# Synthesis, biological evaluation, and computational studies of *N*-benzyl pyridinium–curcumin derivatives as potent AChE inhibitors with antioxidant activity

**DOI:** 10.1080/14756366.2023.2281264

**Published:** 2023-11-20

**Authors:** Nafisah M. Al-Rifai, Nemeh M. Al-Khalileh, Jalal A. Zahra, Musa I. El-Barghouthi, Fouad H. Darras

**Affiliations:** a Pharmaceutical-Chemical Engineering Department, School of Medical Sciences, German Jordanian University, P.O. Box 35247, Amman 11180, Jordan; b Chemistry Department, The University of Jordan, Amman, Jordan; c Department of Chemistry, Faculty of Science, The Hashemite University, Zarqa 13133, Jordan; d Resonance Research Lab, Amman, Jordan

**Keywords:** Acetylcholinesterase inhibitors, antioxidants, Alzheimer’s disease, docking study, synthesis, pyridinium

## Abstract

A library of *N*-benzylpyridinium-based compounds, **7a-j** and **8a-j**, was designed and synthesised as potential acetylcholinesterase) AChE (inhibitors. An *in vitro* assay for the synthesised compounds showed that most compounds had significant AChE inhibitory activities at the nanomolar and submicromolar levels. The benzyl (**8a**) and fluoro (**8b**) derivatives were the most active, with IC_50_ values ≤56 nM. Compound **7f,** which had a benzyl moiety, showed the highest potency among all the target compounds, with an IC_50_ value of 7.5 ± 0.19 nM against AChE, which was higher than that of the activities of tacrine (IC_50_ = 30 ± 0.2 nM) and donepezil (IC_50_ = 14 ± 0.12 nM). Compounds with vanillin moieties exhibited antioxidant activity. Among the tested compounds, four derivatives (**7f**, **7 g**, **8f,** and **8 g**) exhibited superior AChE inhibitory activity, with *K*_i_ values of 6–16 nM, which were potent in the same range as the approved drug, donepezil. These compounds showed moderate antioxidant activities, as indicated by the results of the ABTS assay.

## GRAPHICAL ABSTRACT

**Figure:**
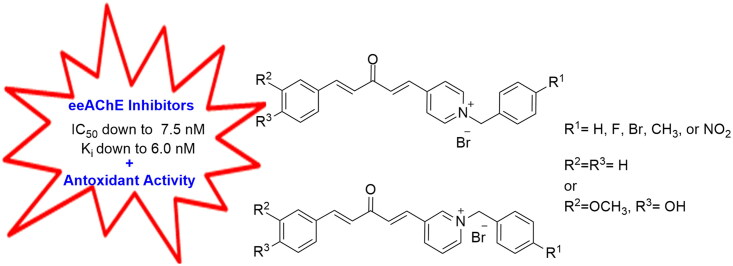

## Introduction

Alzheimer’s disease (AD) is the most common form of dementia reported worldwide. In 2020 and 2021, AD was listed as the seventh leading cause of mortality in the world, making it one of the costliest illnesses in society^1^. Worldwide, there are only four approved drugs for AD, and they only treat the symptoms of moderate stages of the disease, not its cause and progression. Donepezil, rivastigmine, and galantamine treat AD by inhibiting cholinesterase (ChE), whereas memantine is an *N*-methyl-D-aspartate (NMDA) receptor antagonist. In June 2021, the U.S. Food and Drug Administration (USFDA) conditionally approved the antibody Aducanumab (Aduhelm™) as a therapy to address the underlying biology of AD. In January 2023, a new antibody, lecanemab, was approved by the USFDA. However, more studies addressing the efficacy and safety of these drugs are needed to confirm that they have more benefits than risks^2^^,^^3^. These antibodies target amyloid β (Aβ); nevertheless, there are other pathological factors of AD including the discrepancy of acetylcholine (ACh) in the hippocampus and cortex areas of the brain. According to the cholinergic hypothesis, the main pathological changes in AD are triggered by an extreme decline in ACh^4^ levels caused by the structural and functional loss of cholinergic neurons, which accelerates the neurodegenerative process. ACh is a neurotransmitter involved in signal transduction pathways related to memory and learning. Therefore, ACh deficiency leads to a progressive and significant loss of cognitive and behavioural functions in patients with AD^4^. Human AChE is a prominent enzyme responsible for the degradation of ACh, which in turn blocks postsynaptic signal transmission. Additionally, it has been reported that AChE may function by accelerating Aβ formation and could play a role during amyloid deposition in the Alzheimer’s brain^5^^,^^6^. Hence, the approach of designing and synthesising novel AChE inhibitors is clinically valuable, especially if they can bind to both the catalytic active site (CAS) that is hydrophobic and the peripheral anionic site (PAS) of AChE because AChE interacts with β-amyloid through the PAS^7^.

**Figure 1.**
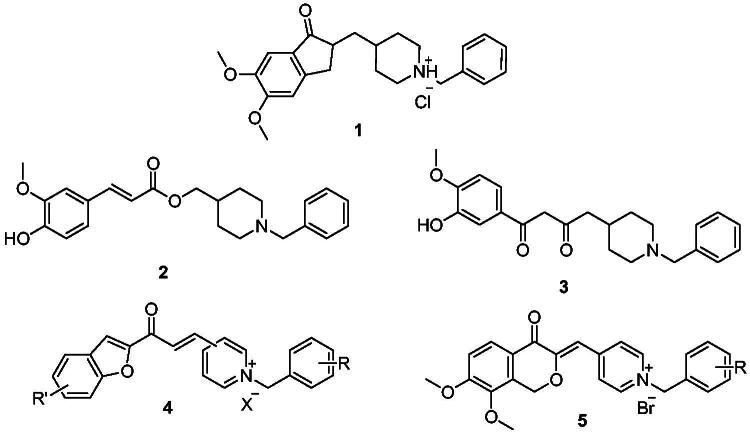
Donepezil (**1**) and reported donepezil analogues that function as AChE inhibitors.

Donepezil is the most potent approved drug for treating AD (**1**, Figure 1), and it significantly impacts several processes related to AD, such as cholinesterase activity, oxidative stress, and anti-Aβ aggregation^8^. Therefore, many AChE inhibitors have been developed using the donepezil structure as a model^7^^,^^8^ (**2**–**5**, Figure 1), a few of which are *N*-benzyl pyridinium compounds, such as compound **4**^9^ and compound **5**^10^.

**Figure 2.**
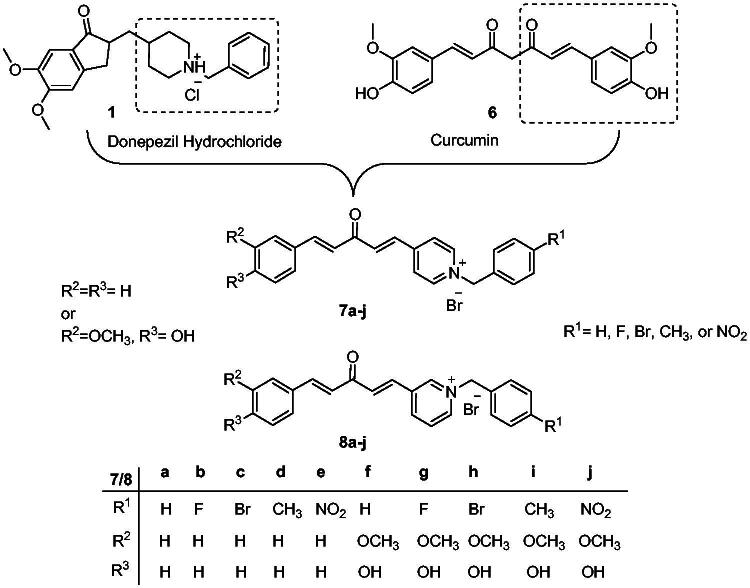
Design strategy of the target compounds.

In the donepezil and AChE-binding model, the benzyl piperidine moiety protonated at physiological pH can interact with CAS, and the indanone part of donepezil binds to PAS. Curcumin is a well-known natural product with many biological activities, including antioxidant and cognitive-enhancing activities^11^. Curcumin has been reported to reduce amyloid formation and inhibit the formation of amyloid β aggregations^12^, and the curcumin part of the compound is thought to interact with the catalytic (hydrophobic) site of AChE. At the same time, the pyridinium moiety will go to the PAS. This is similar to the binding mode of donepezil to AChE, and since curcumin is a known antioxidant, we hypothesised that new analogous compounds derived from curcumin should be AChE inhibitors/antioxidant dual-acting compounds that target the multifactorial pathogenic nature of AD^13^. This strategy is called multi-target directed ligands (MTDLs) and has been reported to be effective in treating AD^14^. We developed a design strategy for the hybrid potential with the activity of the target compounds determined via an *in vitro* AChE inhibition assay using Ellman’s method and an ABTS antioxidant assay (Figure 2).

## Results and discussion

### Chemistry

**Scheme 1.**
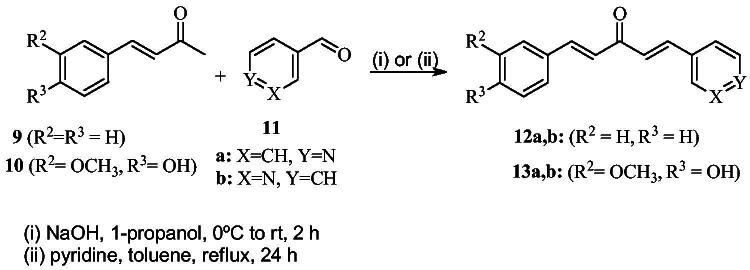
The general route to prepare the α,β-unsaturated carbonyl compounds.

**Scheme 2.**
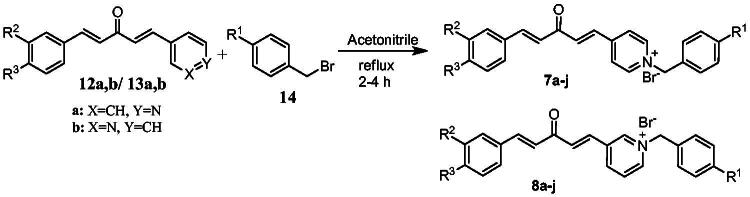
Synthesis of the target benzyl pyridinium salts.

Scheme 1 shows the synthesis of the α,β-unsaturated carbonyl compounds **12a**, **12b**, **13a** and **13b** using the classical base-mediated Claisen–Schmidt condensation of (*E*)-4-phenylbut-3-en-2-one (**9**) or (*E*)-4–(4-hydroxy-3-methoxyphenyl)but-3-en-2-one (**10**) with pyridinecarboxaldehyde **11a** or **11b**. The target benzyl pyridinium salts **7a-j** and **8a-j** were then synthesised by the reaction of *p*-substituted benzyl pyridinium bromides with **12a**, **12b**, **13a**, or **13b** in refluxed acetonitrile^9^ (Scheme 2).

### In vitro AChE and BuChE inhibition

All target compounds, along with the known AD drugs tacrine and donepezil, were tested for their ability to inhibit acetylcholinesterase (*ee*AChE, from *Electrophorus electricus*) and butyrylcholinesterase (*eq*BChE, from equine serum) (Table 1). Both enzymes are widely used to screen compound series because they exhibit high amino acid sequence homology with human orthologs^15^^,^^16^ in addition to their stability and low cost in comparison with human enzymes. The IC_50_ values for the tested compounds were determined, and those with the lowest IC_50_ values were used in kinetic studies to determine the K_i_ values of their inhibition.

**Table 1.** AChE and BChE inhibition, ChEs selectivity, and *K*_i_ values for known and target compounds.

Compound	Chemical Structure	(IC_50_ ± SEM)^a^		Selectivity Index (SI)^b^ (AChE)	(*K_i_* ± SEM)a *ee*AChE (**nM**)
		AChE (**nM**)	BChE (**μM**)		
**Tacrine**	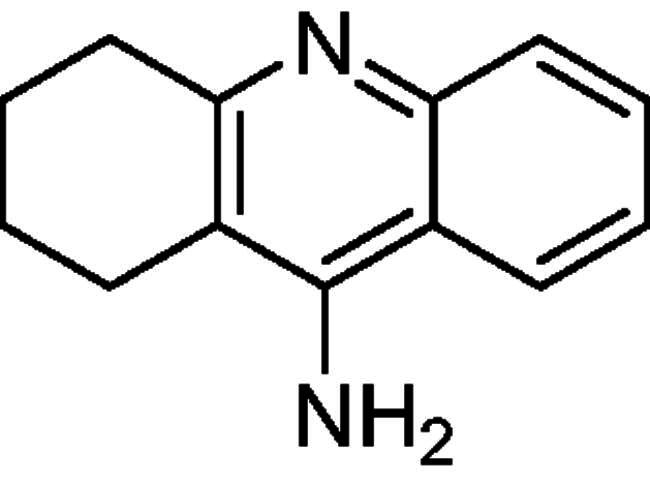	30 ± 0.2	0.003 ± 0.047E-3	0.10	35 ± 0.013
**Donepezil**	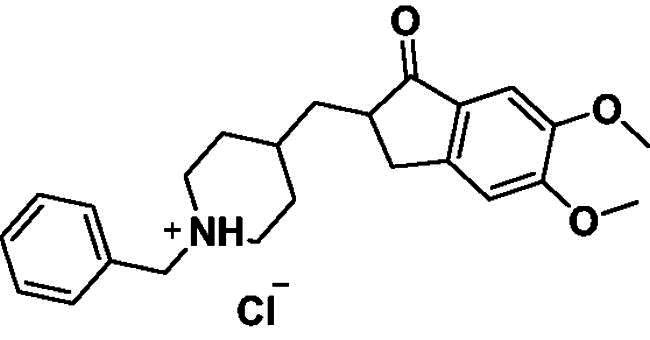	14 ± 0.12	1.4 ± 0.043	101	27 ± 0.018
**7a**	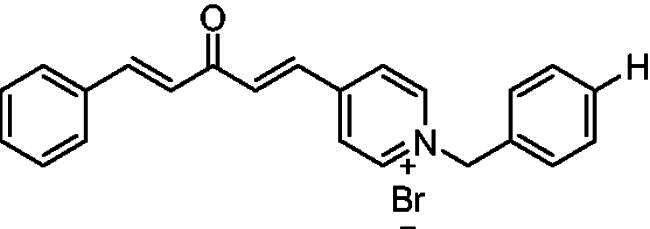	32 ± 0.22	60 ± 0.096	19	39 ± 0.011
**7b**	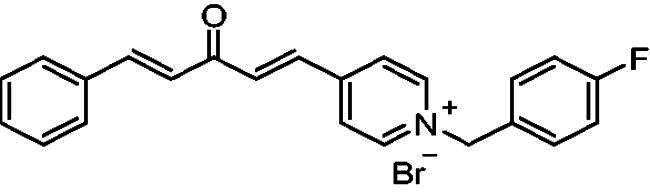	52 ± 0.18	1.07 ± 0.080	21	56 ± 0.016
**7c**	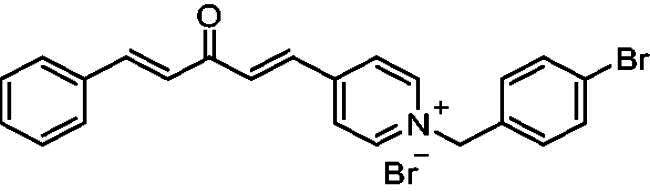	280 ± 0.20	88 ± 0.104	3	−
**7d**	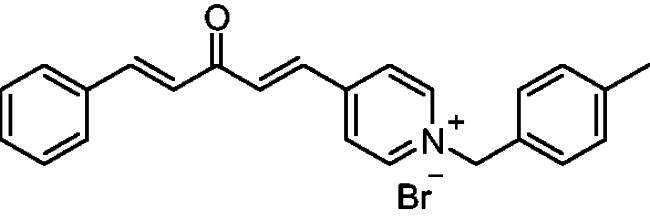	1120 ± 0.14	4.5 ± 0.073	4	−
**7e**	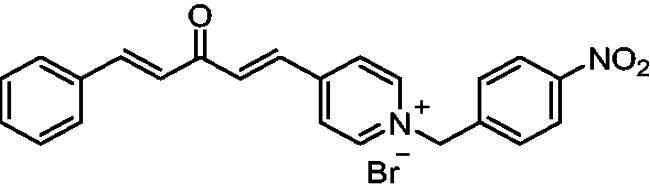	800 ± 0.12	3.9 ± 0.052	5	−
**7f**	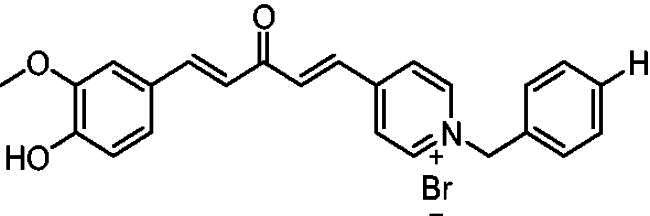	7.5 ± 0.19	94 ± 0.063	55	6.0 ± 0.036
**7 g**	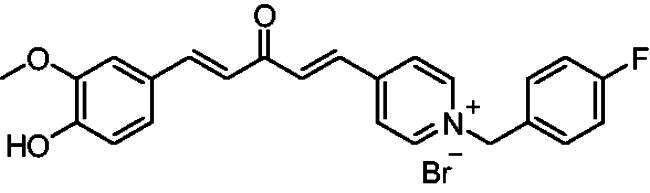	16 ± 0.18	1.04 ± 0.081	65	6.0 ± 0.031
**7h**	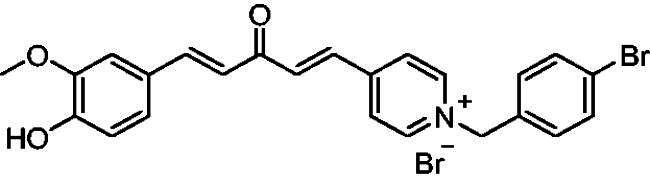	710 ± 0.16	6.36 ± 0.106	10	−
**7i**	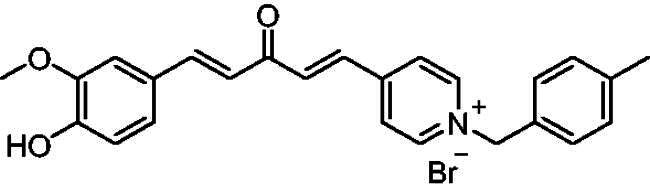	220 ± 0.17	74 ± 0.110	3	−
**7j**	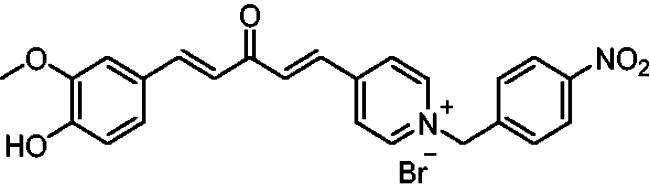	230 ± 0.19	1.4 ± 0.083	2	−
**8a**	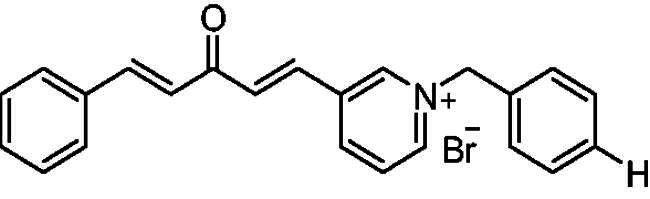	56 ± 0.17	75 ± 0.069	14	102 ± 0.008
**8b**	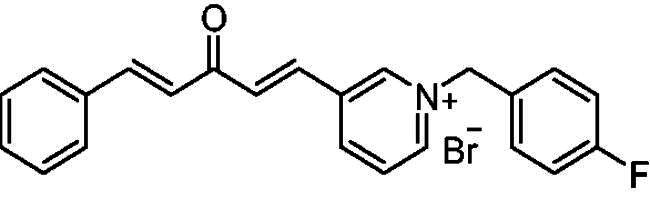	54 ± 0.23	40 ± 0.079	7	90 ± 0.012
**8c**	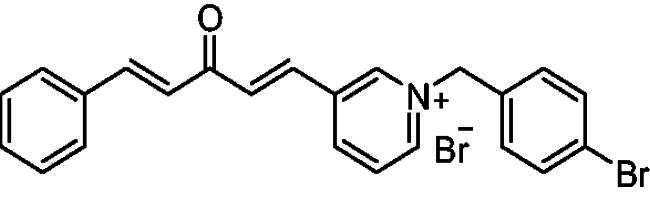	1160 ± 0.17	61 ± 0.076	1	−
**8d**	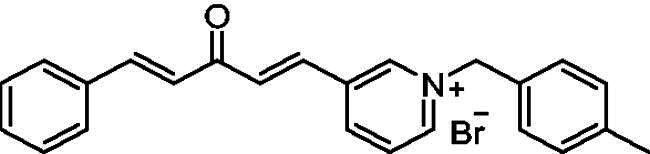	630 ± 0.20	2.5 ± 0.079	4	−
**8e**	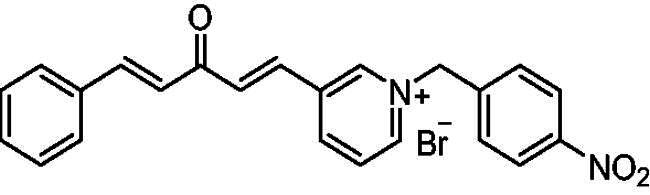	1500 0.22	2.7 ± 0.063	2	−
**8f**	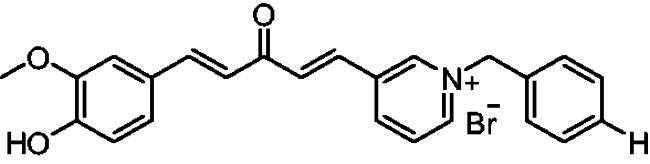	17 ± 0.17	3.5 ± 0.095	473	16 ± 0.022
**8 g**	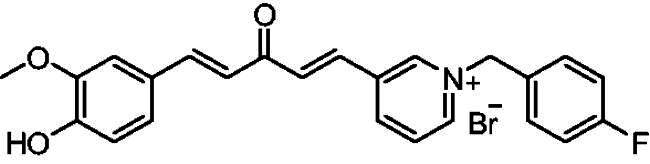	16 ± 0.24	1.2 ± 0.097	79	10 ± 0.025
**8h**	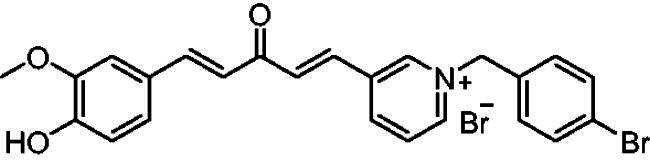	630 ± 0.22	1.8 ± 0.121	3	−
**8i**	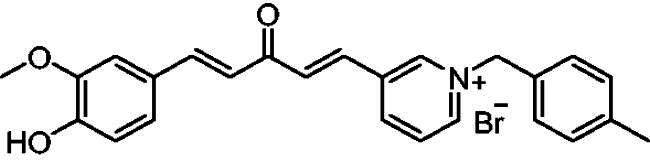	270 ± 021	2.5 ± 0.100	11	−
**8j**	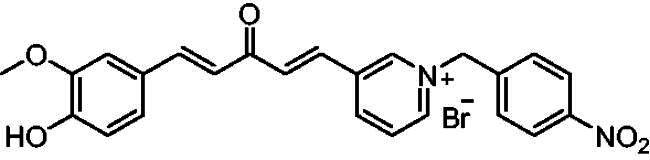	660 ± 0.21	9.8 ± 0.09	42	−

^a^Data are the mean ± standard error of three independent experiments that included duplicate samples.

^b^Selectivity ratio [IC_50_(BChE)/IC_50_(AChE)].

**Figure 3.**
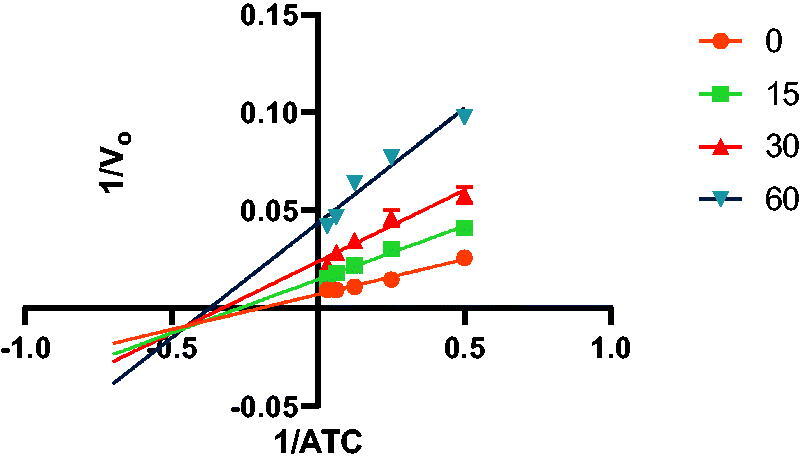
Lineweaver-Burk plot of the substrate-velocity curve of AChE activity with different substrate (ATC) concentrations (32–2 mM) in the absence and presence of compound **7a**.

In addition to determining their inhibitory activities, the most powerful inhibitors, **7a**, **7b**, **7f**, **7 g**, **8a**, **8b**, **8f,** and **8 g,** were used for kinetic studies to determine their *K_i_* values, and double reciprocal Lineweaver-Burk plots were produced (provided in the SI). Figure 3 shows the Lineweaver-Burk plot of compound **7a**, illustrating that as the inhibitor concentration increased, the slopes and intercepts increased. This pattern indicated a mixed-type inhibition, suggesting that the inhibitor may be able to bind to either the free enzyme or the enzyme-substrate complex, similar to donepezil^17^ and tacrine^18^.

Structurally, compounds **7a-j** and **8a-j** were 4-pyridinium and 3-pyridinium derivatives, respectively. Derivatives **7f-j** and **8j-f** have curcumin residues in their structure. At the same time, the two basic 4-pyridinium (**7a**) and 3-pyridinium compounds (**8a**) were potent AChE inhibitors (IC_50_ = 32 nM and 56 nM, respectively). The potencies of the parent curcumin 4-pyridinium (**7f**) and curcumin 3-pyridinium compounds (**8f**) were even higher, with IC_50_ values of 7.5 and 17 nM, respectively. Derivative **7f** had an unsubstituted benzyl moiety and was the most potent among all the target compounds, with an IC_50_ value of 7.5 ± 0.19 nM that was higher than that of tacrine (IC_50_ = 30 ± 0.2 nM) and donepezil (IC_50_ = 14 ± 0.12 nM). Substitution at the benzyl residue is disputable and can be attributed to the size of the substituent. Compound **7 g** had the smallest substituent F and an IC_50_ value of 16 ± 0.18 nM, which was close to that of **7f**. The same behaviour was observed for compounds **8f** and **8 g**. While derivatives with nitro, bromo, and methyl substituents show lower anti-AChE activity with IC_50_ values in the 0.22 to 1.5 µM range, these compounds were less potent inhibitors of AChE than those with H or F substituents.

Most of the target compounds exhibited high inhibitory activities against *ee*AChE, with IC_50_ values in the nano- and low-micromolar ranges. All the target compounds showed distinct AChE selectivity over BChE, considering that donepezil exhibited higher selectivity for AChE over BChE, whereas tacrine was more selective for BChE^19^. Furthermore, it has been noticed that 3- and 4-pyridinium derivatives with similar substituents (R) have similar potency against AChE. The dose-response curves and hillslope values of all tested compounds are provided in the Supporting Information.

**Figure 4.**
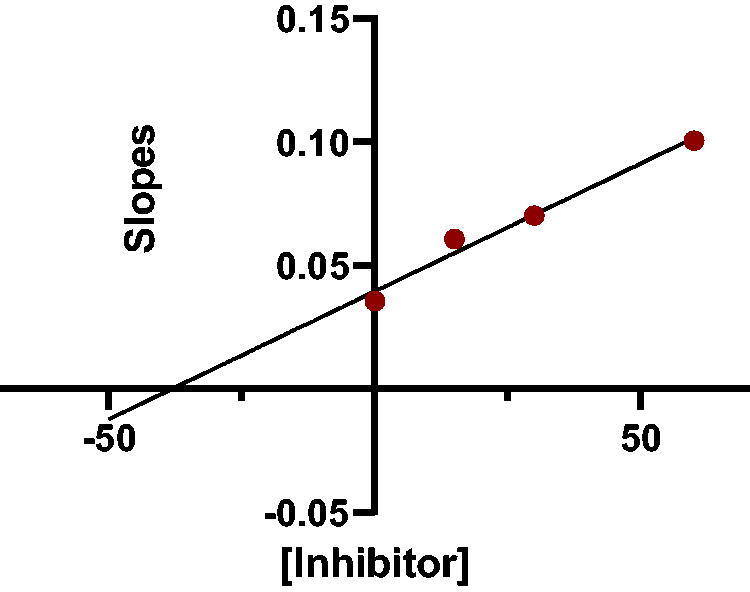
Slopes of Lineweaver–Burk plots versus inhibitor **7a** concentration.

The slopes of the Lineweaver-Burk reciprocal plots against the concentrations of the compound (inhibitor) were plotted to obtain the *K_i_* values for the prepared compounds (Table 1). The value at which the line intersects the x-axis is -*K_i_*. The plot of slopes of the Lineweaver-Burk plots versus inhibitor **7a** concentrations is shown in Figure 4 (-*K_i_* = −39 nM).

### In vitro antioxidant activity

The ABTS assay was performed to determine the antioxidant activity of the most potent AChE inhibitors that were synthesised. The radical scavenging activities of the target compounds were compared to the reference antioxidants, trolox and α-tocopherol, at the same concentration and expressed as a percentage of inhibition against ABTS (2,2′-azino-bis(3-ethylbenzothiazoline-6-sulfonic acid). Compounds with a curcumin moiety exhibited good-to-moderate antioxidant activity in a concentration-dependent manner (Table 2). The radical scavenging activity of compound **7f** at 0.5 mg/mL was 55.3%. In contrast, compounds **8f** and **8 g** had approximately 59% activity at the same concentration. Trolox and α-tocopherol exhibited 93.9 and 90.2% scavenging activity, respectively, at the same concentration of the target compounds.

**Table 2.** ABTS antioxidant activity.

	Inhibition %				
	Concentrations (mg/mL)				
**Compounds**	0.0312 mg/mL	0.0625 mg/mL	0.125 mg/mL	0.25 mg/mL	0.5 mg/mL
Trolox	9.3 ± 1.2	24.7 ± 1.2	51.0 ± 2.3	91.5 ± 1.8	93.9 ± 0.1
α-tocopherol	6.9 ± 2.3	15.9 ± 1.0	29.7 ± 1.3	56.4 ± 0.72	90.2 ± 0.04
**7a**	1.4 ± 0.56	1.5 ± 0.30	1.7 ± 0.68	3.5 ± 0.72	6.4 ± 0.47
**7b**	0.5 ± 0.47	1.4 ± 0.07	3.3 ± 0.10	6.2 ± 0.50	7.1 ± 0.59
**8a**	0.0 ± 0.70	0.3 ± 0.28	0.4 ± 0.36	1.4 ± 0.49	2.6 ± 0.38
**8b**	0.2 ± 0.40	0.3 ± 0.22	1.0 ± 0.52	2.3 ± 0.64	5.1 ± 0.62
**7f**	5.9 ± 0.47	13.7 ± 0.50	23.7 ± 0.51	35.5 ± 0.93	55.3 ± 1.42
**7 g**	6.1 ± 0.82	13.9 ± 0.68	22.5 ± 0.77	30.2 ± 1.86	52.1 ± 1.58
**8f**	7.7 ± 0.97	15.8 ± 0.18	25.4 ± 0.57	39.3 ± 0.23	59.1 ± 0.15
**8 g**	6.1 ± 0.71	13.7 ± 0.52	24.7 ± 0.51	39.5 ± 0.38	59.0 ± 0.71

As can be concluded from the results of both Ellman’s and ABTS assays, there was a good correlation between the inhibitory activity of the target compounds and their radical scavenging activity, which supported the multi-target behaviour of these compounds. In other words, they can simultaneously target the cholinergic system and oxidative stress in the brain.

### Docking study with AChE

**Figure 5.**
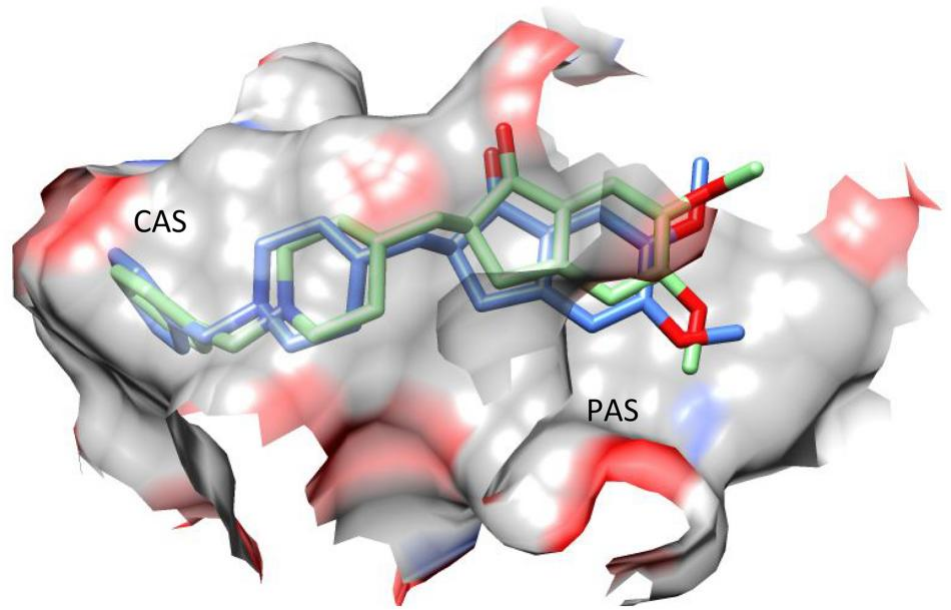
Superposition of the X-ray (blue) and docked (light green) structures of donepezil within the active site of AChE.

The results of the *in vitro* AChE inhibition assay for the synthesised target compounds motivated us to investigate and confirm their molecular interactions with AChE. Therefore, molecular docking techniques were applied to a few of the target compounds to investigate the intermolecular forces involved in the recognition process, using the interaction between donepezil and AChE for comparison. Two binding sites comprise the AChE structure: the peripheral anionic site (PAS) at the enzyme entrance and the catalytic active site (CAS) at the bottom of the gorge^10^. Donepezil’s docking results showed that it interacted with both CAS and PAS (Figure 5).

**Figure 6.**
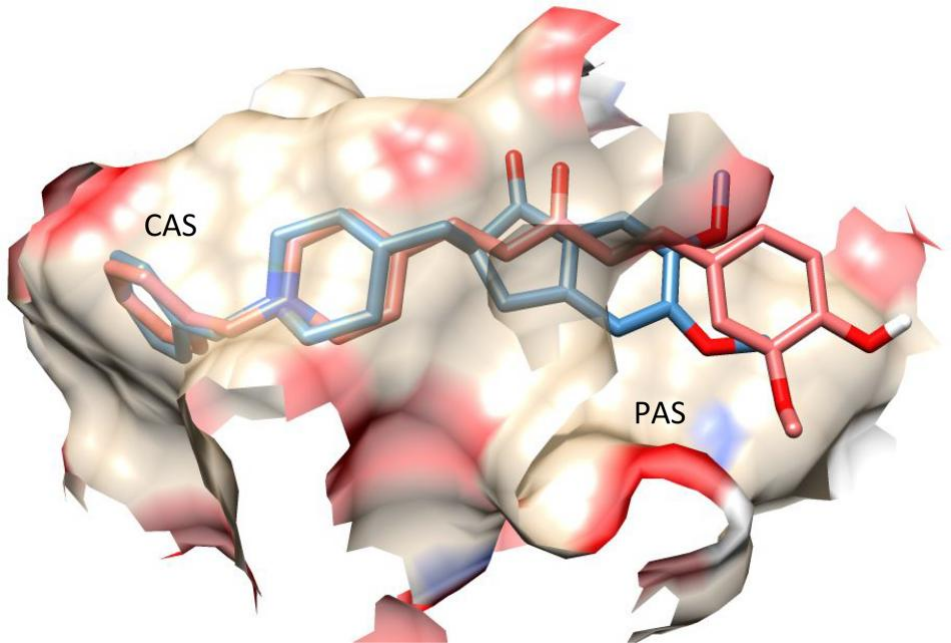
Superposition of the top-scored structure of target compound **7f** (pink) and the X-ray structure of donepezil (blue) within the active site of AChE.

The most potent compound in our library was **7f**, which had a binding environment similar to that of donepezil (IC_50_ = 19 nM)^19^, with similar binding positions observed in both the CAS and PAS structures (Figure 6).

**Figure 7.**
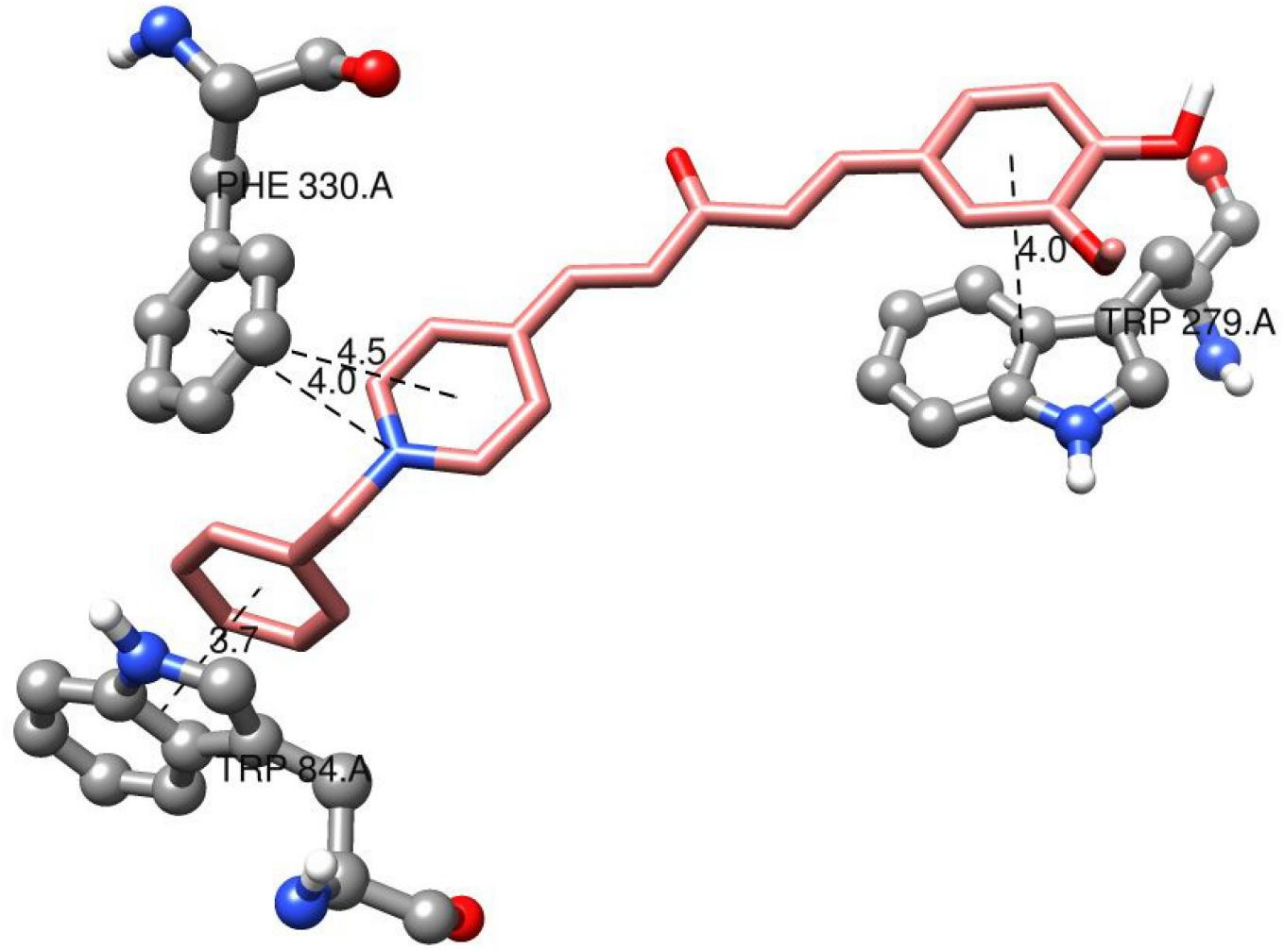
Interaction mode of the target compound **7f** in the active site of ACh.

The amino acids TRP84 and PHE330 were close to the bottom of the gorge. They exhibited positive interactions with compound **7f**, as observed from the docked structure of compound **7f** with AChE (Figure 7). TRP84 was stacked with the benzene aromatic ring of compound **7f** over a 3.7 Å-ring-to-ring distance. The quaternary nitrogen of the pyridinium ring in compound **7f** interacted with the phenyl group of PHE330 at a distance of 4.0 Å. At a distance of 4.5 Å, an aromatic interaction was observed between the pyridinium ring of **7f** and the phenyl group of PHE330. In the PAS site, the vanillin moiety of compound **7f** interacted with the indole ring of TRP279 via a stacking interaction with a distance of 4.0. These results illustrated that compound **7f** could simultaneously interact with the CAS and PAS of AChE.

**Figure 8.**
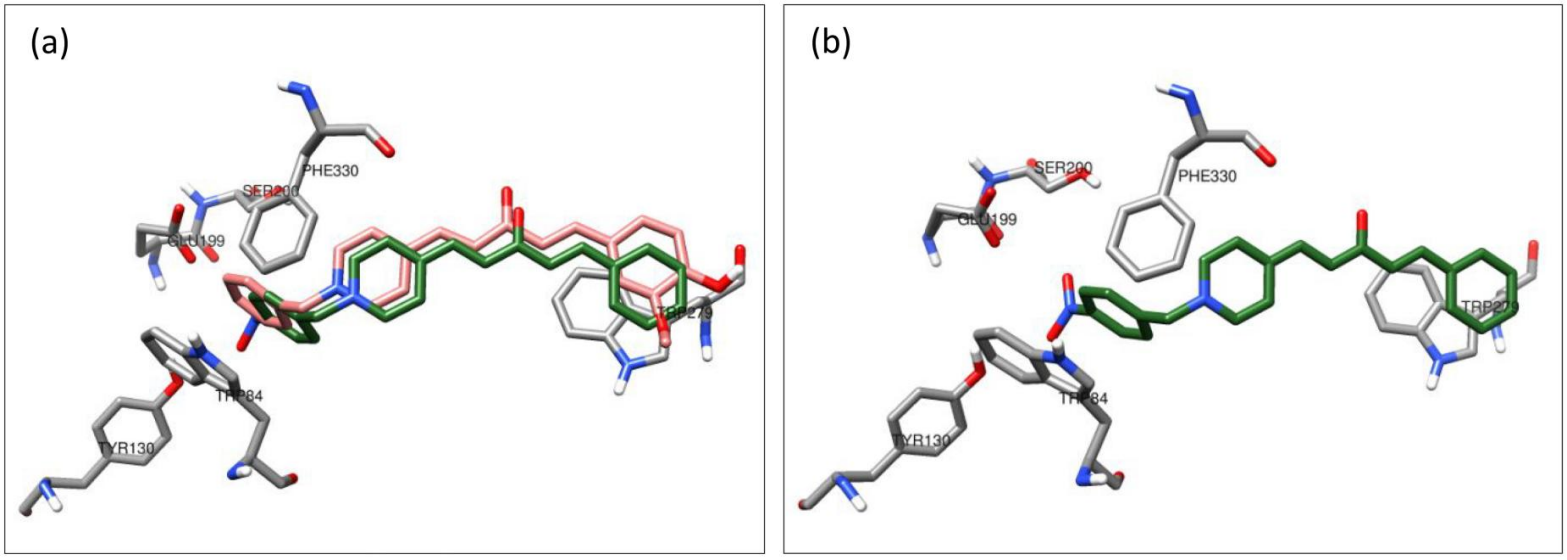
(a) Superposition of the top-scored structures for compounds **7e** (green) and **7f** (pink) within the active site of AChE. (b) Interaction mode of compound **7e** in the active site of AChE.

The AChE inhibitory activity assay for target compound **7e** demonstrated a noticeable decrease in inhibitory affinity, with an IC_50_ of 0.8 μM compared to 7.5 nM for **7f**. Therefore, a molecular docking study of the 4-nitrobenzyl analog compound **7e** was performed to understand this behaviour. The superposition of the top-scoring structures for compounds **7e** and **7f** showed that compound **7e** could interact with the surrounding amino acids similar to that of compound **7f** (Figure 8). The superimposition of the two docked structures showed that the orientation of compound **7e** shifted, affecting the strength of its interaction with the amino acids of AChE. Additionally, the close proximity of the nitro group in compound **7e** to the carboxylate group of GLU199 induced an orientational change in the benzyl moiety in compound **7f**. This may explain the reduced activity of compound **7e** compared to that of compound **7f.**

The effect of the size of the four-substituent group on the binding affinity towards the CAS in AChE may explain the reduction in AChE inhibitory activity of all bulky nitro, bromo, and methyl groups versus para H and/or F analogs as a result of this docking study.

## Experimental

### Chemistry

All chemicals were purchased from commercial sources: Sigma-Aldrich Co. and Acros. Melting points of the compounds were uncorrected and measured in capillary tubes, using Krüss Optronic melting point metre M3000. ^1^H and ^13^C-NMR spectral data were obtained from a Bruker Avance III spectrometer (500 and 125 MHz, respectively) with TMS as an internal standard. Spectra were acquired in deuterated dimethyl sulfoxide-*d*_6_ (DMSO-*d*_6_) or Methanol-*d*_4_ (CD_3_OD). ^1^H-NMR and ^13^C-NMR spectra of all compounds are given in SI (Figure S1-S48). High-resolution mass spectra (HRMS) were measured (in positive ion mode) using electrospray ion trap (ESI) technique by collision-induced dissociation on a Bruker APEX-IV (7 Tesla) instrument. TLC was performed on silica gel on aluminium foils with fluorescent indicator 254 nm (Sigma-Aldrich). For column chromatography, silica gel 60, 230–400 mesh (Macherey-Nagel) was used.

#### General procedure for the synthesis of compounds 12a and 12b

To a mixture of (*E*)-4-phenylbut-3-en-2-one (**9**) (20 mmol) and pyridinecarboxaldehyde **11a** or **11b** (20 mmol) in toluene (10 ml), 7–10 drops of piperidine were added. Then, the reaction mixture was refluxed for 24 h (overnight). After completion of the reaction (monitored by TLC using a 1:1 *n*-hexane/ethyl acetate eluent system), the mixture was cooled and then concentrated under vacuum. The residue was subjected to column chromatography using *n*-hexane/ethyl acetate (1:1) as the eluent system to give a yellow solid product (**12a** or **12b**).

##### (1*E,*4*E*)-1-Phenyl-5-(pyridin-4-yl)penta-1,4-dien-3-one (12a)

Yellow solid, Yield: 21%. ^1^H- NMR (500 MHz, Methanol-*d*_4_) *δ*: 8.56 (d, *J* = 5.1 Hz, 2H), 7.81 (d, *J* = 16.0 Hz, 1H; H-aliphatic), 7.68 (m, 5H), 7.48 (d, *J* = 16.1 Hz, 1H; H-aliphatic), 7.41 (*m*, 3H), 7.21 (d, *J* = 16.1 Hz, 1H; H-aliphatic) ppm. ^13^C-NMR (125 MHz, Methanol-*d*_4_) *δ*: 189.3 (C = O), 149.5 (CH), 144.6 (CH), 143.4, 139.5 (CH), 134.5, 130.6 (CH), 129.5 (CH), 128.7 (CH), 128.3 (CH), 124.9 (CH), 122.5 (CH) ppm. HRMS (ESI) *m/z*: calcd for C_16_H_14_NO, [M + H]^+^: 236.10307, found: 236.10606.

##### (1*E,*4*E*)-1-phenyl-5-(pyridin-3-yl)penta-1,4-dien-3-one (12b)

Yellow solid, Yield: 15%. ^1^H NMR (500 MHz, DMSO-*d*_6_) *δ*: 8.91 (d, *J* = 2.2 Hz, 1H), 8.57 (*m*, 1H), 8.19 (*m*, 1H), 7.77 (*m*, 4H), 7.57 (*m*, 1H), 7.48 (*m*, 2H), 7.43 (*m*, 2H) 7.29 (d, *J* = 16.2 Hz, 1H, H-aliphatic) ppm. ^13^C NMR (125 MHz, DMSO-*d*_6_) *δ*: 188.8 (C = O), 151.4 (CH), 150.5 (CH), 143.7 (CH), 139.8 (CH), 135.2 (CH), 135.1, 131.1 (CH), 131.0, 129.4 (CH), 129.0 (CH), 127.6 (CH), 126.1 (CH), 124.4 (CH) ppm. HRMS (ESI) *m/z*: calcd for C_16_H_14_NO, [M + H]^+^: 236.10699, found: 236.10780.

#### General procedure for the synthesis of compounds 13a and 13b

(*E*)-4–(4-hydroxy-3-methoxyphenyl)but-3-en-2-one (**10**) (5.2 mmol) and pyridinecarboxaldehyde **11a** or **11b (**20 mmol) were dissolved in 1-propanol (15 ml). The resulting solution was stirred at room temperature for 10 min; then the reaction mixture was cooled in an ice-bath and 1 M NaOH (3 mmol) was slowly added. The stirring continued at room temperature for about 2 h. After completion of the reaction as illustrated by TLC (using 1:1 ethyl acetate/*n*-hexane eluent system), an appropriate amount of ice-cooled water (25 ml) was added, and the pH was adjusted to about 5–6 using 5% HCl solution^10^. The yellow precipitate was filtered and washed with diethyl ether furnishing **13a** or **13b**.

##### (1*E,*4*E*)-1–(4-Hydroxy-3-methoxyphenyl)-5-(pyridin-4-yl)penta-1,4-dien-3-one (13a)

Yellow solid, Yield: 92%. ^1^H-NMR (500 MHz, DMSO-*d*_6_) *δ*: 9.75 (*s*, 1H, OH), 8.65 (*m*, 2H), 7.79 (d, *J* = 16.0 Hz, 1H), 7.72 (*m*, 2H), 7.65 (d, *J* = 16.0 Hz, 1H), 7.59 (d, *J* = 16.0 Hz, 1H), 7.39 (d, *J* = 2.0 Hz, 1H), 7.24 (*m*, 1H), 7.14 (d, *J* = 15.9 Hz, 1H), 6.86 (*m*, 1H), 3.85 (*s*, 3H, OCH_3_) ppm. ^13^C-NMR (125 MHz, DMSO-*d*_6_) *δ*: 188.61 (C = O), 150.8 (CH), 150.3, 148.4, 145.1 (CH), 142.5, 139.4 (CH), 129.9 (CH), 126.5, 124.2 (CH), 123.3 (CH), 122.6 (CH), 116.2 (CH), 112.0 (CH), 56.1 (OCH_3_) ppm. HRMS (ESI) *m/z*: calcd for C_17_H_16_NO_3_, [M + H]^+^: 282.11247, found: 282.11414.

##### (1*E,*4*E*)-1–(4-Hydroxy-3-methoxyphenyl)-5-(pyridin-3-yl)penta-1,4-dien-3-one (13b)

Yellow solid, Yield: 90%. ^1^H-NMR (500 MHz, DMSO-*d*_6_) *δ*: 9.68 (*s*, 1H, OH), 8.90 (d, *J* = 2.2 Hz, 1H), 8.56 (*m*, 1H), 8.18 (*m*, 1H), 7.74 (d, *J* = 16.0 Hz, 1H, H-aliphatic), 7.68 (d, *J* = 16.1 Hz, 1H, H-aliphatic), 7.47 (*m*, 1H), 7.43 (*m*, 1H), 7.34 (d, *J* = 2.0 Hz, 1H), 7.18 (*m*, 1H), 7.08 (d, *J* = 16.0 Hz, 1H, H-aliphatic), 6.81 (d, *J* = 8.1 Hz, 1H), 3.81 (*s*, 3H, OCH_3_) ppm. ^13^C-NMR (125 MHz, DMSO-*d*_6_) *δ*: 188.5 (C = O), 151.2 (CH), 150.4 (CH), 150.2 (CH), 148.4 (CH), 144.7 (CH), 138.8 (CH), 135.1, 131.1 (CH), 127.6, 126.6 (CH), 124.4, 124.0 (CH), 123.4 (CH), 116.1 (CH), 112.0 (CH), 56.1 (OCH_3_) ppm. HRMS (ESI) *m/z*: calcd for C_17_H_16_NO_3_, [M + H]^+^: 282.11247, found: 282.11444.

#### General procedure for the synthesis of pyridinium salt derivatives (7a-j, 8a-j)^10^

Compound **12a, 12b, 13a** or **13b** (0.3 mmol) was dissolved in acetonitrile (5–6 ml) upon heating. Then, benzyl bromide or substituted benzyl bromide (0.6 mmol) was added dropwise. The reaction mixture was heated under reflux for 2–4 h. After completion of the reaction as indicated by TLC using 10:1 dichloromethane/methanol as an eluent system, the resulting yellow solid was filtered and washed with diethyl ether to afford the targeted pyridinium salts **(7a-j, 8a-j)**.

##### 1-Benzyl-4-(1*E,*4*E*)-3-oxo-5-phenylpenta-1,4-dien-1-yl)pyridin-1-ium bromide (7a)

Yellow solid, Yield: 73%, mp 191–193 ^0^C. ^1^H-NMR (500 MHz, DMSO-*d*_6_) *δ*: 9.24 (d, *J* = 6.4 Hz, 2H, H-pyridine), 8.49 (d, *J* = 6.3 Hz, 2H, H-pyridine), 7.94 (dd, *J* = 16.2 Hz, 2H, H-aliphatic), 7.79 (*m*, 3H), 7.52 (*m*, 2H), 7.43 (*m*, 6H), 7.27 (d, *J* = 16.2 Hz, 1H, H-aliphatic), 5.81 (*s*, 2H, N-CH_2_) ppm. ^13^C-NMR (125 MHz, DMSO-d6) *δ*: 188.7 (C = O), 151.5, 145.5 (CH), 145.5 (CH), 136.3 (CH), 135.2 (CH), 134.8 (CH), 134.8, 131.5 (CH), 129.8 (CH), 129.7 (CH), 29.6 (CH), 129.2 (CH), 129.2 (CH), 126.9 (CH), 126.2 (CH), 63.4 (N-CH_2_) ppm. HRMS (ESI) *m/z*: calcd for C_23_H_20_NO^+^, [M]^+^: 326.15394, found: 326.15558.

##### 1–(4-Fluorobenzyl)-3-(1*E,*4*E*)-3-oxo-5-phenylpenta-1,4-dien-1-yl)pyridin-1-ium bromide (7b)

Yellow solid, Yield: 76% and mp 119–122 ^0^C. ^1^H-NMR (500 MHz, DMSO-*d*_6_) *δ*: 9.25 (m, 2H, H-pyridine), 8.49 (m, 2H, H-pyridine), 7.95 (d, *J* = 16.2 Hz, 2H, H-aliphatic), 7.80 (*m*, 4H), 7.64 (*m*, 2H), 7.45 (*m*, 3H), 7.25 (*m*, 2H), 5.81 (*s*, 2H, N-CH_2_) ppm. ^13^C-NMR (125 MHz, DMSO-*d*_6_) *δ*: 188.7 (C = O), 163.0 (d, *J* = 246.68 Hz, C-F), 151.5, 145.6 (CH), 145.4 (CH), 136.3, 135.2 (CH), 134.8 (CH), 131.9 (d, *J* = 8.7 Hz), 131.5 (CH), 131.0 (d, *J* = 3.2 Hz), 129.5 (CH), 129.2 (CH), 127.0 (CH), 126.2 (CH), 116.6 (d, *J* = 22.68 Hz,), 62.5 (CH_2_) ppm. HRMS (ESI) *m/z*: calcd for C_23_H_19_FNO^+^, [M]^+^: 344.14452, found: 344.14651.

##### 1–(4-Bromobenzyl)-4-(1*E,*4*E*)-3-oxo-5-phenylpenta-1,4-dien-1-yl)pyridin-1-ium bromide (7c)

Yellow solid, Yield: 61% and mp 100–102 ^0^C. ^1^H-NMR (500 MHz, DMSO-*d*_6_) *δ*: 9.21 (d, *J* = 6.3 Hz, 2H, H-pyridine), 8.48 (d, *J* = 6.3 Hz, 2H, H-pyridine), 7.93 (dd, *J* = 16.1 Hz, 2H, aliphatic-H), 7.76 (*m*, 3H), 7.64 (d, *J* = 8.4 Hz, 2H), 7.47 (*m*, 5H), 7.27 (d, *J* = 16.2 Hz, 1H, aliphatic-H), 5.79 (*s*, 2H, N-CH_2_) ppm. ^13^C-NMR (125 MHz, DMSO-*d*_6_) *δ*: 188.1 (C = O), 162.2, 151.0, 145.0 (CH), 144.9 (CH), 135.7 (CH), 134.6 (CH), 134.2, 133.4, 132.0, 131.0 (CH), 129.0 (CH), 128.6 (CH), 126.4 (CH), 125.6 (CH), 122.7 (CH), 61.9 (N-CH_2_) ppm. HRMS (ESI) *m/z*: calcd for C_23_H_19_BrNO^+^, [M]^+^: 404.06445, 406.06241, found: 404.06282 (^79^Br), 406.06098 (^81^Br).

##### 1–(4-Methylbenzyl)-4-(1*E,*4*E*)-3-oxo-5-phenylpenta-1,4-dien-1-yl)pyridin-1-ium bromide (7d)

Yellow solid, Yield: 76%, decomposed at 195–197 °C. ^1^H NMR (500 MHz, DMSO-*d*_6_) *δ*: 9.25 (d, *J* = 6.3 Hz, 2H, pyridine-H), 8.50 (d, *J* = 6.3 Hz, 2H, pyridine-H), 7.99 (d, *J* = 2.9 Hz, 1H), 7.95 (d, *J* = 3.1 Hz, 1H), 7.80 (*m*, 3H), 7.44 (*m*, 5H), 7.27 (d, *J* = 16.2 Hz, 1H, aliphatic-H), 7.22 (d, *J* = 7.8 Hz, 2H), 5.78 (*s*, 2H, N-CH_2_), 2.26 (*s*, 3H, CH_3_) ppm. ^13^C NMR (125 MHz, DMSO-*d*_6_) *δ*: 188.8 (C = O), 151.4, 145.6 (CH), 145.3 (CH), 139.4, 136.3 (CH), 135.1 (CH), 134.8, 131.8, 131.5 (CH), 130.2 (CH), 129.5 (CH), 129.3 (CH), 129.2 (CH), 126.9 (CH), 126.2 (CH), 63.1 (N-CH_2_), 21.2 (CH_3_) ppm. HRMS (ESI) *m/z*: calcd for C24H22NO^+^, [M]^+^: 340.16959, found: 340.16795.

##### 1–(4-Nitrobenzyl)-4-(1*E,*4*E*)-3-oxo-5-phenylpenta-1,4-dien-1-yl)pyridin-1-ium bromide (7e)

Yellow solid, Yield: 72% and decomposed at 210 ^0^C. ^1^H-NMR (500 MHz, DMSO-*d*_6_) *δ*: 9.27 (d, *J* = 6.3 Hz, 2H, pyridine-H), 8.54 (d, *J* = 6.3 Hz, 2H, pyridine-H), 8.26 (d, *J* = 8.4 Hz, 2H), 7.97 (dd, *J* = 15.5 Hz, 2H, aliphatic-H), 7.83 (d, *J* = 16.0 Hz, 1H, aliphatic-H), 7.77 (*m*, 4H), 7.45 (*m*, 3H), 7.27 (d, *J* = 16.2 Hz, 1H, aliphatic-H), 5.99 (*s*, 2H, N-CH_2_) ppm. ^13^C-NMR (125 MHz, DMSO-*d*6) *δ*: 188.1 (C = O), 151.2, 147.8, 145.3 (CH), 145.0 (CH), 141.1, 135.7 (CH), 134.7 (CH), 134.2, 131.0 (CH), 129.9 (CH), 129.0 (CH), 128.6 (CH), 126.5 (CH), 125.6 (CH), 124.0 (CH), 61.6 (N-CH_2_) ppm. HRMS (ESI) *m/z*: calcd for C_23_H_19_N_2_O_3_^+^, [M]^+^: 371.13902, found: 371.14106.

##### 1-Benzyl-4-(1*E,*4*E*)-5–(4-hydroxy-3-methoxyphenyl)-3-oxopenta-1,4-dien-1-yl)pyridin-1-ium bromide (7f)

Orange solid, Yield: 78% and decomposed at 182–183 ^0^C. ^1^H-NMR (500 MHz, DMSO-*d*_6_) *δ*: 9.80 (s, 1H, OH), 9.22 (d, *J* = 6.3 Hz, 2H, pyridine-H), 8.47 (d, *J* = 6.3 Hz, 2H, pyridine-H), 7.94 (d, *J* = 16.0 Hz, 1H, aliphatic-H), 7.86 (d, *J* = 16.1 Hz, 1H, aliphatic-H), 7.74 (d, *J* = 15.9 Hz, 1H, aliphatic-H), 7.52 (*m*, 2H), 7.38 (*m*, 4H), 7.21 (*m*, *J* = 8.2 Hz, 1H), 7.09 (d, *J* = 16.1 Hz, 1H), 6.82 (d, *J* = 8.1 Hz, 1H), 5.80 (*s*, 2H, N-CH_2_), 3.81 (*s*, 3H, OCH_3_) ppm. ^13^C-NMR (125 MHz, DMSO-*d*_6_) *δ*: 188.2 (C = O), 151.6, 150.7, 148.5, 146.5 (CH), 145.4 (CH), 135.5 (CH), 135.4 (CH), 134.8, 129.8 (CH), 129.7 (CH), 129.2 (CH), 126.9 (CH), 126.3, 124.5 (CH), 123.4 (CH), 116.2 (CH), 112.0 (CH), 63.3 (N-CH_2_), 56.2 (OCH_3_) ppm. HRMS (ESI) *m/z*: calcd for C_24_H_22_NO_3_^+^, [M]^+^: 372.15942, found: 372.16075.

##### 1–(4-Fluorobenzyl)-4-(1*E,*4*E*)-5–(4-hydroxy-3-methoxyphenyl)-3-oxopenta-1,4-dien-1-yl)pyridin-1-ium bromide (7 g)

Maroon solid, Yield: 77% and decomposed at 222–224 ^0^C. ^1^H-NMR (500 MHz, DMSO-*d*_6_) *δ*: 9.80 (s, 1H, OH), 9.20 (d, *J* = 6.3 Hz, 2H, pyridine-H), 8.46 (d, *J* = 6.3 Hz, 2H, pyridine-H), 7.85 (d, *J* = 16.1 Hz, 1H, aliphatic-H), 7.73 (d, *J* = 15.9 Hz, 1H, aliphatic-H), 7.62 (*m*, 2H), 7.35 (d, *J* = 2.0 Hz, 1H,), 7.32 (*s*, 1H), 7.24 (*m*, 3H), 7.09 (d, *J* = 16.0 Hz, 1H, aliphatic-H), 6.82 (d, *J* = 8.1 Hz, 1H), 5.78 (*s*, 2H, N-CH_2_), 3.81 (*s*, 3H, O-CH_3_) ppm. ^13^C-NMR (125 MHz, DMSO-*d*_6_) *δ*: 188.2 (C = O) 163.0 (d, *J* = 247.19 Hz, C-F), 151.7, 150.7, 146.5 (CH), 145.4 (CH), 135.5 (d, *J* = 13.8 Hz, CH), 131.8 (d, *J* = 8.7 Hz), 131.0, 126.9 (CH), 126.3, 124.5 (CH), 123.4 (CH), 116.6 (d, *J* = 21.7 Hz, CH), 116.2 (CH), 112.0 (CH), 62.5 (N-CH_2_), 56.2 (O-CH_3_) ppm. HRMS (ESI) *m/z*: calcd for C_24_H_21_FNO_3_^+^, [M]^+^: 390.15000, found: 390.14826.

##### 1–(4-Bromobenzyl)-4-(1*E,*4*E*)-5–(4-hydroxy-3-methoxyphenyl)-3-oxopenta-1,4-dien-1-yl)pyridin-1-ium bromide (7h)

Maroon solid, Yield: 89% and decomposed at 234–235 ^0^C. ^1^H- NMR (500 MHz, DMSO-*d*_6_) *δ*: 9.79 (s, 1H, OH), 9.21 (d, *J* = 6.4 Hz, 2H, pyridine-H), 8.48 (d, *J* = 6.4 Hz, 2H, pyridine-H), 7.96 (d, *J* = 16.0 Hz, 1H, aliphatic-H), 7.87 (d, *J* = 16.0 Hz, 1H, aliphatic-H), 7.74 (d, *J* = 15.9 Hz, 1H, aliphatic-H), 7.63 (d, *J* = 8.2 Hz, 2H), 7.49 (d, *J* = 8.2 Hz, 2H), 7.36 (d, *J* = 2.0 Hz, 1H), 7.21 (*m*, 1H), 7.09 (d, *J* = 16.1 Hz, 1H), 6.83 (d, *J* = 8.1 Hz, 1H), 5.78 (*s*, 2H, N-CH_2_), 3.81 (*s*, 3H, O-CH_3_) ppm. ^13^C-NMR (125 MHz, DMSO-*d*_6_) *δ*: 188.23 (C = O), 151.7, 150.7, 148.5, 146.6 (CH), 145.5 (CH), 135.5 (CH), 135.5 (CH), 134.0, 132.6 (CH), 131.5 (CH), 126.9 (CH), 126.3, 124.5 (CH), 123.5 (CH), 123.3, 116.2 (CH), 112.0 (CH), 62.5 (N-CH_2_), 56.2 (O-CH_3_) ppm. HRMS (ESI) *m/z*: calcd for C_24_H_21_BrNO_3_^+^, [M]^+^: 450.06993, 452.06788, found: 450.07152 (^79^Br), 452.06980 (^81^Br).

##### 4-(1*E,*4*E*)-5–(4-Hydroxy-3-methoxyphenyl)-3-oxopenta-1,4-dien-1-yl)-1–(4-methylbenzyl)pyridin-1-ium bromide (7i)

Reddish orange solid, Yield: 81% and decomposed at 213–214 ^0^C. ^1^H-NMR (500 MHz, DMSO-*d*_6_) *δ*: 9.79 (s, 1H, OH), 9.20 (d, *J* = 6.3 Hz, 2H, pyridine), 8.46 (d, *J* = 6.4 Hz, 2H, pyridine), 7.94 (d, *J* = 16.0 Hz, 1H, aliphatic-H), 7.86 (d, *J* = 16.0 Hz, 1H, aliphatic-H), 7.74 (d, *J* = 16.0 Hz, 1H, aliphatic-H), 7.42 (d, *J* = 7.8 Hz, 2H), 7.36 (d, *J* = 2.0 Hz, 1H), 7.21 (m, 3H), 7.09 (d, *J* = 16.0 Hz, 1H), 6.83 (d, *J* = 8.2 Hz, 1H), 5.75 (*s*, 2H, N-CH_2_), 3.81 (*s*, 3H, O-CH_3_), 2.26 (*s*, 3H, CH_3_) ppm. ^13^C-NMR (125 MHz, DMSO-*d*_6_) *δ*: 187.6 (C = O), 151.0, 150.1, 147.9, 145.9 (CH), 144.7 (CH), 138.8, 135.0 (CH), 134.8 (CH), 131.2, 129.6 (CH), 128.7 (CH), 126.2 (CH), 125.7, 123.9 (CH), 122.8 (CH), 115.6 (CH), 111.5 (CH), 62.6 (N-CH_2_), 55.6 (O-CH_3_), 20.6 (CH_3_) ppm. HRMS (ESI) *m/z*: calcd for C_25_H_24_NO_3_^+^, [M]^+^: 386.17507, found: 386.17672.

##### 4-(1*E,*4*E*)-5–(4-Hydroxy-3-methoxyphenyl)-3-oxopenta-1,4-dien-1-yl)-1–(4-nitrobenzyl)pyridin-1-ium bromide (7j)

Reddish orang solid, Yield: 79% and decomposed at 170–172 ^0^C. ^1^H-NMR (500 MHz, DMSO-*d_6_*) *δ*: 9.81 (*s*, 1H, OH), 9.22 (d, *J* = 6.3 Hz, 2H, pyridine-H), 8.50 (d, *J* = 6.3 Hz, 2H, pyridine-H), 8.26 (d, *J* = 8.3 Hz, 2H), 7.96 (d, *J* = 16.0 Hz, 1H, aliphatic-H), 7.86 (d, *J* = 16.0 Hz, 1H, aliphatic-H), 7.75 (m, 3H), 7.35 (d, *J* = 2.1 Hz, 1H), 7.21 (m, 1H), 7.09 (d, *J* = 16.1 Hz, 1H, aliphatic-H), 6.83 (d, *J* = 8.1 Hz, 1H), 5.95 (*s*, 2H, N-CH_2_), 3.81 (*s*, 3H, CH_3_) ppm. ^13^C-NMR (125 MHz, DMSO-*d*_6_) *δ*: 188.2 (C = O), 152.0, 150.7, 148.5, 148.3, 146.6 (CH), 145.8 (CH), 141.7, 135.6 (CH), 135.5 (CH), 130.4 (CH), 127.0 (CH), 126.3, 124.6 (CH), 124.6 (CH), 123.5 (CH), 116.2 (CH), 112.0 (CH), 62.2 (CH_2_), 56.2 (O-CH_3_) ppm. HRMS (ESI) *m/z*: calcd for C_24_H_21_N_2_O_5_^+^, [M]^+^: 417.14450, found: 417.14296.

##### 1-Benzyl-3-(1*E,*4*E*)-3-oxo-5-phenylpenta-1,4-dien-1-yl)pyridin-1-ium bromide (8a)

Yellow solid, Yield: 70% and mp 223–225 ^0^C. ^1^H-NMR (500 MHz, DMSO-*d*_6_) *δ*: 9.51 (*s*, 1H, pyridine-H), 8.99 (d, *J* = 6.0 Hz, 1H, pyridine-H), 8.93 (d, *J* = 8.2 Hz, 1H, pyridine-H), 8.12 (*m*, 1H), 7.91 (d, *J* = 16.1 Hz, 1H, aliphatic-H), 7.79 (d, *J* = 16.0 Hz, 1H, aliphatic-H), 7.72 (*m*, 3H), 7.55 (*m*, 2H), 7.45 (*m*, 6H), 7.19 (d, *J* = 16.1 Hz, 1H), 5.87 (*s*, 2H, N-CH_2_) ppm. ^13^C-NMR (125 MHz, DMSO-d6) *δ*: 188.4, 145.3 (CH), 144.3 (CH), 144.2 (CH), 143.5 (CH), 136.6, 134.5, 134.1 (CH), 133.0, 131.3 (CH), 130.8 (CH), 129.7 (CH), 129.3 (CH), 128.7 (CH), 128.7 (CH), 128.4 (CH), 128.3 (CH), 125.0 (CH), 64.6 (N-CH_2_) ppm. HRMS (ESI) *m/z*: calcd for C_23_H_20_NO^+^, [M]^+^: 326.15394, found: 326.15487.

##### 1–(4-Fluorobenzyl)-3-(1*E,*4*E*)-3-oxo-5-phenylpenta-1,4-dien-1-yl)pyridin-1-ium bromide (8b)

Pale yellow solid, Yield: 69% and mp 157–159 °C. ^1^H-NMR (500 MHz, DMSO-*d*_6_) *δ*: 9.89 (s, 1H, pyridine-H), 9.19 (d, *J* = 6.1 Hz, 1H, pyridine-H), 9.00 (d, *J* = 8.2 Hz, 1H, pyridine-H), 8.21 (*m*, 1H), 8.02 (d, *J* = 16.3 Hz, 1H, aliphatic-H), 7.92 (d, *J* = 16.1 Hz, 1H, aliphatic-H), 7.80 (*m*, 3H), 7.73 (m, 2H), 7.43 (*m*, 3H), 7.25 (*m*, 3H), 5.90 (*s*, 2H, N-CH_2_) ppm. ^13^C-NMR (125 MHz, DMSO-d6) *δ*: 188.7 (C = O), 163.1 (d, *J* = 246.5 Hz, C-F), 145.2 (CH), 145.02 (CH), 144.9 (CH), 144.3 (CH), 136.0, 135.4 (CH), 134.9, 132.1 (d, J = 8.6 Hz, CH), 131.4 (CH), 131.1 (CH), 130.7 (d, J = 3.2 Hz), 129.5 (CH), 129.2 (CH), 128.9 (CH), 126.2 (CH), 116.5 (d, J = 21.7 Hz, CH), 63.0 (N-CH_2_) ppm. HRMS (ESI) *m/z*: calcd for C_23_H_19_FNO^+^, [M]^+^: 344.14452, found: 344.14623.

##### 1–(4-Bromobenzyl)-3-(1*E,*4*E*)-3-oxo-5-phenylpenta-1,4-dien-1-yl)pyridin-1-ium bromide (8c)

Off-white solid, Yield: 78% and mp 203–205 ^0^C. ^1^H-NMR (500 MHz, DMSO-*d*_6_) *δ* 9.74 (*s*, 1H, pyridine-H), 9.15 (d, *J* = 6.1 Hz, 1H, pyridine-H), 8.99 (d, *J* = 8.2 Hz, 1H, pyridine-H), 8.22 (*m*, 1H), 7.95 (d, *J* = 16.1 Hz, 1H, aliphatic-H), 7.80 (*m*, 4H), 7.64 (d, *J* = 8.1 Hz, 2H), 7.55 (d, *J* = 8.1 Hz, 2H), 7.45 (*m*, 3H), 7.24 (d, *J* = 16.1 Hz, 1H, aliphatic-H), 5.85 (*s*, 2H, N-CH_2_) ppm. ^13^C-NMR (125 MHz, DMSO-d_6_) *δ*: 188.0 (C = O), 144.6 (CH), 144.5 (CH), 144.5 (CH), 143.6 (CH), 135.4, 134.8 (CH), 134.3, 133.1, 132.0 (CH), 131.2 (CH), 130.8 (CH), 130.7 (CH), 128.9 (CH), 128.6 (CH), 128.4 (CH), 125.5, 122.8, 62.6 (CH_2_) ppm. HRMS (ESI) *m/z*: calcd for C_23_H_19_BrNO^+^, [M]^+^: 404.06445, 406.06241, found: 404.06353 (^79^Br), 406.06219 (^81^Br).

##### 1–(4-Methylbenzyl)-3-(1*E,*4*E*)-3-oxo-5-phenylpenta-1,4-dien-1-yl)pyridin-1-ium bromide (8d)

Off-white solid, Yield: 73% and mp 205–206 ^0^C. ^1^H-NMR (500 MHz, DMSO-*d*_6_) *δ*: 9.82 (*s*, 1H, pyridine-H), 9.15 (d, *J* = 6.1 Hz, 1H, pyridine-H), 8.99 (d, *J* = 8.2 Hz, 1H, pyridine-H), 8.20 (*m*, 1H), 7.99 (d, *J* = 16.2 Hz, 1H, aliphatic-H), 7.88 (d, *J* = 16.1 Hz, 1H, aliphatic-H), 7.80 (*m*, 3H), 7.50 (*m*, 2H), 7.44 (*m*, 3H), 7.23 (*m*, 3H), 5.83 (*s*, 2H, N-CH2), 2.26 (*s*, 3H, CH3) ppm. ^13^C-NMR (125 MHz, DMSO-*d*_6_) *δ*: 188.7 (C = O), 145.1, 145.0 (CH), 144.9 (CH), 144.1 (CH), 139.5, 135.9, 135.4 (CH), 134.9, 131.5, 131.4 (CH), 131.2 (CH),130.2 (CH), 129.5 (CH), 129.5 (CH), 129.1 (CH), 128.9 (CH), 126.2 (CH), 63.8 (CH_2_), 21.2 (CH_3_) ppm. HRMS (ESI) *m/z*: calcd for C_24_H_22_NO^+^, [M]^+^: 340.16959, found: 340.17090.

##### 1–(4-Nitrobenzyl)-3-(1*E,*4*E*)-3-oxo-5-phenylpenta-1,4-dien-1-yl)pyridin-1-ium bromide (8e)

Off-white solid, Yield: 76% and mp 145–147 ^0^C. ^1^H-NMR (500 MHz, DMSO-*d*_6_) *δ*: 9.85 (*s*, 1H, pyridine-H), 9.21 (d, *J* = 6.1 Hz, 1H, pyridine-H), 9.03 (d, *J* = 8.1 Hz, 1H, pyridine-H), 8.26 (*m*, 3H), 7.99 (d, *J* = 16.3 Hz, 1H, aliphatic-H), 7.83 (*m*, 6H), 7.44 (*m*, 3H), 7.23 (d, *J* = 16.3 Hz, 1H, aliphatic-H), 6.06 (*s*, 2H, N-CH_2_) ppm. ^13^C-NMR (125 MHz, DMSO-*d*_6_) *δ*: 188.1 (C = O), 147.8, 144.9 (CH), 144.8 (CH), 144.6 (CH), 144.0 (CH), 140.7, 135.5, 134.8 (CH), 134.3, 130.8 (CH), 130.6 (CH), 130.2 (CH), 128.9 (CH), 128.6 (CH), 128.4 (CH), 125.6 (CH), 124.0 (CH), 62.2 (N-CH_2_) ppm. HRMS (ESI) *m/z*: calcd for C_23_H_19_N_2_O_3_^+^, [M]^+^: 371.13902, found: 371.14040.

##### 1-Benzyl-3-(1*E,*4*E*)-5–(4-hydroxy-3-methoxyphenyl)-3-oxopenta-1,4-dien-1-yl)pyridin-1-ium bromide (8f)

Yellow solid, Yield: 91% and mp 196–198 ^0^C. ^1^H-NMR (500 MHz, DMSO-*d*_6_) *δ*: 9.87 (*s*, 1H, pyridine-H), 9.75 (*s*, 1H, OH), 9.17 (d, *J* = 5.8 Hz, 1H, pyridine-H), 9.00 (d, *J* = 6.6 Hz, 1H, pyridine-H), 8.21 (*m*, 1H), 7.91 (*m*, 2H), 7.76 (dd, *J* = 16.1 Hz, 1H, aliphatic-H), 7.60 (*m*, 2H), 7.41 (m, 4H), 7.24 (*m*, 1H), 7.07 (dd, *J* = 16.2 Hz, 1H, aliphatic-H), 6.83 (*m*, 1H), 5.90 (d, *J* = 5.2 Hz, 2H, N-CH_2_), 3.82 (d, *J* = 5.3 Hz, 3H, O-CH_3_) ppm. ^13^C-NMR (125 MHz, DMSO-*d*_6_) *δ*: 188.2 (C = O), 150.5, 148.5, 146.1 (CH), 144.9 (CH), 144.1 (CH), 136.2, 134.5 (CH), 134.5, 131.4 (CH), 129.9 (CH), 129.6 (CH), 129.4(CH), 128.9 (CH), 126.4, 124.4 (CH), 123.4 (CH), 116.2 (CH), 112.1 (CH), 63.9 (CH), 56.2 (CH_2_) ppm. HRMS (ESI) *m/z*: calcd for C_24_H_22_NO_3_^+^, [M]^+^: 372.15942, found: 372.16107.

##### 1–(4-Fluorobenzyl)-3-(1*E*,4E)-5–(4-hydroxy-3-methoxyphenyl)-3-oxopenta-1,4-dien-1-yl)pyridin-1-ium bromide (8 g)

Yellow solid, Yield: 90% and mp 214–216 ^0^C. ^1^H-NMR (500 MHz, DMSO-*d*_6_) *δ*: 9.85 (*s*, 1H, pyridine-H), 9.75 (*s*, 1H, OH), 9.16 (d, *J* = 6.1 Hz, 1H, pyridine-H), 8.99 (d, *J* = 8.2 Hz, 1H, pyridine-H), 8.20 (*m*, 1H), 7.90 (dd, *J* = 16.0 Hz, 2H, aliphatic-H), 7.72 (*m*, 3H), 7.39 (d, *J* = 2.0 Hz, 1H), 7.25 (*m*, 3H), 7.06 (d, *J* = 16.1 Hz, 1H, aliphatic-H), 6.82 (d, *J* = 8.1 Hz, 1H), 5.88 (*s*, 2H, N-CH_2_), 3.81 (*s*, 3H, O-CH_3_) ppm. ^13^C-NMR (125 MHz, DMSO-*d*_6_) *δ*: 188.2 (C = O), 163.1 (d, *J* = 246.7 Hz, C-F), 150.5, 148.5, 146.1 (CH), 144.8 (CH), 144.1 (CH), 136.1, 134.5 (CH), 132.1 (d, *J* = 8.6 Hz, CH), 131.4 (CH), 130.7 (d, *J* = 3.1 Hz), 128.9 (CH), 126.4, 124.4 (CH), 123.4 (CH), 116.5 (d, *J* = 21.9 Hz, CH), 116.2 (CH), 112.1 (CH), 63.0 (CH_2_), 56.2 (O-CH_3_) ppm. HRMS (ESI) *m/z*: calcd for C_24_H_21_FNO_3_^+^, [M]^+^: 390.15000, found: 390.15171.

##### 1–(4-Bromobenzyl)-3-(1*E,*4*E*)-5–(4-hydroxy-3-methoxyphenyl)-3-oxopenta-1,4-dien-1-yl)pyridin-1-ium bromide (8h)

Yellow solid, Yield: 91% and mp 226–228 ^0^C. ^1^H-NMR (500 MHz, DMSO-*d*_6_) δ: 9.79 (*s*, 1H, pyridine-H), 9.75 (*s*, 1H, OH), 9.14 (d, *J* = 6.1 Hz, 1H, pyridine-H), 8.99 (d, *J* = 8.1 Hz, 1H, pyridine-H), 8.21 (*m*, 1H), 7.88 (dd, *J* = 16.1 Hz, 2H, aliphatic-H), 7.74 (d, *J* = 16.1 Hz, 1H, aliphatic-H), 7.63 (d, *J* = 8.2 Hz, 2H), 7.56 (d, *J* = 8.3 Hz, 2H), 7.38 (d, *J* = 1.9 Hz, 1H), 7.22 (*m*, 1H), 7.06 (d, *J* = 16.2 Hz, 1H, aliphatic-H), 6.82 (d, *J* = 8.1 Hz, 1H), 5.86 (*s*, 2H, N-CH_2_), 3.81 (*s*, 3H, O-CH_3_) ppm. ^13^C-NMR (125 MHz, DMSO-*d*_6_) *δ*: 188.2 (C = O), 150.5, 148.5, 146.1 (CH), 145.0 (CH), 144.2 (CH), 136.2, 134.5 (CH), 133.7, 132.5 (CH), 131.7 (CH), 131.4 (CH), 128.9 (CH), 126.4, 124.4 (CH), 123.4, 123.4 (CH), 116.2, 112.0, 63.1 (N-CH_2_), 56.2 (O-CH_3_) ppm. HRMS (ESI) *m/z*: calcd for C_24_H_21_BrNO_3_^+^, [M]^+^: 450.06993, 452.06788, found: 450.07190 (^79^Br), 452.07010 (^81^Br).

##### 3-(1*E,*4*E*)-5–(4-Hydroxy-3-methoxyphenyl)-3-oxopenta-1,4-dien-1-yl)-1–(4-methylbenzyl)pyridin-1-ium bromide (8i)

Yellow solid, Yield: 90% and mp 200–203 ^0^C. ^1^H- NMR (500 MHz, DMSO-*d*_6_) *δ*: 9.79 (*s*, 1H, pyridine-H), 9.75 (*s*, 1H, OH), 9.13 (d, *J* = 6.1 Hz, 1H, pyridine-H), 8.98 (d, *J* = 8.1 Hz, 1H, pyridine-H), 8.19 (*m*, 1H), 7.87 (dd, *J* = 16.0 Hz, 2H, aliphatic-H), 7.75 (d, *J* = 16.0 Hz, 1H, aliphatic-H), 7.49 (d, *J* = 7.8 Hz, 2H), 7.38 (*s*, 1H), 7.22 (d, *J* = 7.7 Hz, 3H), 7.06 (d, *J* = 16.0 Hz, 1H, aliphatic-H), 6.82 (d, *J* = 8.1 Hz, 1H), 5.82 (*s*, 2H, N-CH2), 3.81 (*s*, 3H, O-CH3), 2.26 (*s*, 3H, CH_3_) ppm. ^13^C-NMR (125 MHz, DMSO-*d*_6_) *δ*: 188.2 (C = O), 150.5, 148.5, 146.0 (CH), 144.8 (CH), 144.0 (CH), 139.5, 136.1, 134.6 (CH), 131.5, 131.4 (CH), 130.2 (CH), 129.4 (CH), 128.9 (CH), 126.4, 124.4 (CH), 123.4 (CH), 116.2 (CH), 112.0 (CH), 63.8 (N-CH2), 56.2 (O-CH_3_), 21.2 (CH_3_) ppm. HRMS (ESI) *m/z*: calcd for C_25_H_24_NO_3_^+^, [M]^+^: 386.17507, found: 386.17650.

##### 3-(1*E,*4*E*)-5–(4-Hydroxy-3-methoxyphenyl)-3-oxopenta-1,4-dien-1-yl)-1–(4-nitrobenzyl)pyridin-1-ium bromide (8j)

Yellow solid, Yield: 93% and mp 190–192 ^0^C. ^1^H- NMR (500 MHz, DMSO-*d*_6_) *δ*: 9.84 (*s*, 1H, pyridine), 9.75 (*s*, 1H, OH), 9.19 (d, *J* = 6.1 Hz, 1H, pyridine), 9.03 (d, *J* = 8.2 Hz, 1H, pyridine), 8.25 (*m*, 3H), 7.87 (*m*, 4H), 7.75 (d, *J* = 16.0 Hz, 1H, aliphatic-H), 7.38 (*s*, 1H), 7.23 (d, *J* = 8.2 Hz, 1H), 7.06 (d, *J* = 16.1 Hz, 1H, aliphatic-H), 6.82 (d, *J* = 8.1 Hz, 1H), 6.06 (*s*, 2H, N-CH2), 3.81 (*s*, 3H, O-CH3) ppm. ^13^C-NMR (125 MHz, DMSO-*d*_6_) *δ*: 188.2 (C = O), 150.5, 148.5, 148.4, 146.1 (CH), 145.3 (CH), 144.4 (CH), 141.3, 136.3, 134.5 (CH), 131.4 (CH), 130.7 (CH), 129.0 (CH), 126.4, 124.5 (CH), 124.4 (CH), 123.4 (CH), 116.2 (CH), 112.0 (CH), 62.8 (CH_2_), 56.2 (O-CH_3_) ppm. HRMS (ESI) *m/z*: calcd for C_24_H_21_N_2_O_5_^+^, [M]^+^: 417.14450, found: 417.14644.

## Biology

### Acetyl- and butyrylcholinesterase inhibition assays (IC_50_)

The AChE and BChE inhibitory activity assays were performed using a BioTek Epoch 2 microplate spectrophotometer in clear, flat-bottom 96-well plates. Compounds **7a-j** and **8a-j** were dissolved in 1:1–1:1.5 water/methanol, depending on their solubility. To each well, 295 µL phosphate buffer pH 8.0, 10 µL enzyme solution (AChE/BChE), and 10 µL target compound solutions were added together and allowed to stand for 4 min at room temperature. Then, 10 µL of 0.01 M solution freshly prepared Ellman’s reagent 5,5′-dithiobis(2-nitrobenzoic acid) (DTNB) was added to each well. The reaction in the wells was started by the addition of 2 µL of 0.075 M solution freshly prepared substrate (ATCh (acetylthiocholine iodide)/BTCh (S-butyrylthiocholine iodide)) solution. The solution was mixed immediately and incubated for 2:30 min for AChE or for 5 min for BChE inside the microplate reader at 26 °C. The absorbance was measured at 412 nm, and the assay was conducted in triplicate; each concentration was in duplicate on each plate^20^. The IC_50_ values were calculated graphically by nonlinear regression analysis of inhibition curves that were obtained by plotting the logarithm of the inhibitor concentrations versus the percentage of inhibition (log(inhibitor) vs. normalised response) using GraphPad Prism 8 software (See Supplementary Information for details).

### Enzyme inhibition constant (K_i_) determination

The rates of hydrolysis of the ATCh mean velocity (V) for the most potent compounds were measured spectrophotometrically using the kinetic assay protocol available in Gen5 software. The changes in absorbance in each well at 405 nm and 26 °C were measured using the microplate reader at 48-s intervals over 10 min. The assay was performed in triplicate using different inhibitor concentrations. Compounds **7a-j** and **8a-j** concentrations were prepared according to the IC_50_ value of each inhibitor using [IC_50_/2], [IC_50_], and [2 × IC_50_] as the final concentrations. The assay was carried out in clear, flat-bottom 96-well plates, to which 5 µL distilled water was added to all wells of columns 2–3, rows B-F. Then, 5 µL of the lowest concentration of inhibitor solution was added to the wells of columns 4–5, 5 µL of the median concentration of inhibitor solution was added to the wells of columns 6–7, and 5 µL of the highest concentration of inhibitor solution was added to the wells of columns 8–9. Next, 275 μL enzyme solution was added to the wells, and the plates were incubated for 4 min. Then, 10 μL 2.5 mM DTNB was added to the wells, followed by 10 μL of 32, 16, 8, 4, or 2 mM ATCh solutions to initiate the reactions. The plates were placed in a microplate reader for direct kinetic measurements.

The inhibition constant (*K_i_)* for each inhibitor was calculated by averaging the ATCh hydrolysis rates from three independent experiments. Using GraphPad Prism 8 software, nonlinear regression was used to calculate the *K_m_* and *V_max_* values of the Michaelis-Menten kinetics by plotting [substrate] versus velocity. For the Lineweaver-Burk plots, linear regression was used to plot [1/ATCh] versus [substrate]. Finally, the *K_i_* value*s* were obtained by plotting the slopes against [inhibitor] ^20^.

### Antioxidant activity assay (ABTS)

The antioxidant activity was determined using a radical cation (ABTS**^.+^**) decolorisation assay, as described by Xiao et al.^21^ with a few modifications. The assay used a BioTek Epoch 2 microplate spectrophotometer (Agilent, USA) with clear, flat-bottom 96-well plates. The 7 mmol/L ABTS**^.+^** stock solution was prepared by dissolving 96.02 mg ABTS in acetic acid buffer (pH 4.5) and to a final volume of 25 ml, which was kept in the dark at 0–4 °C. A 2.45 mmol/L potassium persulfate (K_2_S_2_O_8_) stock solution was prepared by dissolving 66.24 mg K_2_S_2_O_8_ in acetic acid buffer solution with pH 4.5, and then diluted to 100 ml and kept in the dark at 0–4 °C. The ABTS working solution was prepared by mixing similar quantities of 7 mmol/L ABTS and 2.45 mmol/L K_2_S_2_O_8_. This solution was stored in a dark cabinet for 16 h before use, when it was diluted with distilled water to obtain an absorbance of 0.70 at 734 nm. In a clear, flat-bottom 96-well plate, 200 μL of ABTS working solution and 10.0 μL sample solution at different concentrations (0.0312–0.5 mg/mL) were mixed well and incubated in the dark for 7 min before the absorbance was measured at 734 nm using a microplate reader. A blank was run for each assay, and all measurements were performed after 7 min. The ABTS scavenging capacity of inhibitors was compared with that of trolox and α-tocopherol. The percentage of inhibition was calculated as follows:

$$\mathrm{Inhibition}\%=(1-\frac{A_{s}}{Ac})\times 100$$

where A_C_ is the absorbance of the blank, and A_S_ is the absorbance in the presence of the inhibitor.

### Molecular docking study

The crystallographic structure of AChE complexed with donepezil (PDB entry: 1EVE) was obtained from the RCSB Protein Data Bank. Chain A of the crystal structure was used with a grid box of 25 × 25 × 25 Å, appropriate for covering the interaction region between the ligand and enzyme. The ligand flexible docking study was performed using the docking program AutoDock Vina (1.5.6) ^22^. The exhaustiveness parameter was set to 100, whereas the default parameters were used in all other cases. The docked structures were visualised using the UCSF Chimaera program (1.14)^23^.

## Conclusion

To ensure a good quality of life and health for people, we aimed to develop anti-AD agents. Herein, we report synthesising a series of potent AChE inhibitors bearing an *N*-benzylpyridinium moiety. *In vitro* assays indicated the potency of the prepared compounds, especially those containing a vanillin moiety, in additon to their antioxidant activity. Eight of the prepared compounds exhibited AChE inhibitory activity in the nanomolar range. Docking studies explained the potency and binding of these compounds to the active site of AChE. Overall, the results of this study confirmed the promising potential of the synthesised compounds as AD inhibitors. However, further *in vivo* studies are required to confirm this hypothesis.

## Supplementary Material

Supplemental Material

## References

[1] 2023 Alzheimer’s disease facts and figures. Alzheimers Dement. 2023;19(4):1598–1695. 36918389 10.1002/alz.13016

[2] Rahman A, Hossen MA, Chowdhury MFI, Bari S, Tamanna N, Sultana SS, Haque SN, Al Masud A, Saif-Ur-Rahman KM. Aducanumab for the treatment of Alzheimer’s disease: a systematic review. Psychogeriatrics. 2023;23(3):512–522. 36775284 10.1111/psyg.12944 PMC11578022

[3] van Dyck CH, Swanson CJ, Aisen P, Bateman RJ, Chen C, Gee M, Kanekiyo M, Li D, Reyderman L, Cohen S, et al. Lecanemab in early Alzheimer’s disease. N Engl J Med. 2023;388(1):9–21. 36449413 10.1056/NEJMoa2212948

[4] Chen ZR, Huang JB, Yang SL, Hong FF. Role of cholinergic signaling in Alzheimer’s disease. Molecules. 2022;27(6):1816. doi:10.3390/molecules27061816. PMC8949236 35335180

[5] Inestrosa NC, Alvarez A, Pérez CA, Moreno RD, Vicente M, Linker C, Casanueva OI, Soto C, Garrido J. Acetylcholinesterase accelerates assembly of amyloid-beta-peptides into alzheimer’s fibrils: Possible role of the peripheral site of the enzyme. Neuron. 1996;16(4):881–891. 8608006 10.1016/s0896-6273(00)80108-7

[6] Carvajal FJ, Inestrosa NC. Interactions of ache with aβ aggregates in Alzheimer’s brain: Therapeutic relevance of idn 5706. Front Mol Neurosci. 2011;4(:19. 21949501 10.3389/fnmol.2011.00019 PMC3172730

[7] Kareem RT, Abedinifar F, Mahmood EA, Ebadi AG, Rajabi F, Vessally E. The recent development of donepezil structure-based hybrids as potential multifunctional anti-Alzheimer’s agents: highlights from 2010 to 2020. RSC Adv. 2021;11(49):30781–30797. 35498922 10.1039/d1ra03718h PMC9041380

[8] Eissa KI, Kamel MM, Mohamed LW, Kassab AE. Development of new Alzheimer’s disease drug candidates using donepezil as a key model. Arch Pharm. 2023;356(1):2200398. 10.1002/ardp.202200398 36149034

[9] Mostofi M, Mohammadi Ziarani G, Mahdavi M, Moradi A, Nadri H, Emami S, Alinezhad H, Foroumadi A, Shafiee A. Synthesis and structure-activity relationship study of benzofuran-based chalconoids bearing benzylpyridinium moiety as potent acetylcholinesterase inhibitors. Eur J Med Chem. 2015;103(:361–369. 26363872 10.1016/j.ejmech.2015.08.061

[10] Wang C, Wu Z, Cai H, Xu S, Liu J, Jiang J, Yao H, Wu X, Xu J. Design, synthesis, biological evaluation and docking study of 4-isochromanone hybrids bearing n-benzyl pyridinium moiety as dual binding site acetylcholinesterase inhibitors. Bioorg Med Chem Lett. 2015;25(22):5212–5216. 26454504 10.1016/j.bmcl.2015.09.063

[11] Jabir NR, Khan FR, Tabrez S. Cholinesterase targeting by polyphenols: a therapeutic approach for the treatment of Alzheimer’s disease. CNS Neurosci Ther. 2018;24(9):753–762. 29770579 10.1111/cns.12971 PMC6489761

[12] Yang F, Lim GP, Begum AN, Ubeda OJ, Simmons MR, Ambegaokar SS, Chen PP, Kayed R, Glabe CG, Frautschy SA, et al. Curcumin inhibits formation of amyloid beta oligomers and fibrils, binds plaques, and reduces amyloid in vivo. J Biol Chem. 2005;280(7):5892–5901. 15590663 10.1074/jbc.M404751200

[13] Ramalakshmi N, R SR, C NN. Multitarget directed ligand approaches for Alzheimer’s disease: a comprehensive review. Mini Rev Med Chem. 2021;21(16):2361–2388. 33820504 10.2174/1389557521666210405161205

[14] Maramai S, Benchekroun M, Gabr MT, Yahiaoui S. Multitarget therapeutic strategies for Alzheimer’s disease: review on emerging target combinations. Biomed Res Int. 2020;2020(:5120230–27. 32714977 10.1155/2020/5120230 PMC7354643

[15] Yücel YY, Tacal O, Ozer I. Comparative effects of cationic triarylmethane, phenoxazine and phenothiazine dyes on horse serum butyrylcholinesterase. Arch Biochem Biophys. 2008;478(2):201–205. 18656440 10.1016/j.abb.2008.07.006

[16] Ganeshpurkar A, Singh R, Shivhare S, Kumar D, Gutti G, Singh R, Kumar A, Singh , SK. Divya Improved machine learning scoring functions for identification of electrophorus electricus’s acetylcholinesterase inhibitors. Mol Divers. 2022;26(3):1455–1479. 34328603 10.1007/s11030-021-10280-w

[17] Sugimoto H, Ogura H, Arai Y, Limura Y, Yamanishi Y. Research and development of donepezil hydrochloride, a new type of acetylcholinesterase inhibitor. Jpn J Pharmacol. 2002;89(1):7–20. 12083745 10.1254/jjp.89.7

[18] Ahmed M, Rocha JBT, Corrêa M, Mazzanti CM, Zanin RF, Morsch ALB, Morsch VM, Schetinger MRC. Inhibition of two different cholinesterases by tacrine. Chem Biol Interact. 2006;162(2):165–171. 16860785 10.1016/j.cbi.2006.06.002

[19] Liston DR, Nielsen JA, Villalobos A, Chapin D, Jones SB, Hubbard ST, Shalaby IA, Ramirez A, Nason D, White WF. Pharmacology of selective acetylcholinesterase inhibitors: implications for use in alzheimer’s disease. Eur J Pharmacol. 2004;486(1):9–17. 14751402 10.1016/j.ejphar.2003.11.080

[20] Darras FH, Pang Y-P. On the use of the experimentally determined enzyme inhibition constant as a measure of absolute binding affinity. Biochem Biophys Res Commun. 2017;489(4):451–454. 28571743 10.1016/j.bbrc.2017.05.168

[21] Xiao F, Xu T, Lu B, Liu R. Guidelines for antioxidant assays for food components. Food Frontiers. 2020;1(1):60–69.

[22] Trott O, Olson AJ. Autodock vina: Improving the speed and accuracy of docking with a new scoring function, efficient optimization, and multithreading. J Comput Chem. 2010;31(2):455–461. 19499576 10.1002/jcc.21334 PMC3041641

[23] Pettersen EF, Goddard TD, Huang CC, Couch GS, Greenblatt DM, Meng EC, Ferrin TE. Ucsf chimera—a visualization system for exploratory research and analysis. J Comput Chem. 2004;25(13):1605–1612. 15264254 10.1002/jcc.20084

