# Supplementary material for: Synthesis, biological evaluation, and computational studies of N-benzyl pyridinium–curcumin derivatives as potent AChE inhibitors with antioxidant activity
Source: J Enzyme Inhib Med Chem. 2023 Nov 20;38(1):2281264. doi: 10.1080/14756366.2023.2281264 (PMC11003481; doi:10.1080/14756366.2023.2281264)
Supplement: Supplemental Material [file IENZ_A_2281264_SM5563.pdf]

Synthesis, Biological Evaluation, and Computational Studies of *N*-benzyl Pyridinium-Curcumin Derivatives as Potent AChE Inhibitors with Antioxidant Activity

Nafisah Al-Rifai[1]*, Nemeh Al-khalaileh[2], Jalal Zahra[2], Musa El-barghouthi[3], Fouad Darras[4]

Supporting Information

1. NMR Spectra--2

2. Inhibition assay graphs--26

1. NMR Spectra

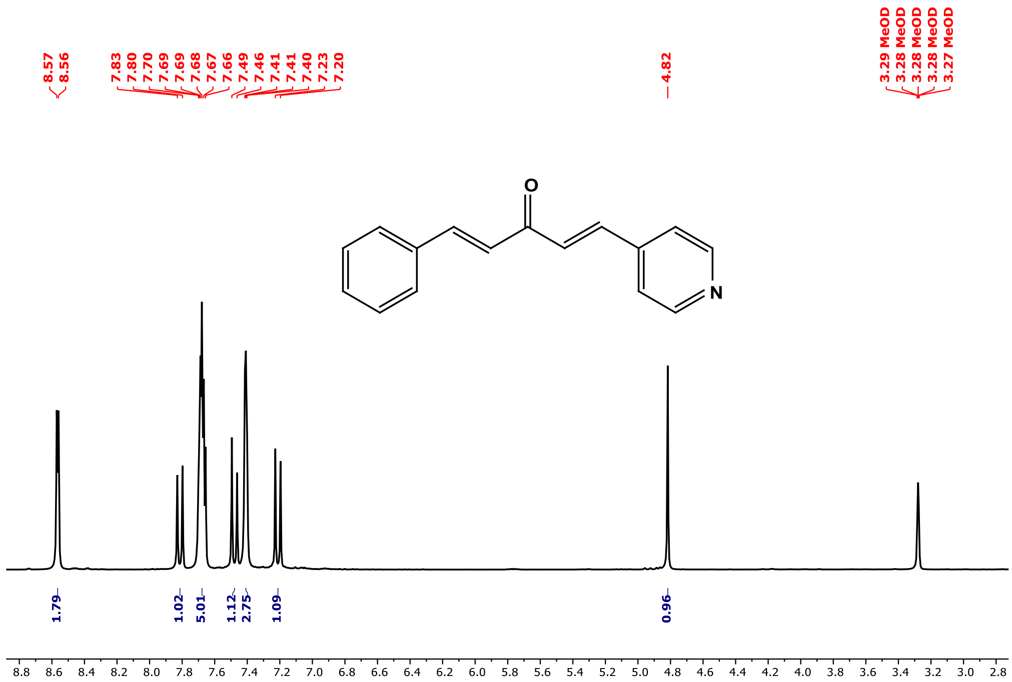

Figure S1: ^{1}H-NMR spectrum of compound **12a** (CD_3OD).

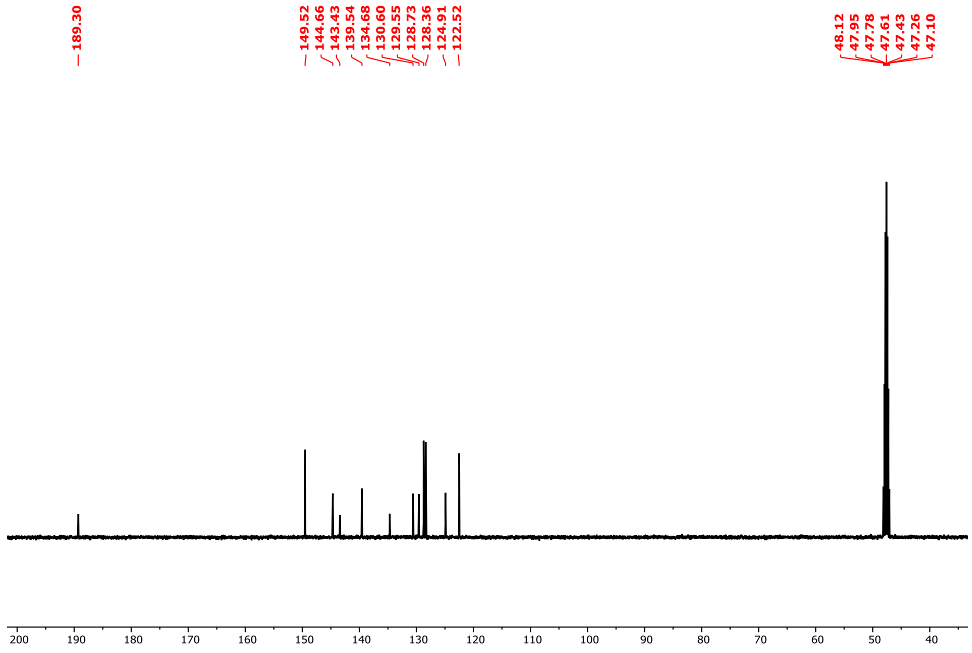

Figure S2: ^{13}C-NMR spectrum of compound **12a** (CD_3OD).

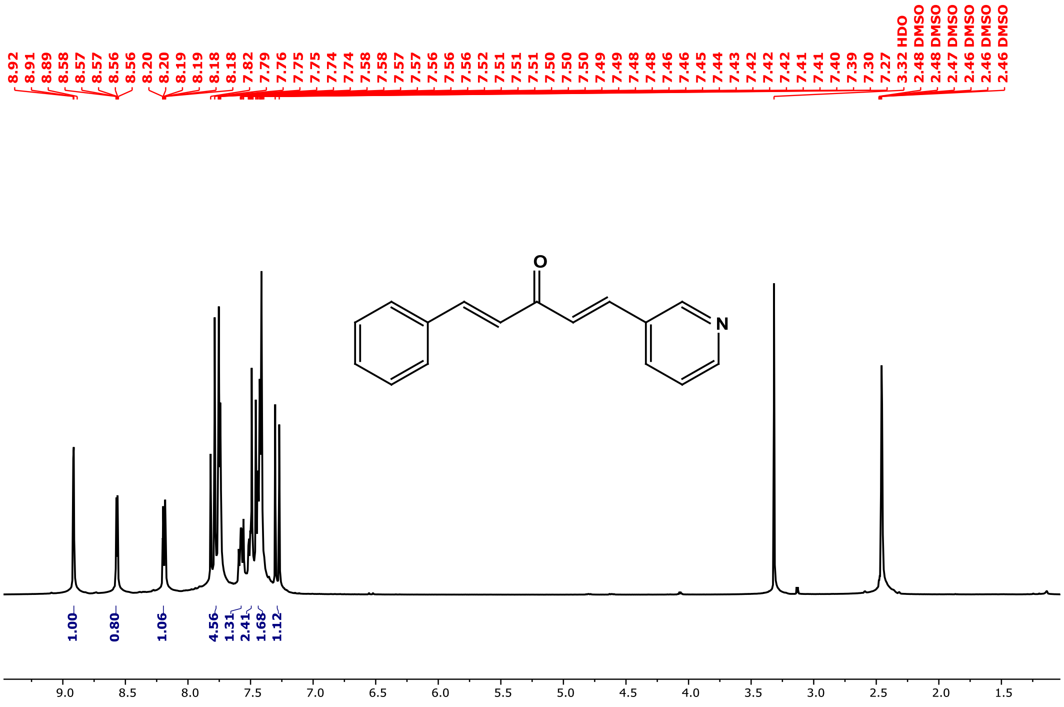

Figure S3: ^{1}H-NMR spectrum of compound **12b** (DMSO-d_6).

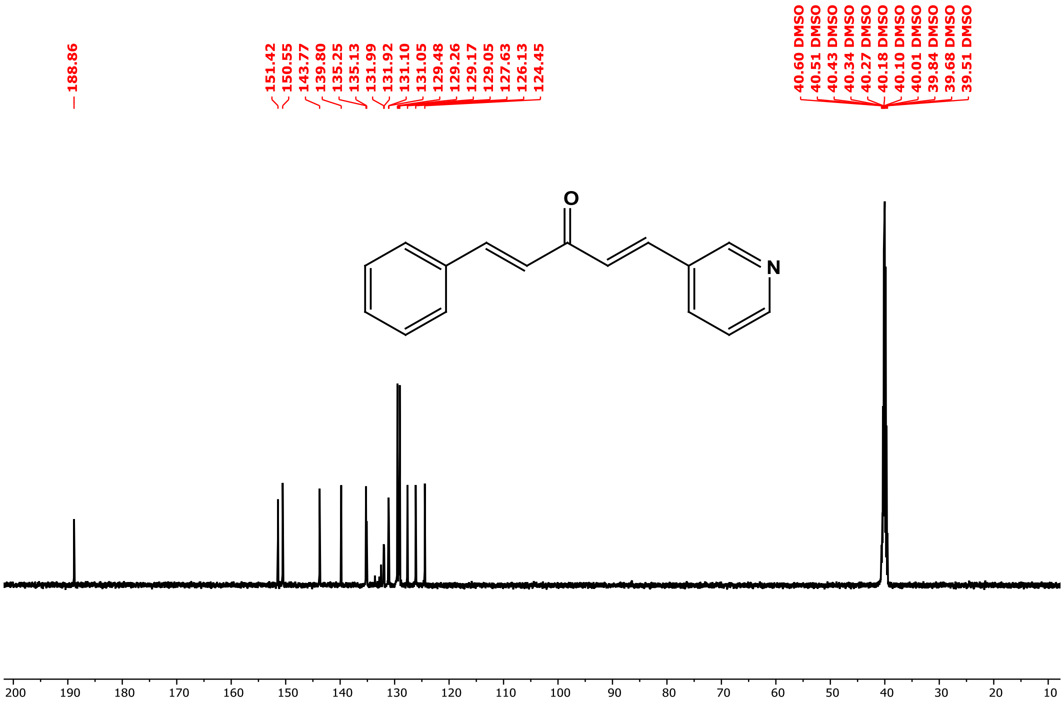

Figure S4: ^{13}C-NMR spectrum of compound **12b** (DMSO-d_6).

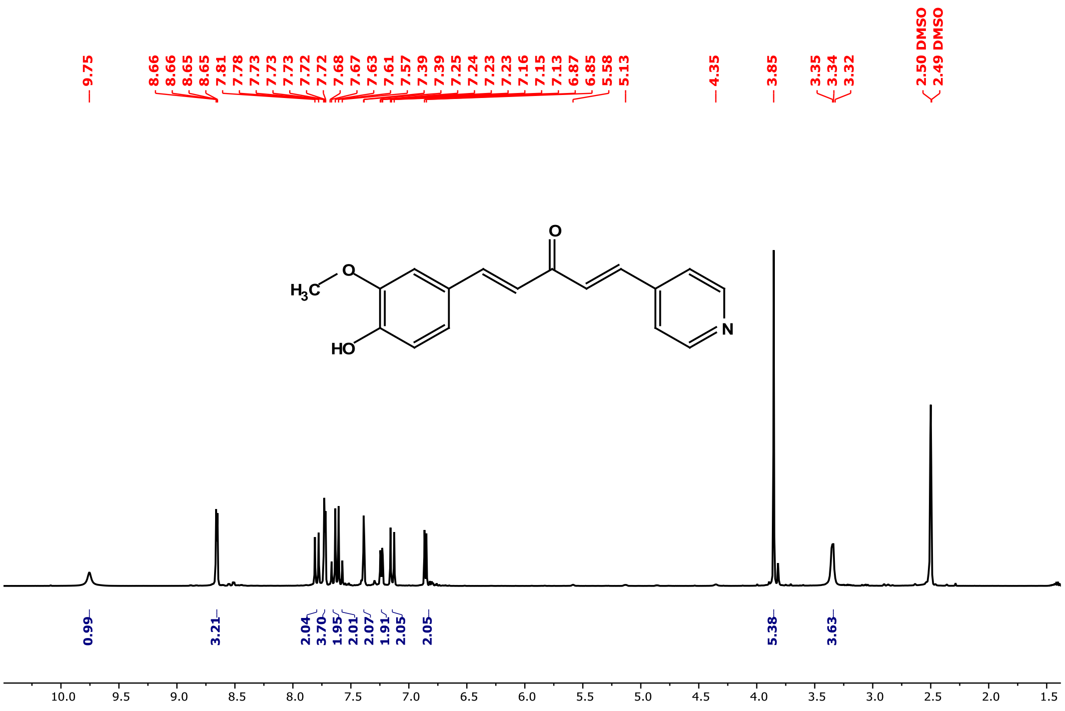

Figure S5: ^{1}H-NMR spectrum of compound **13a** (DMSO-d_6).

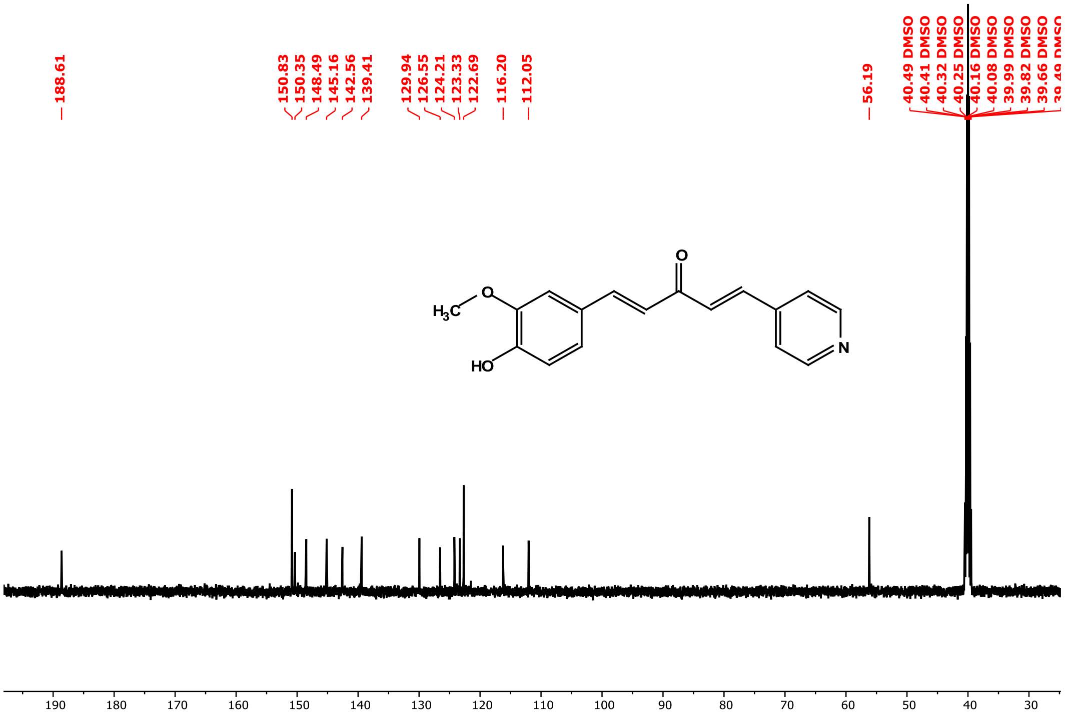

Figure S6: ^{13}C-NMR spectrum of compound **13a** (DMSO-d_6).

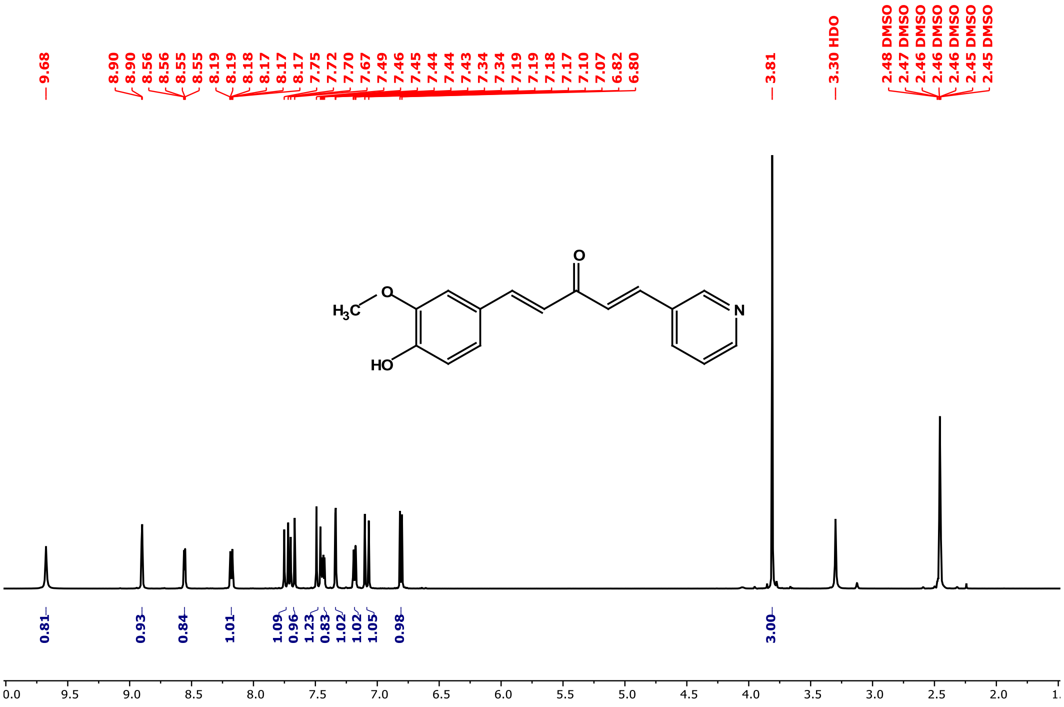

Figure S7: ^{1}H-NMR spectrum of compound **13b** (DMSO-d_6).

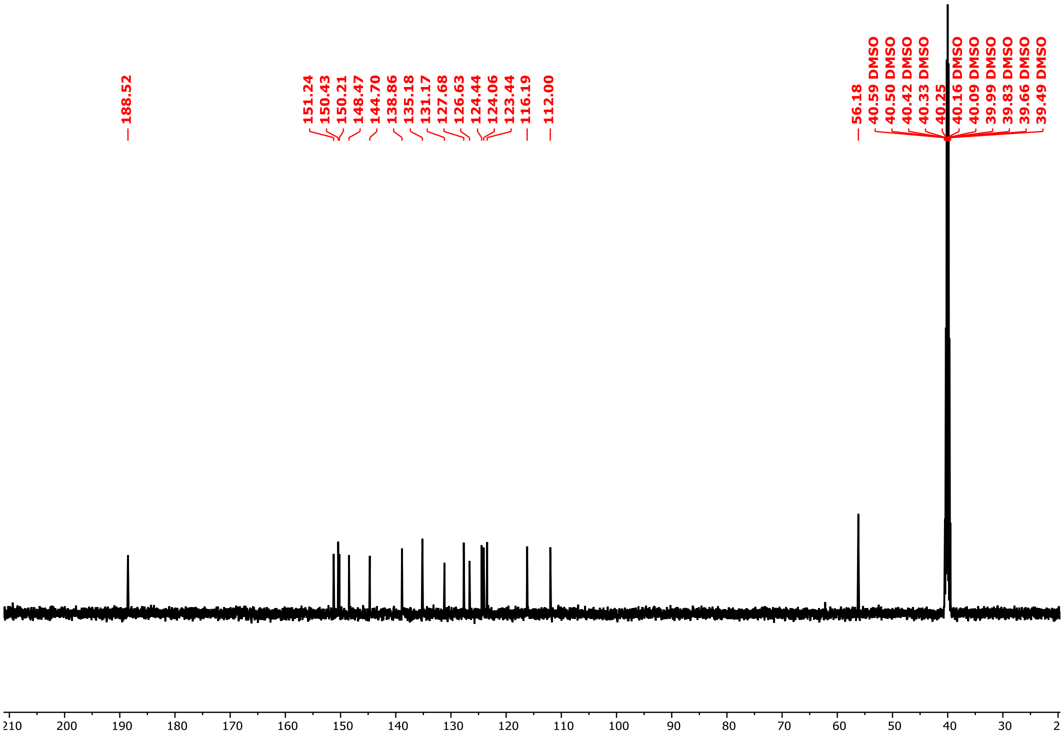

Figure S8: ^{13}C-NMR spectrum of compound **13b** (DMSO-d_6).

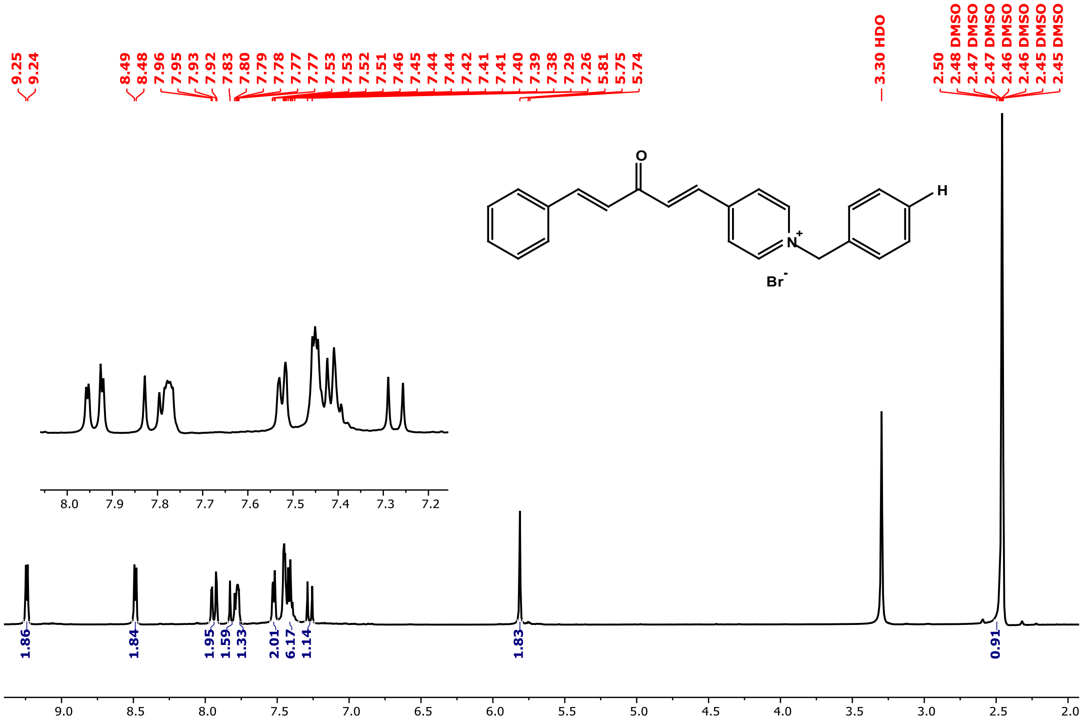

Figure S9: ^{1}H-NMR spectrum of compound **7a** (DMSO-d_6).

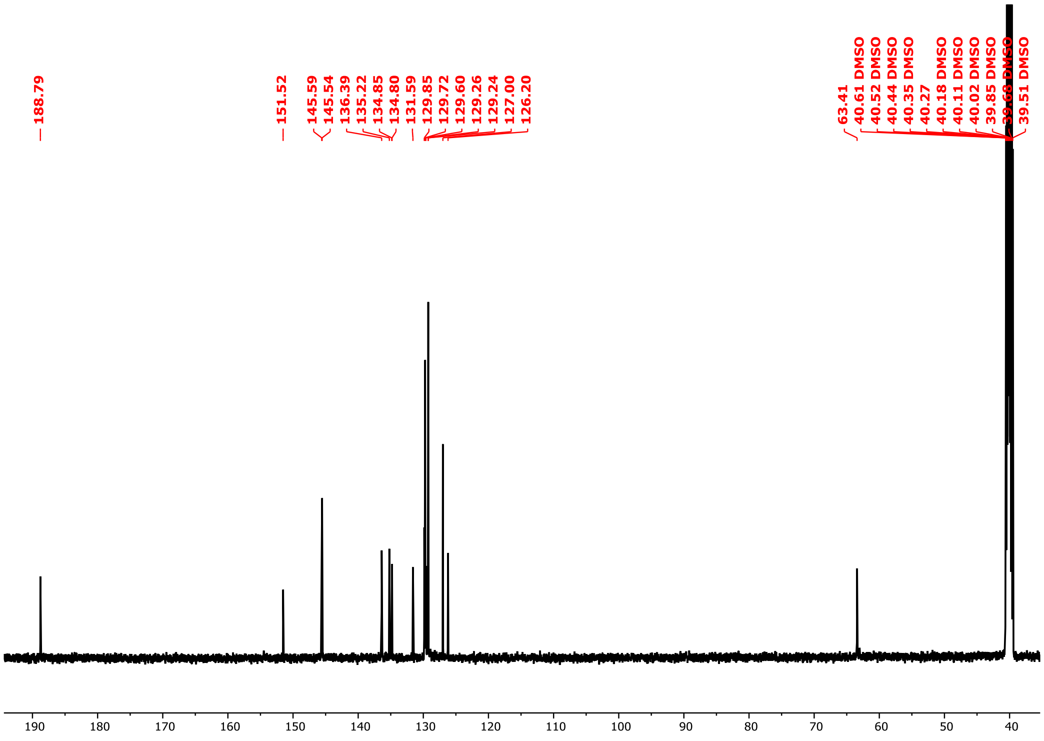

Figure S10: ^{13}C-NMR spectrum of compound **7a** (DMSO-d_6).

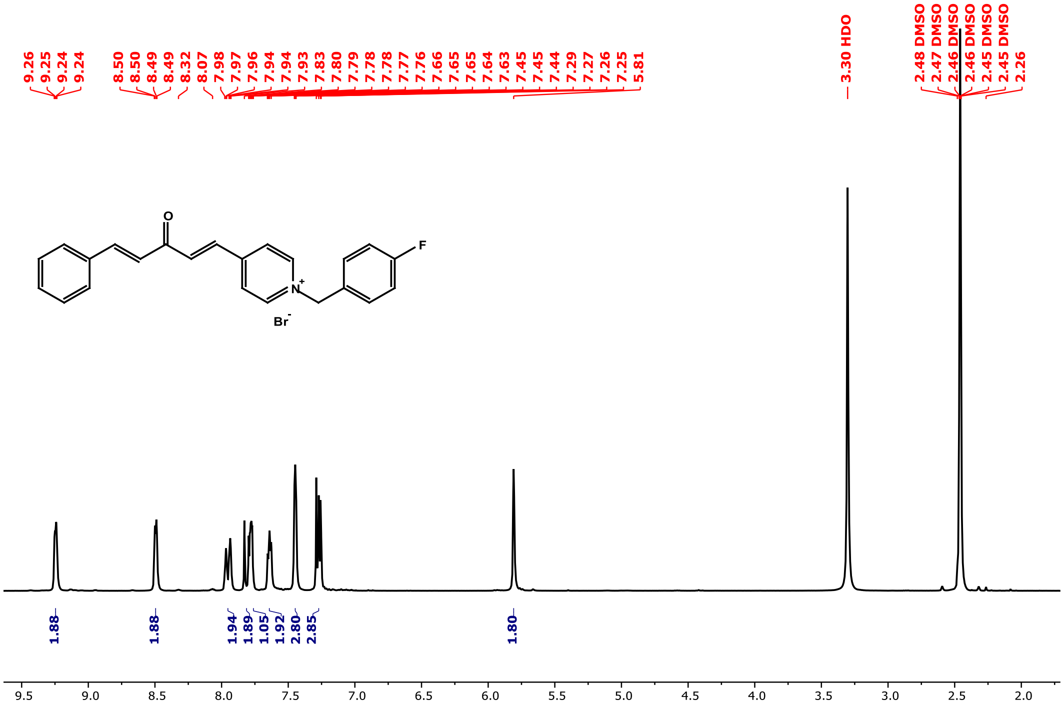

Figure S11: ^{1}H-NMR spectrum of compound **7b** (DMSO-d_6).

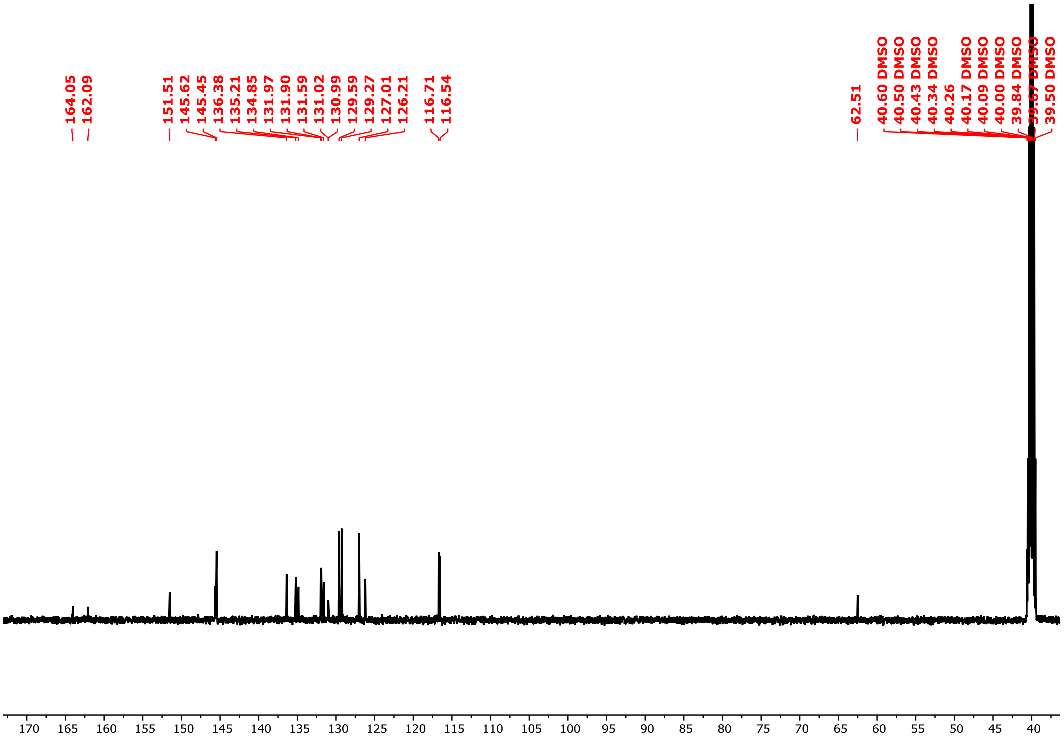

Figure S12: ^{13}C-NMR spectrum of compound **7b** (DMSO-d_6).

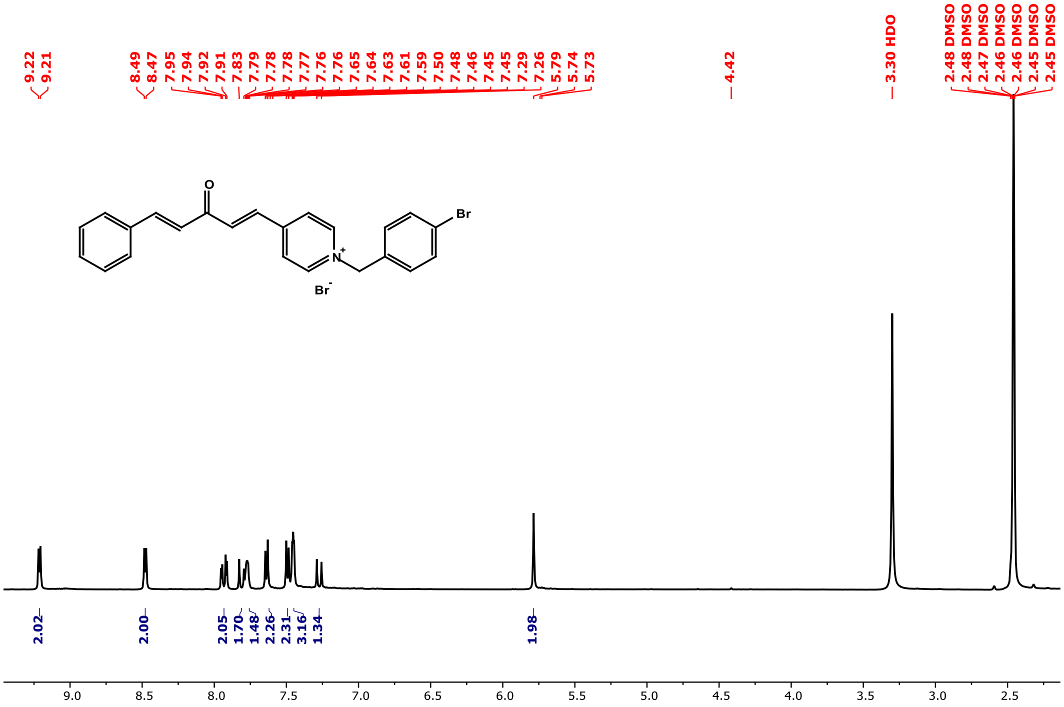

Figure S13: ^{1}H-NMR spectrum of compound **7c** (DMSO-d_6).

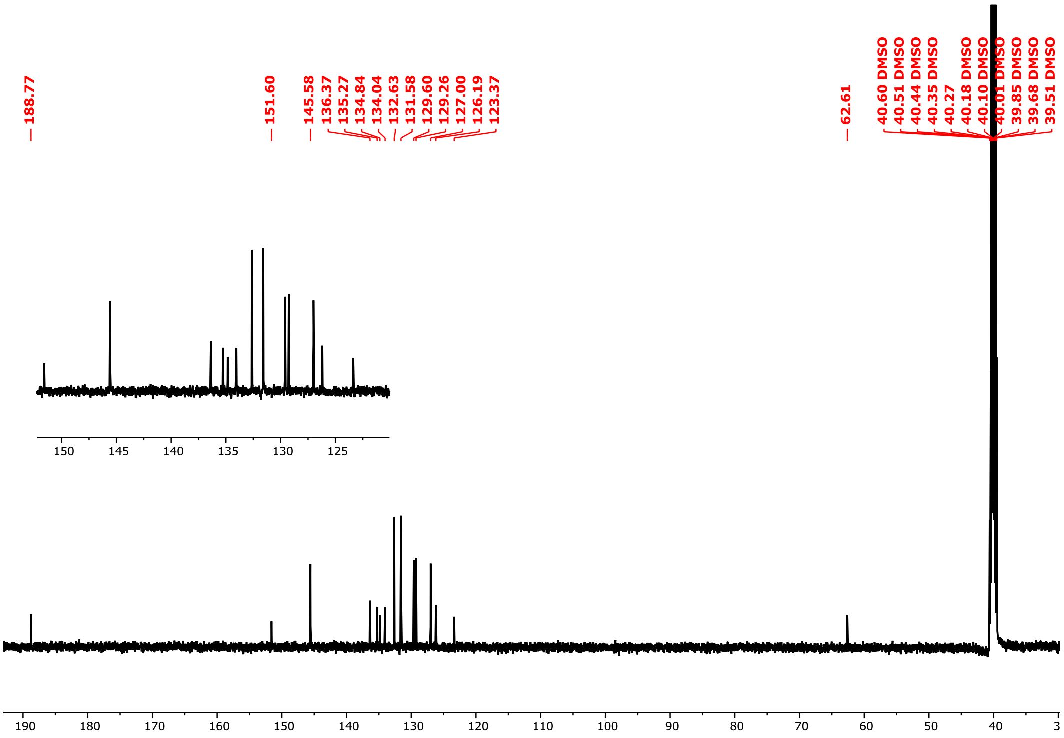

Figure S14: ^{13}C-NMR spectrum of compound **7c** (DMSO-d_6).

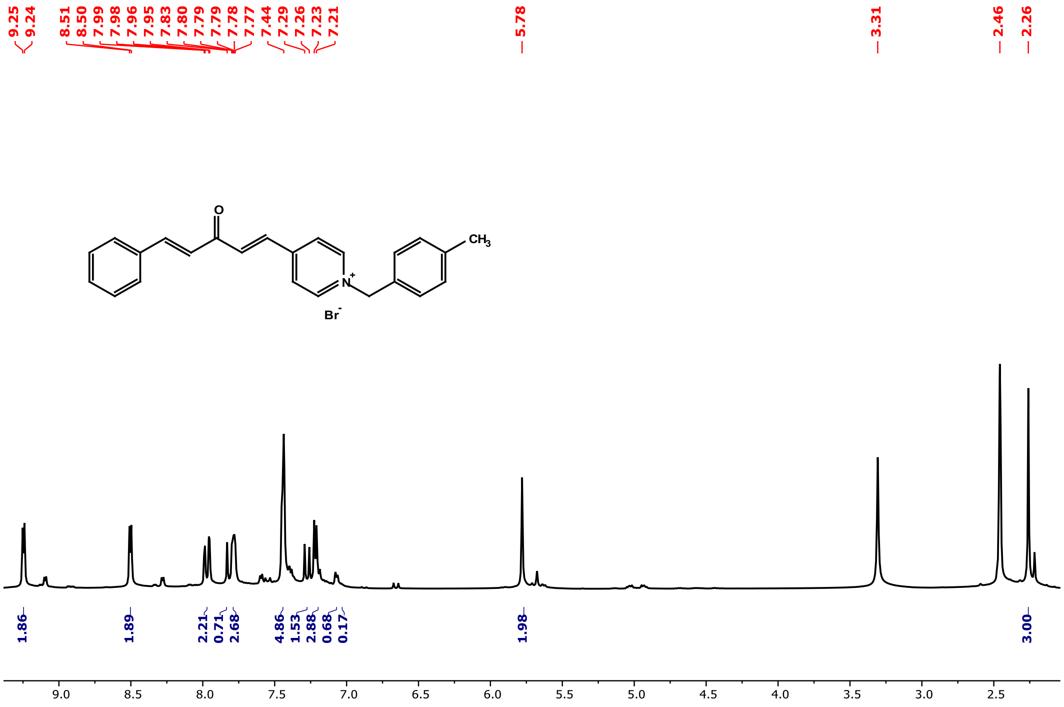

Figure S15: ^{1}H-NMR spectrum of compound **7d** (DMSO-d_6).

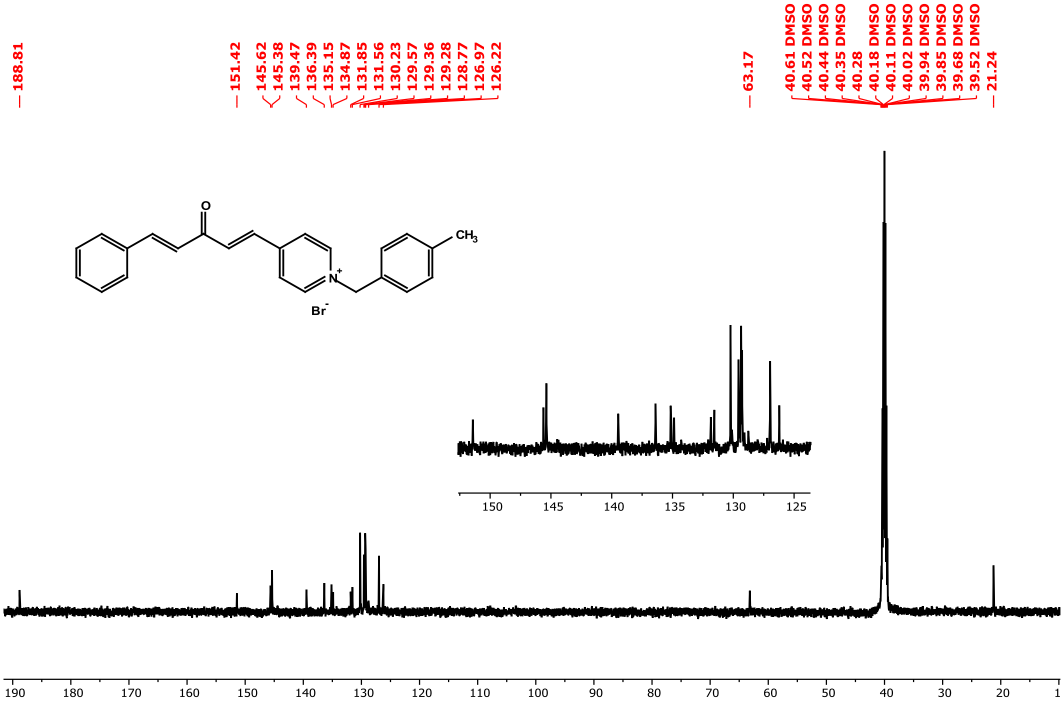

Figure S16: ^{13}C-NMR spectrum of compound **7d** (DMSO-d_6).

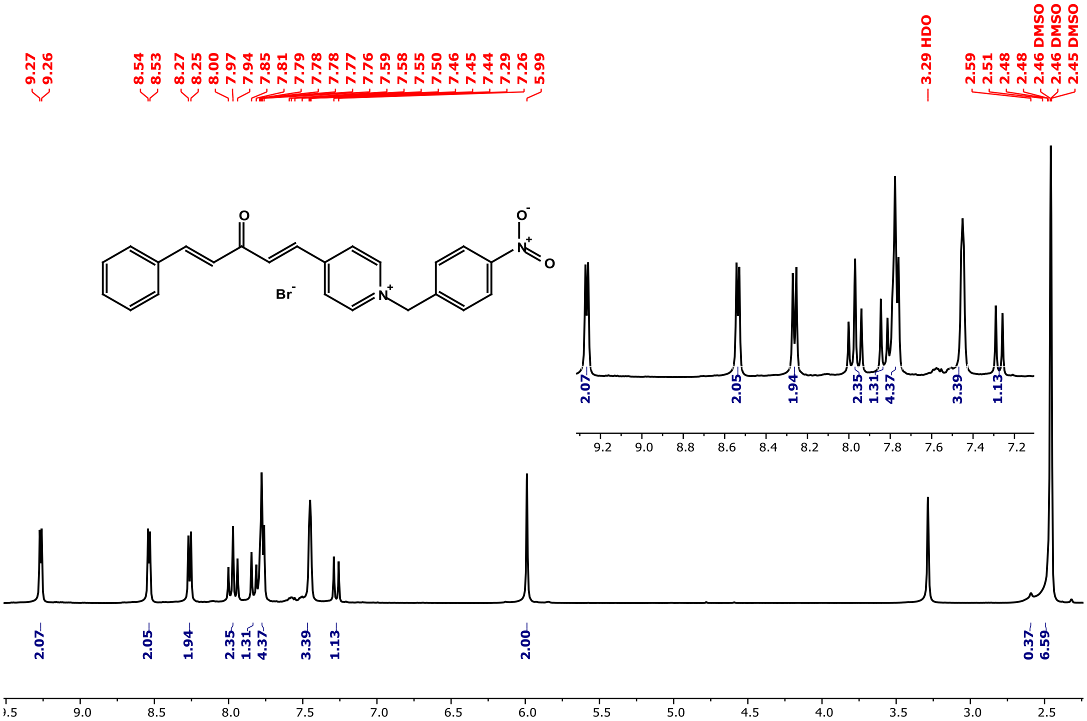

Figure S17: ^{1}H-NMR spectrum of compound **7e** (DMSO-d_6).

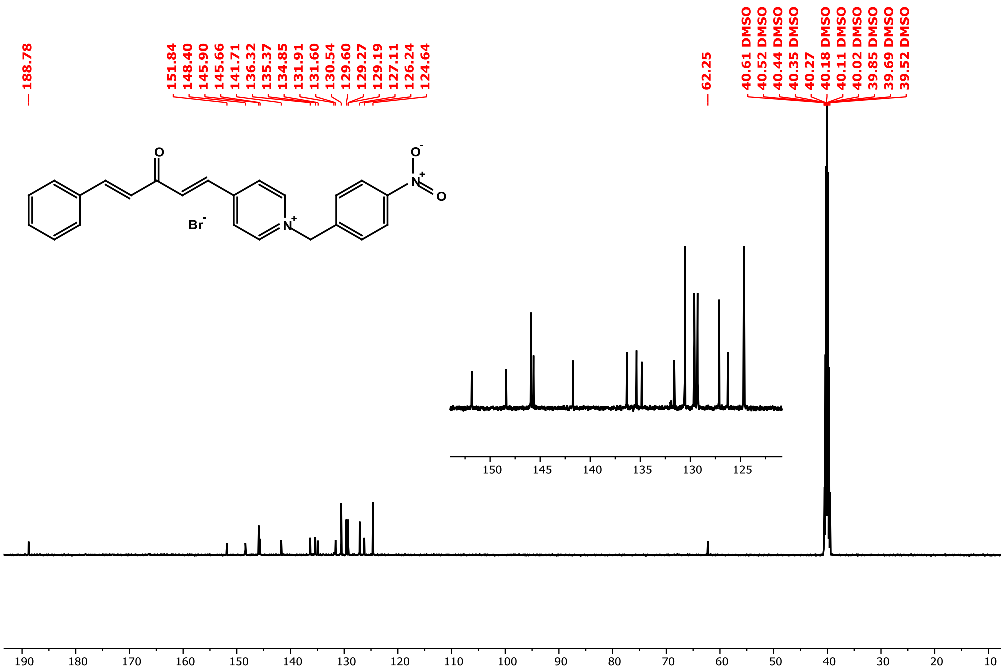

Figure S18: ^{13}C-NMR spectrum of compound **7e** (DMSO-d_6).

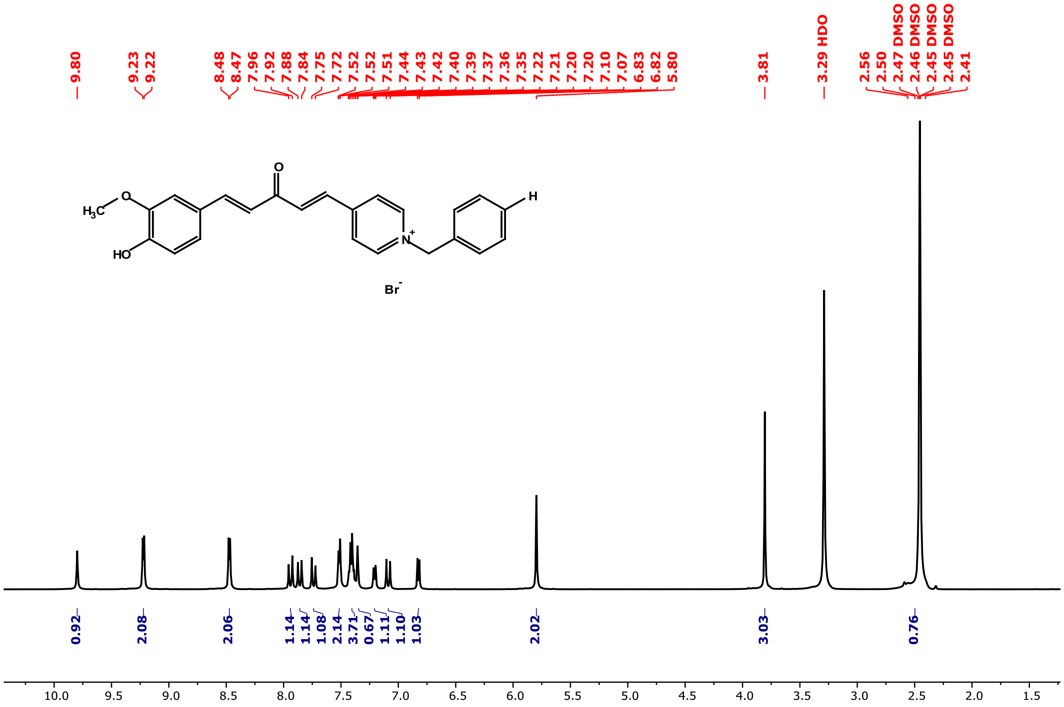

Figure S19: ^{1}H-NMR spectrum of compound **7f** (DMSO-d_6).

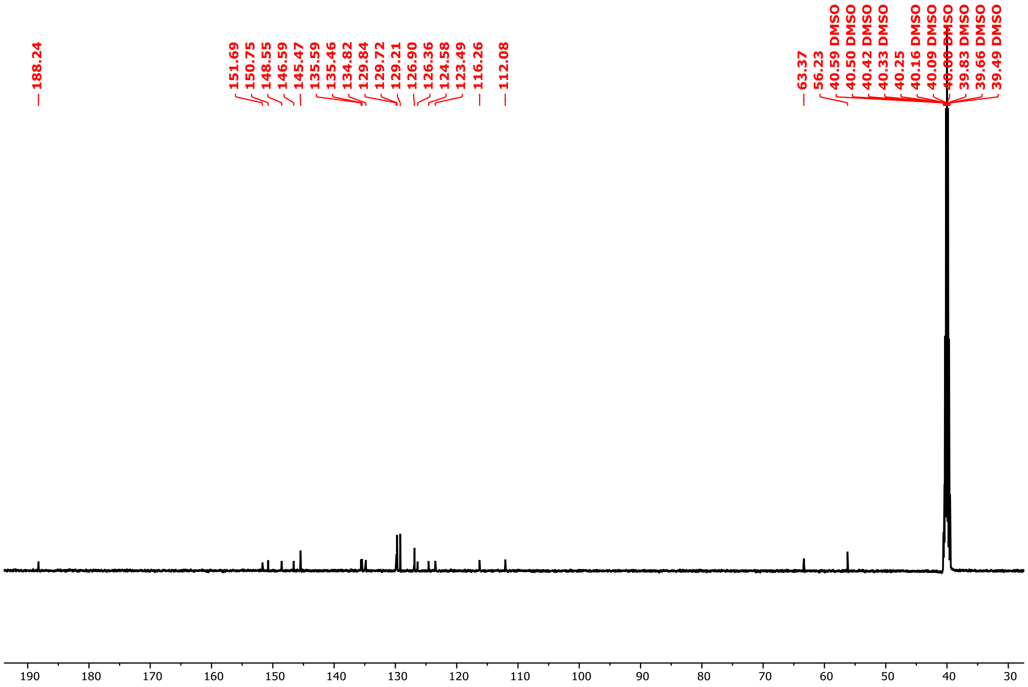

Figure S20: ^{13}C-NMR spectrum of compound **7f** (DMSO-d_6).

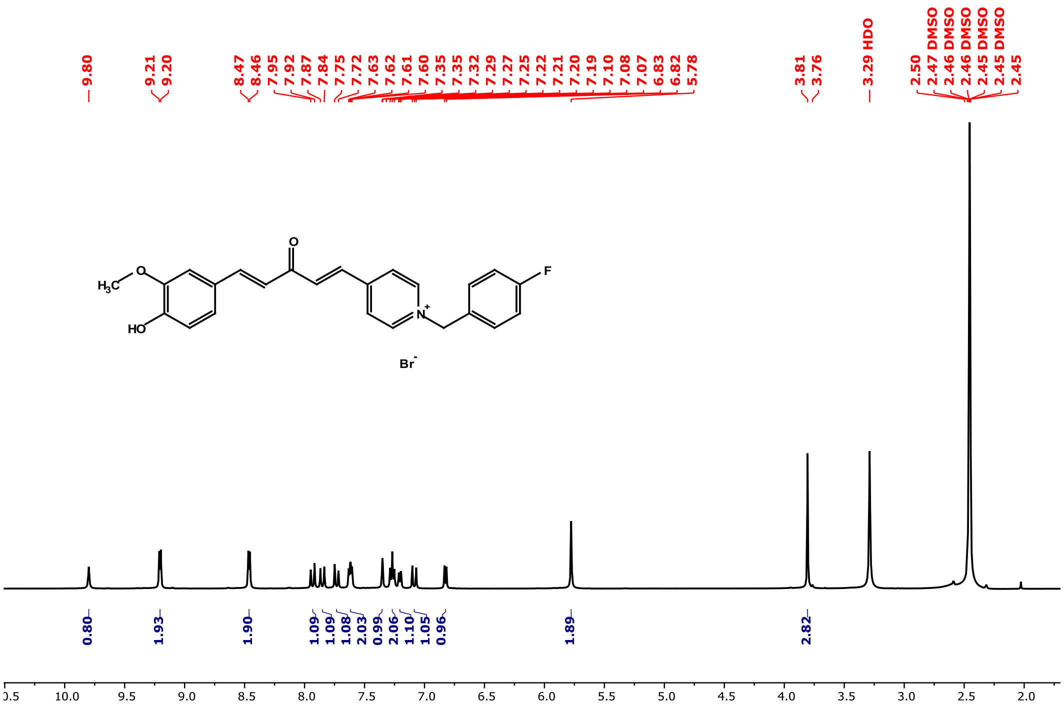

Figure S21: ^{1}H-NMR spectrum of compound **7g** (DMSO-d_6).

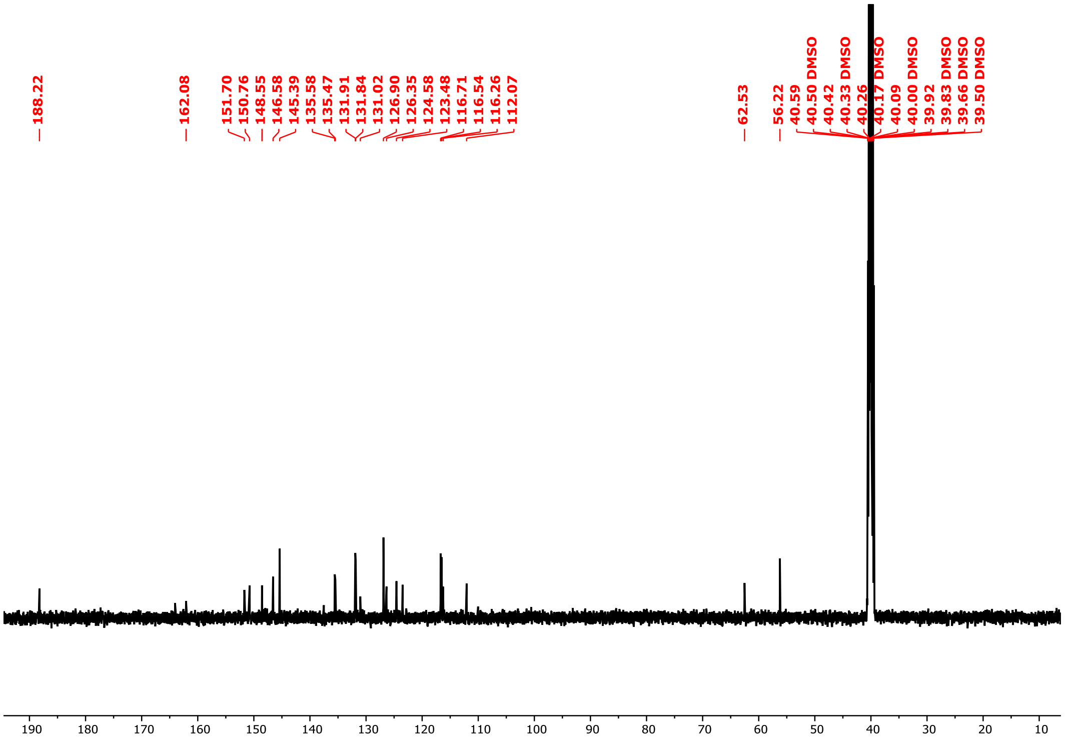

Figure S22: ^{13}C-NMR spectrum of compound **7g** (DMSO-d_6).

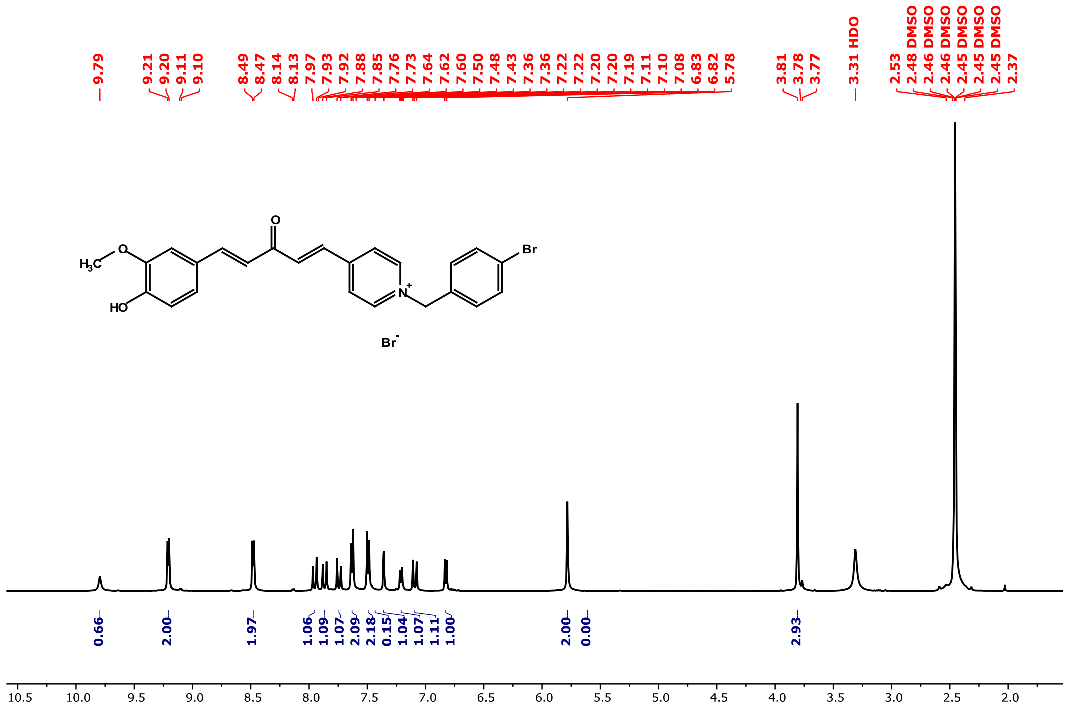

Figure S23: ^{1}H-NMR spectrum of compound **7h** (DMSO-d_6).

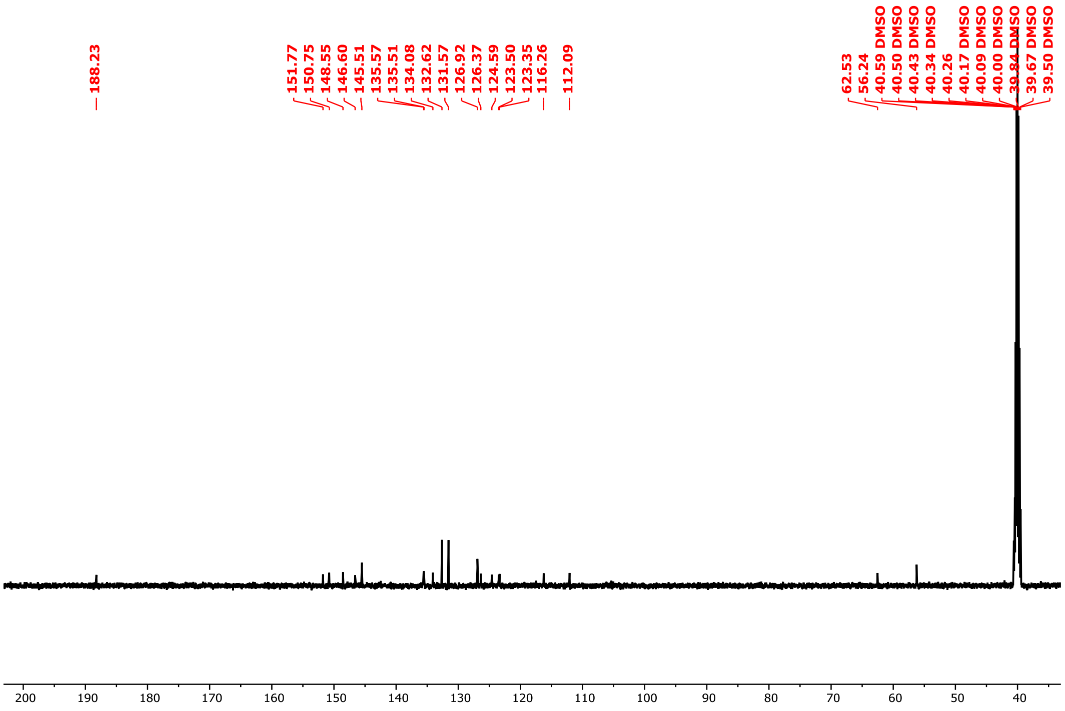

Figure S24: ^{13}C-NMR spectrum of compound **7h** (DMSO-d_6).

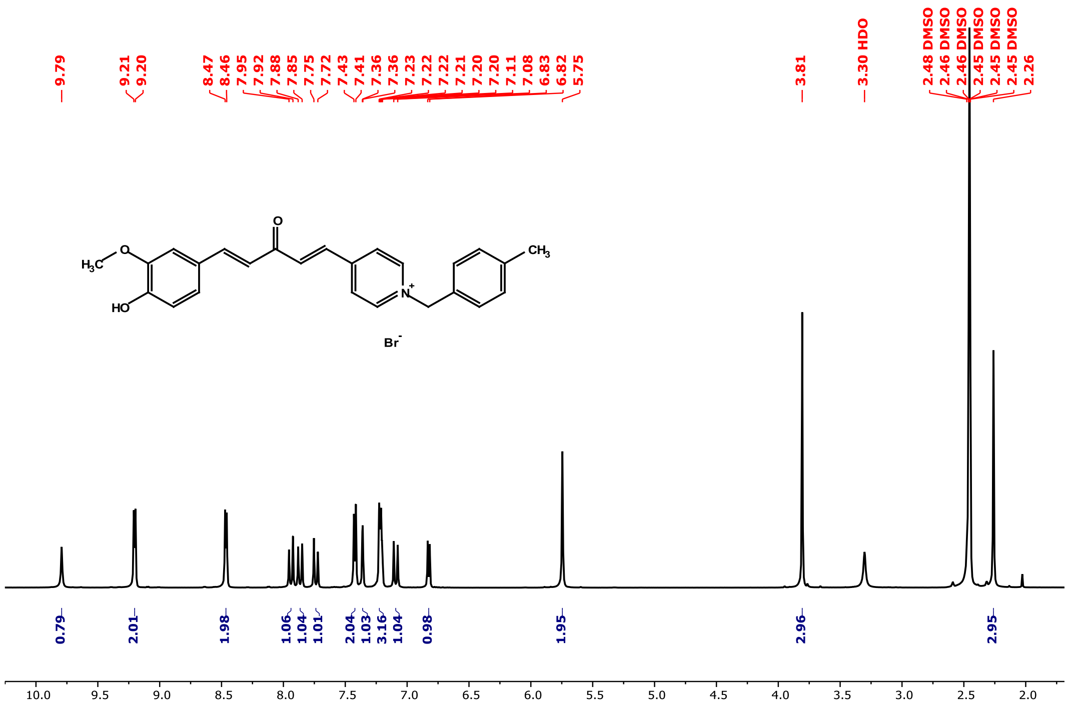

Figure S25: ^{1}H-NMR spectrum of compound **7i** (DMSO-d_6).

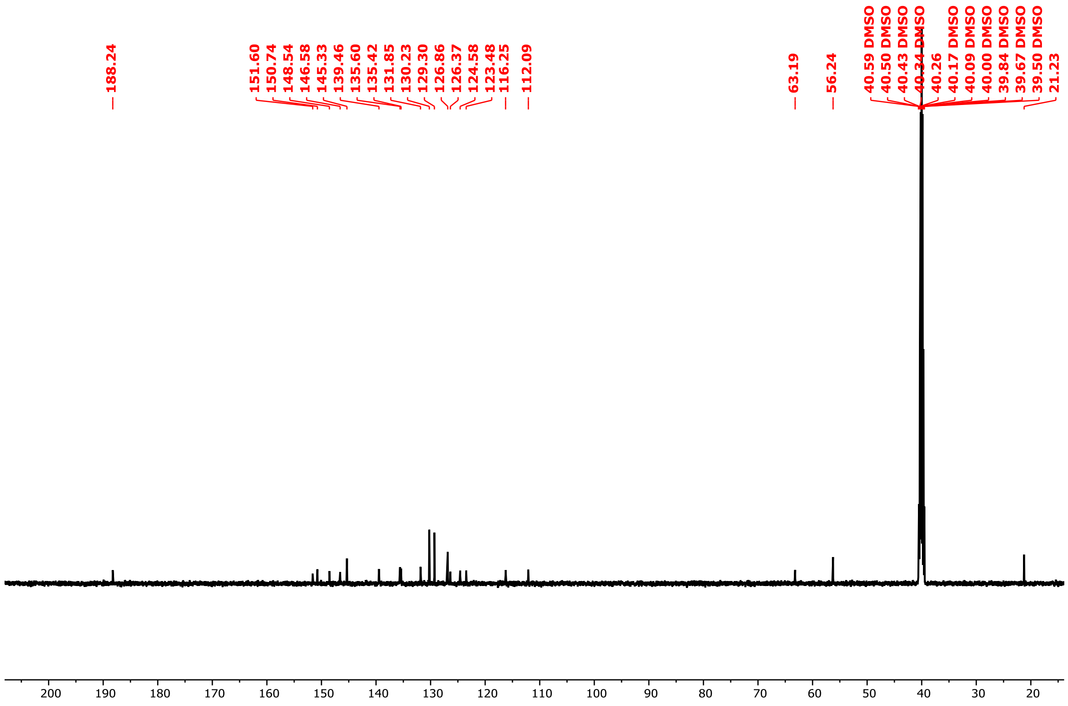

Figure S26: ^{13}C-NMR spectrum of compound **7i** (DMSO-d_6).

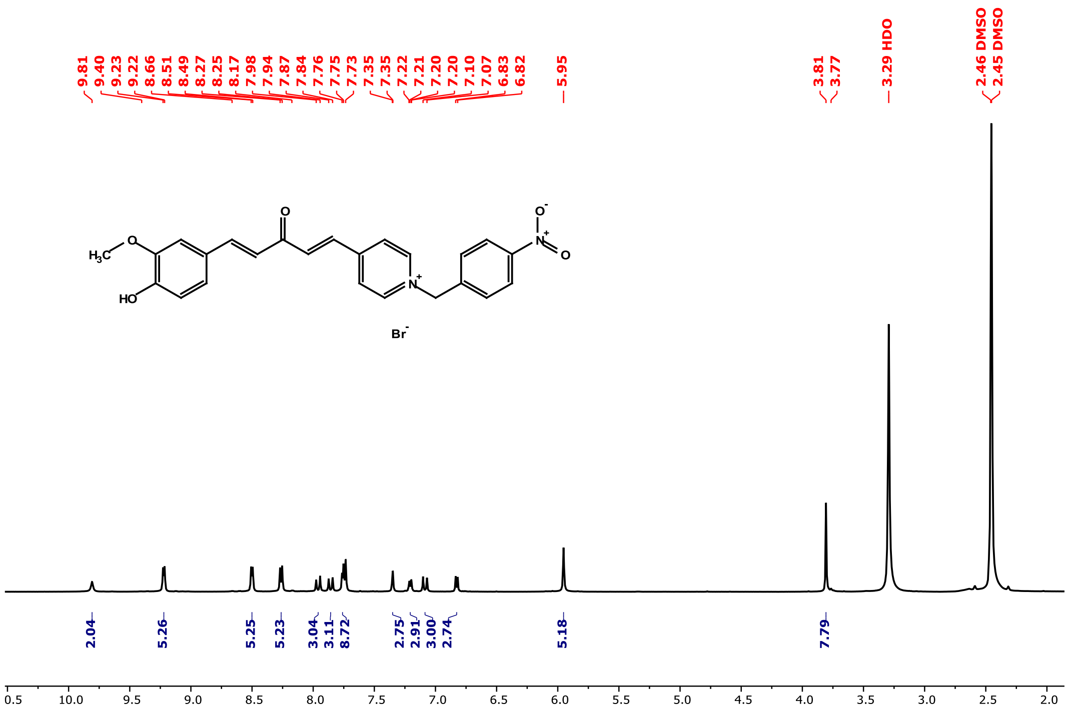

Figure S27: ^{1}H-NMR spectrum of compound **7j** (DMSO-d_6).

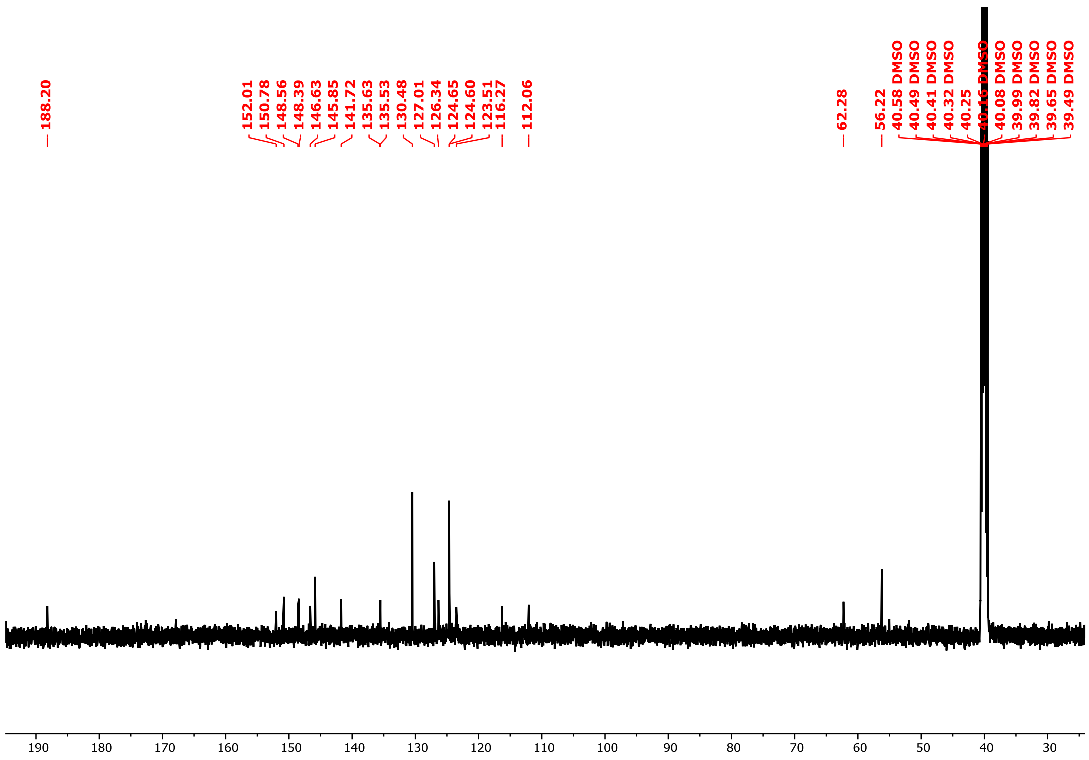

Figure S28: ^{13}C-NMR spectrum of compound **7j** (DMSO-d_6).

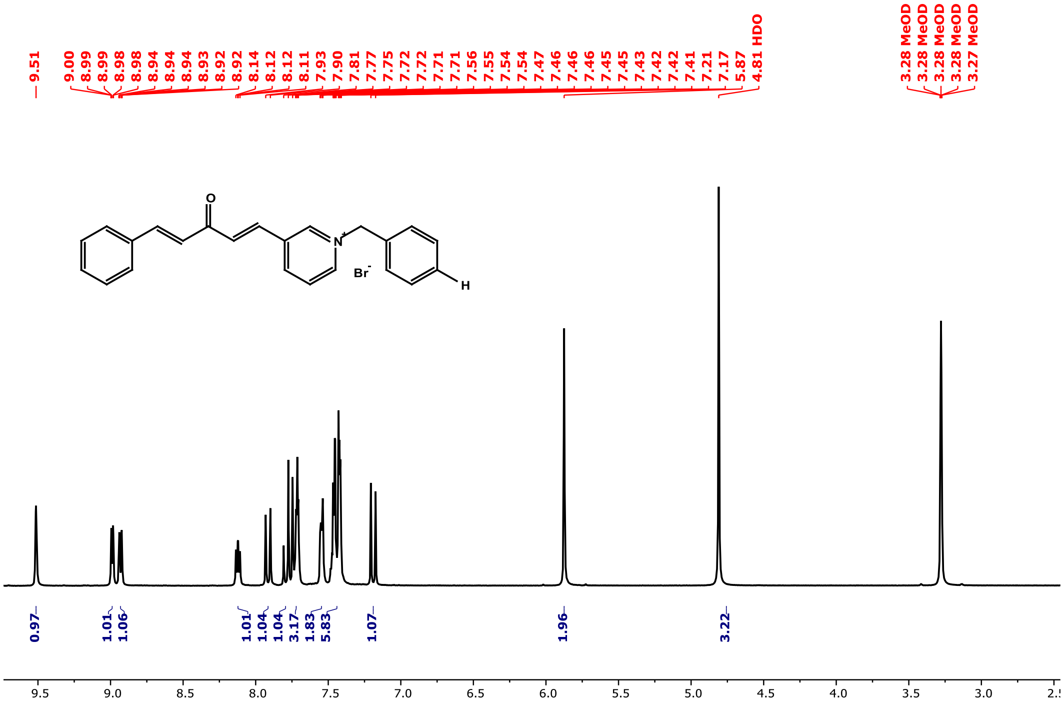

Figure S29: ^{1}H-NMR spectrum of compound **8a** (CD_3OD).

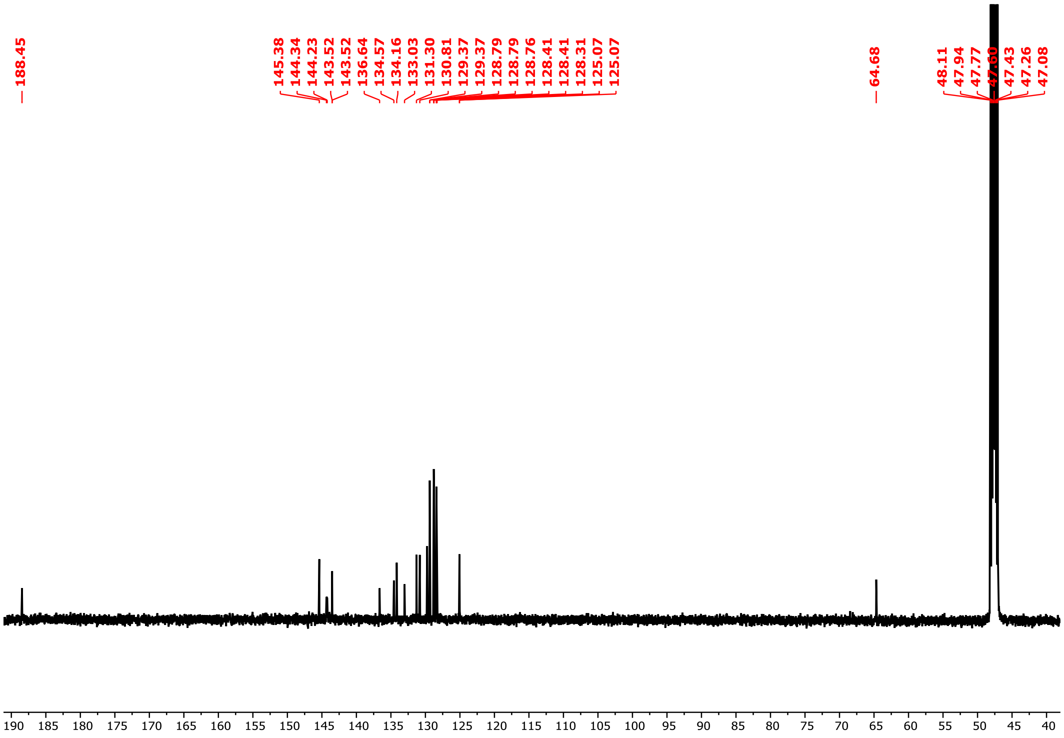

Figure S30: ^{13}C-NMR spectrum of compound **8a** (CD_3OD).

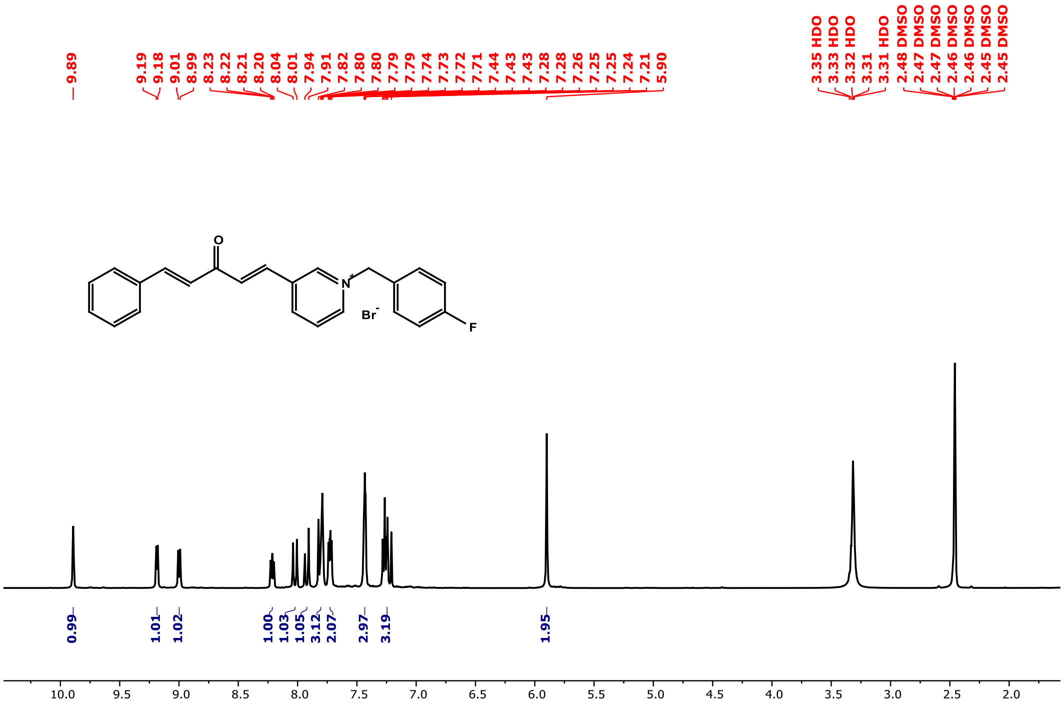

Figure S31: ^{1}H-NMR spectrum of compound **8b** (DMSO-d_6).

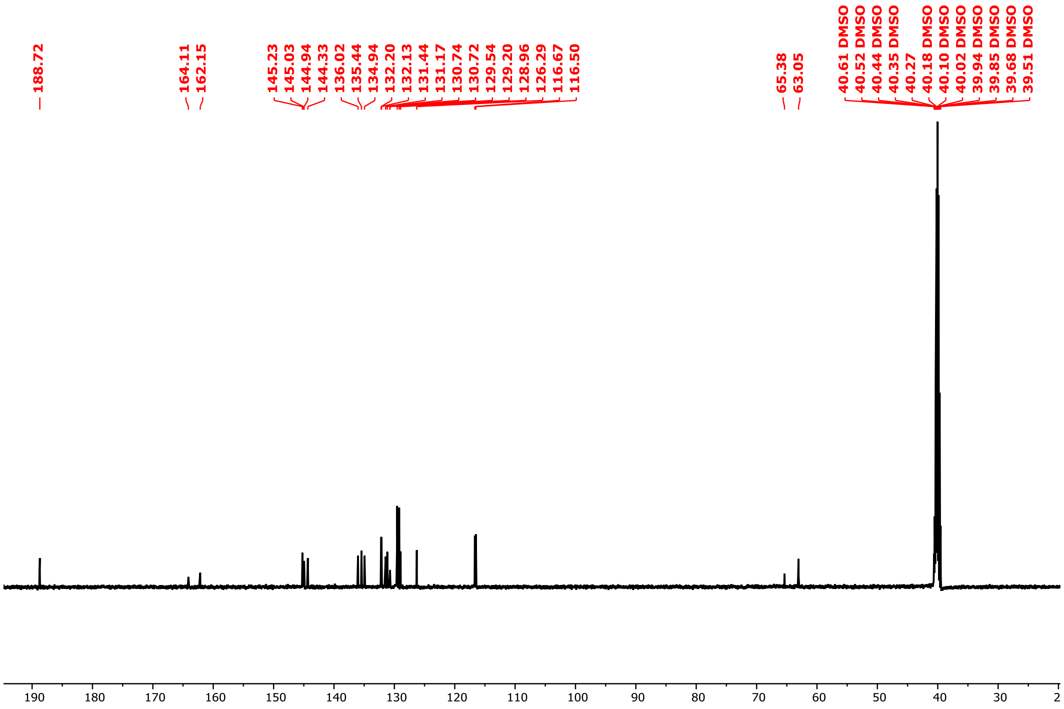

Figure S32: ^{13}C-NMR spectrum of compound **8b** (DMSO-d_6).

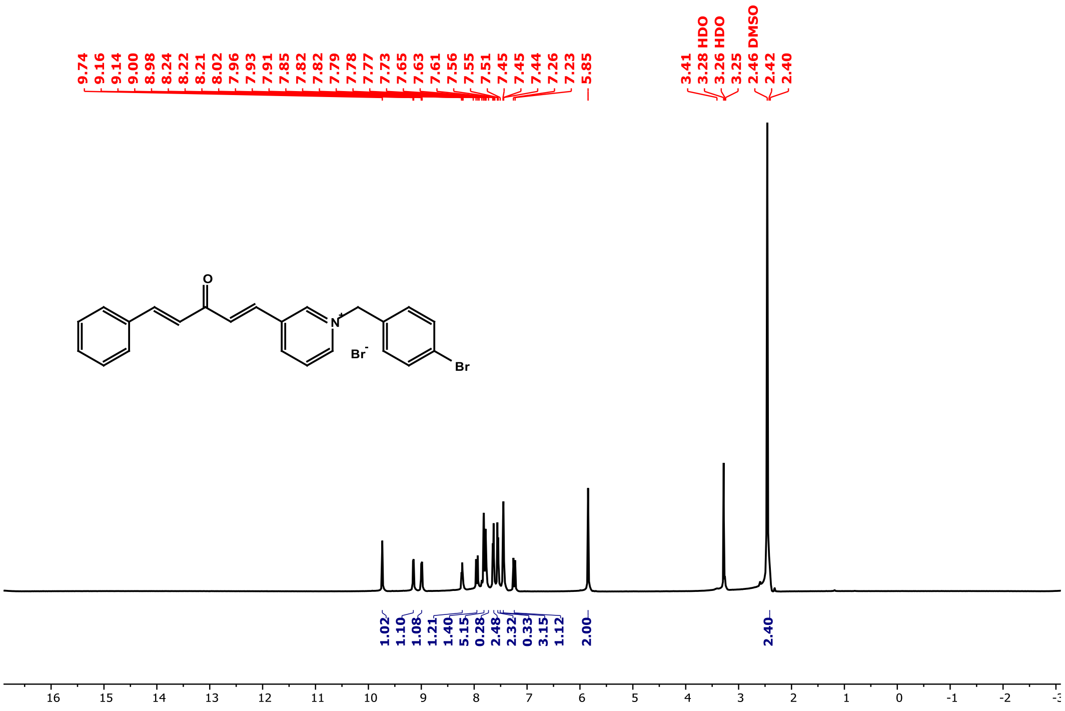

Figure S33: ^{1}H-NMR spectrum of compound **8c** (DMSO-*d*$_6$).

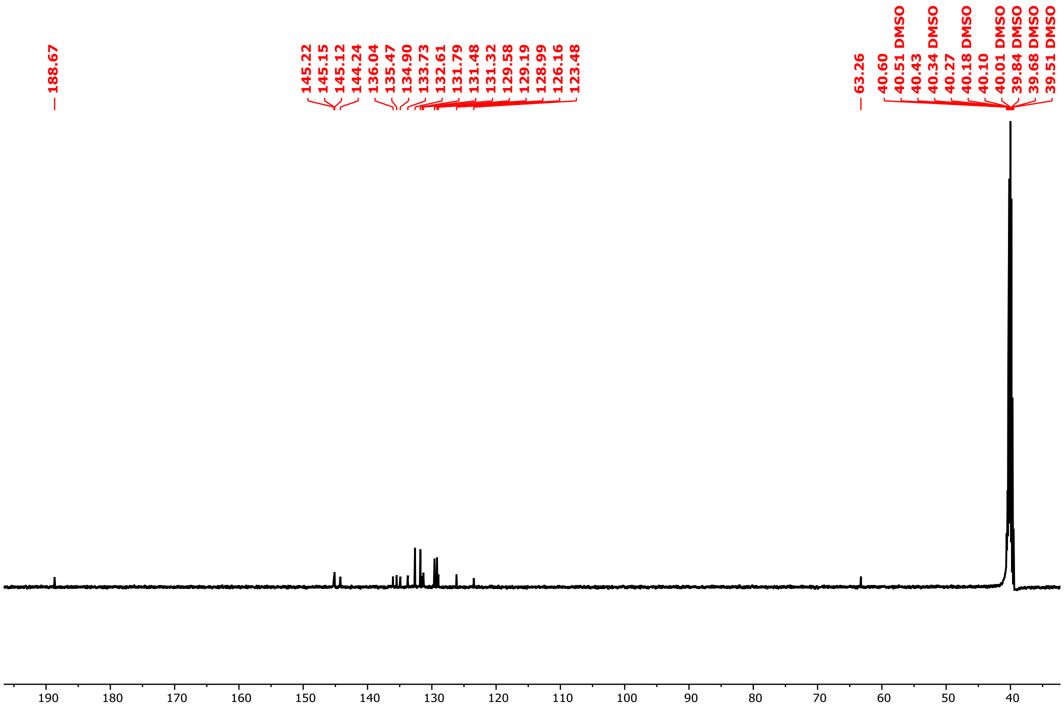

Figure S34: ^{13}C-NMR spectrum of compound **8c** (DMSO-*d*$_6$).

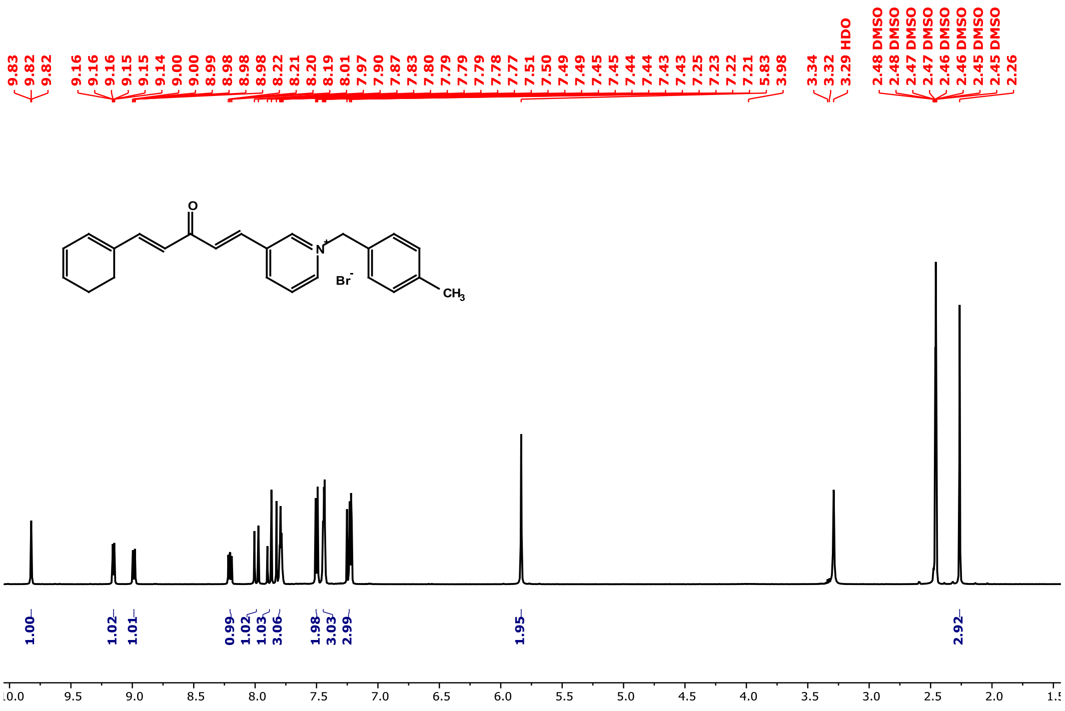

Figure S35: ^{1}H-NMR spectrum of compound **8d** (DMSO-d_6).

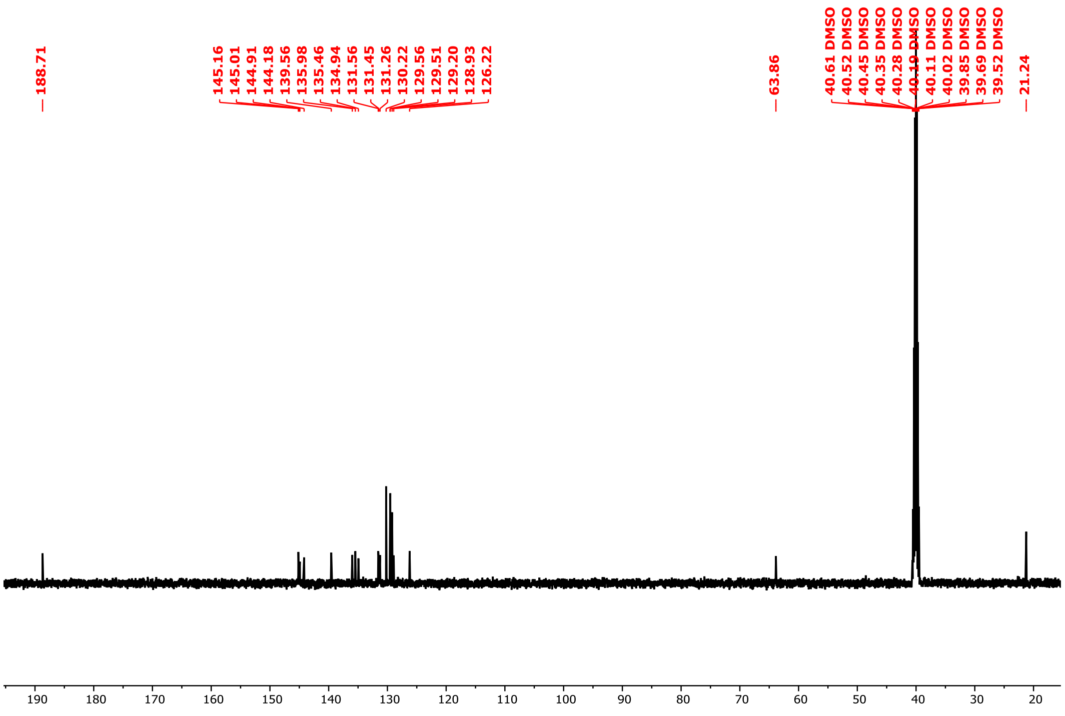

Figure S36: ^{13}C-NMR spectrum of compound **8d** (DMSO-d_6).

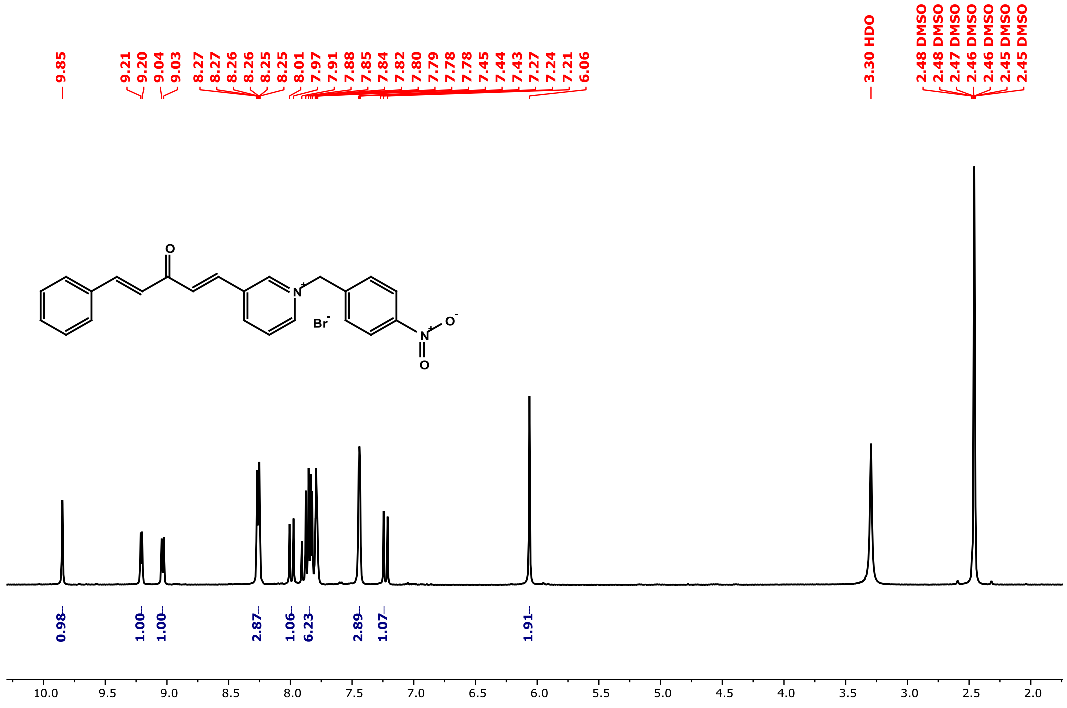

Figure S37: ^{1}H-NMR spectrum of compound **8e** (DMSO-d_6).

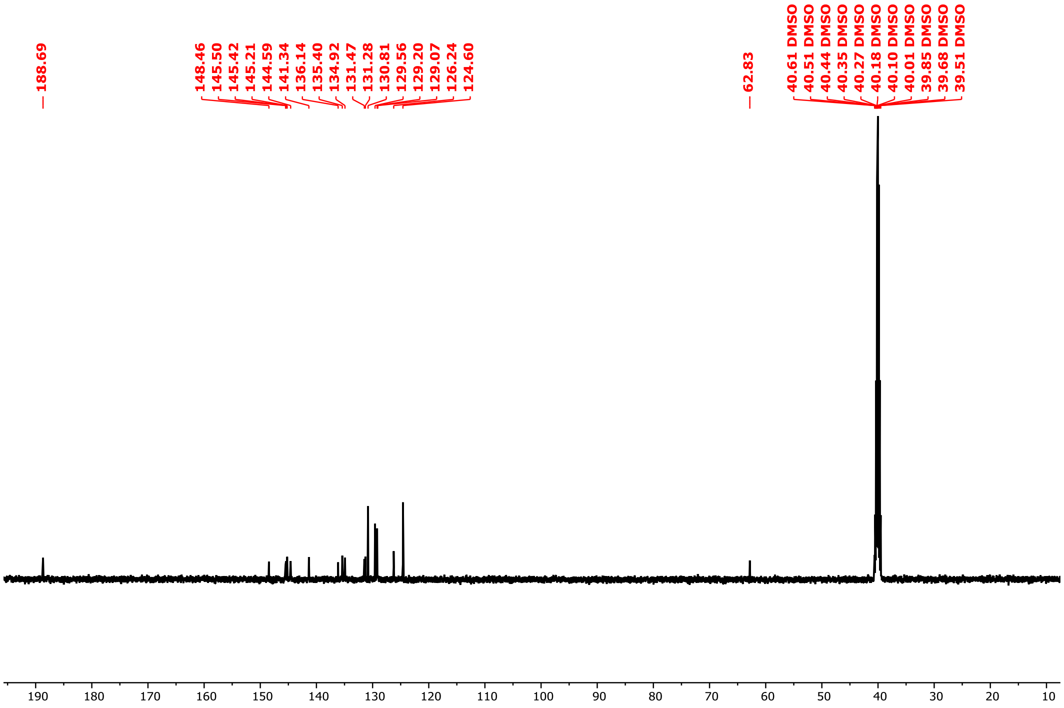

Figure S38: ^{13}C-NMR spectrum of compound **8e** (DMSO-d_6).

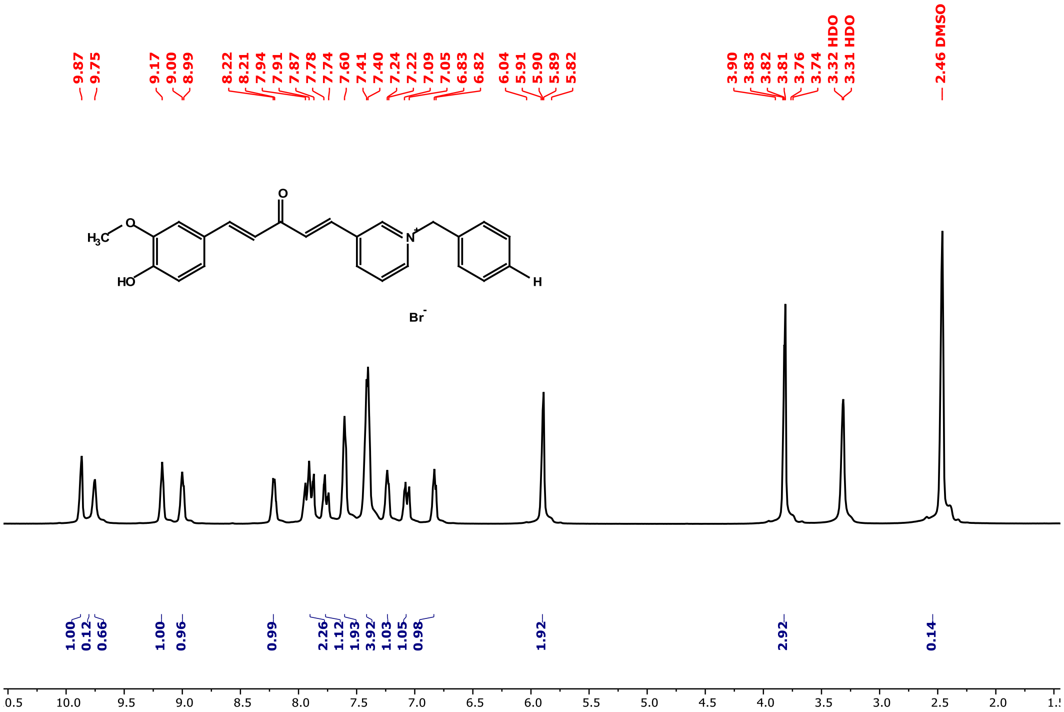

Figure S39: ^{1}H-NMR spectrum of compound **8f** (DMSO-d_6).

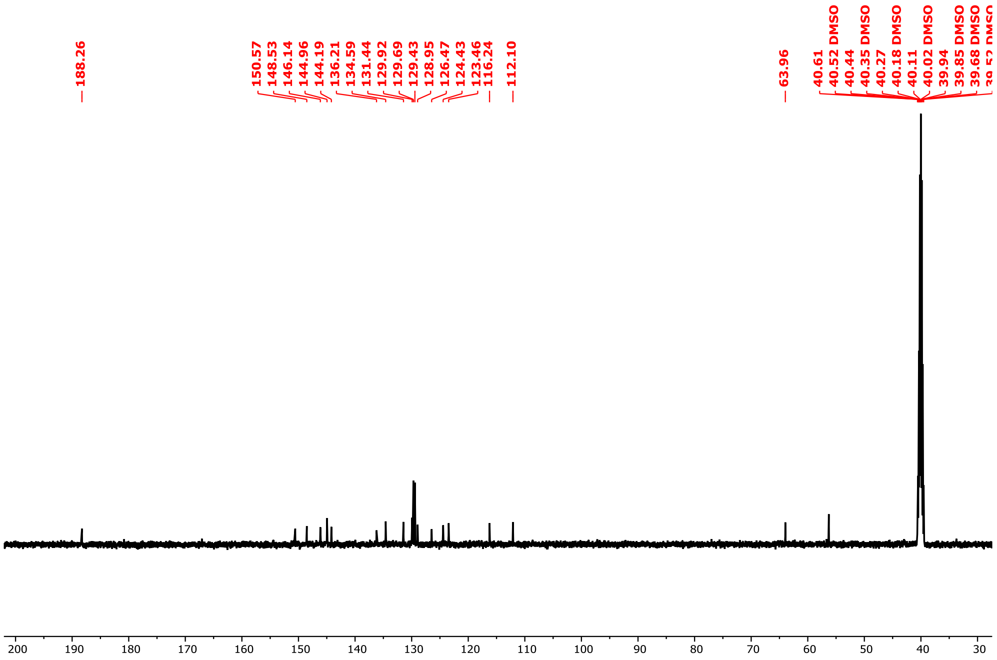

Figure S40: ^{13}C-NMR spectrum of compound **8f** (DMSO-d_6).

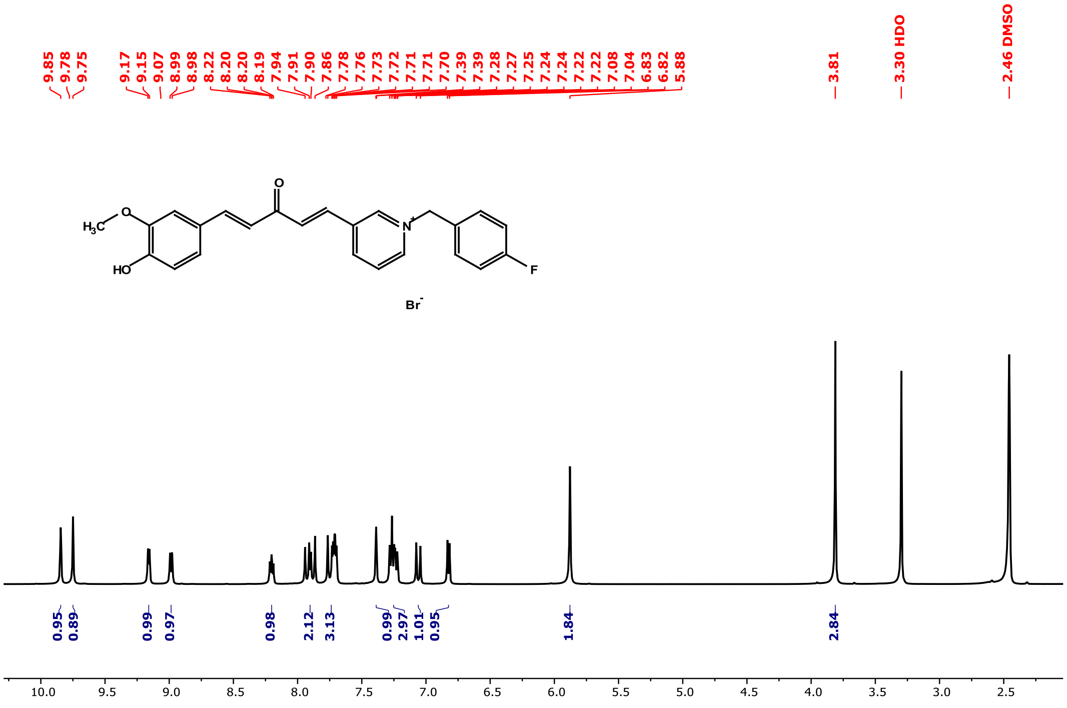

Figure S41: ^{1}H-NMR spectrum of compound **8g** (DMSO-d_6).

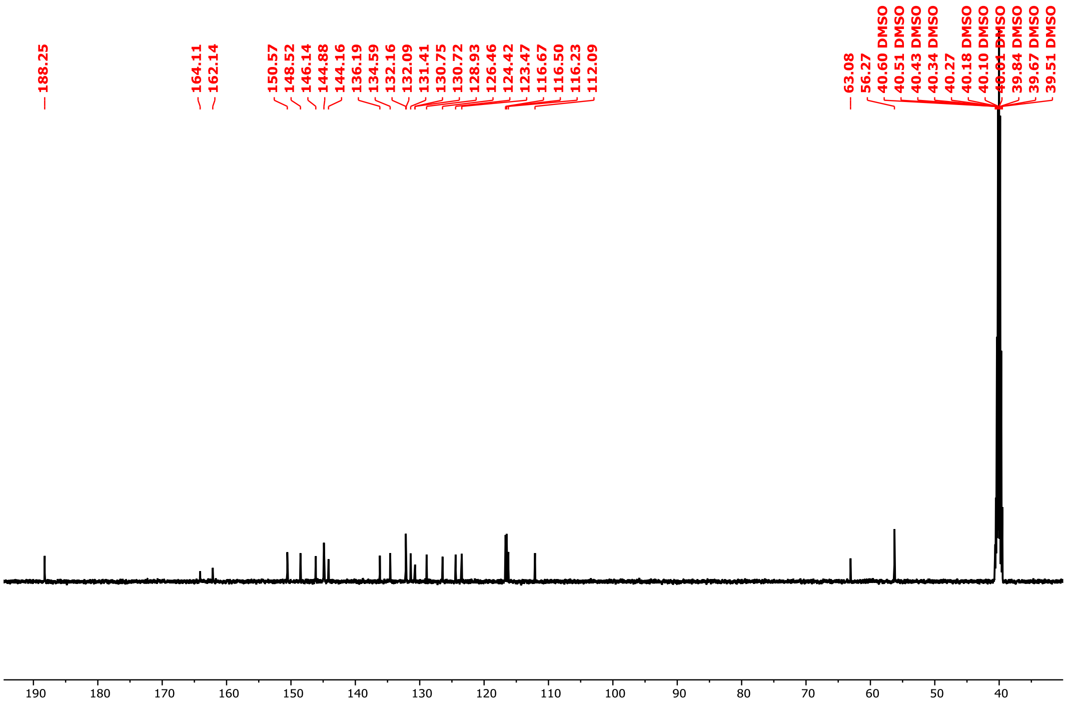

Figure S42: ^{13}C-NMR spectrum of compound **8g** (DMSO-d_6).

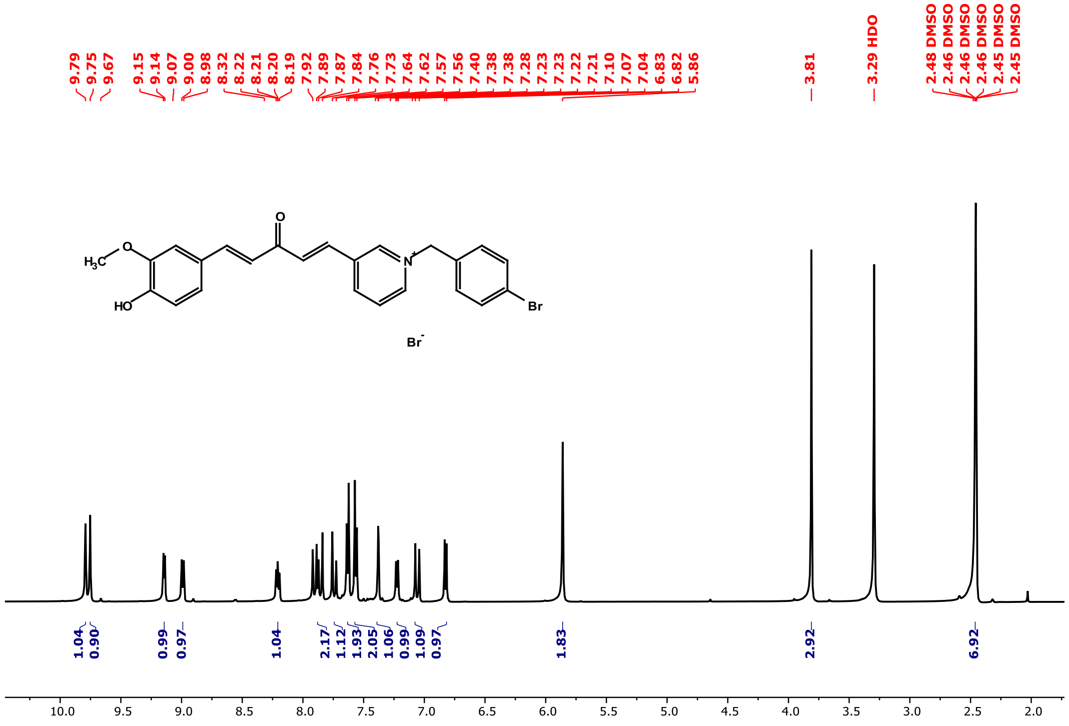

Figure S43: ^{1}H-NMR spectrum of compound **8h** (DMSO-d_6).

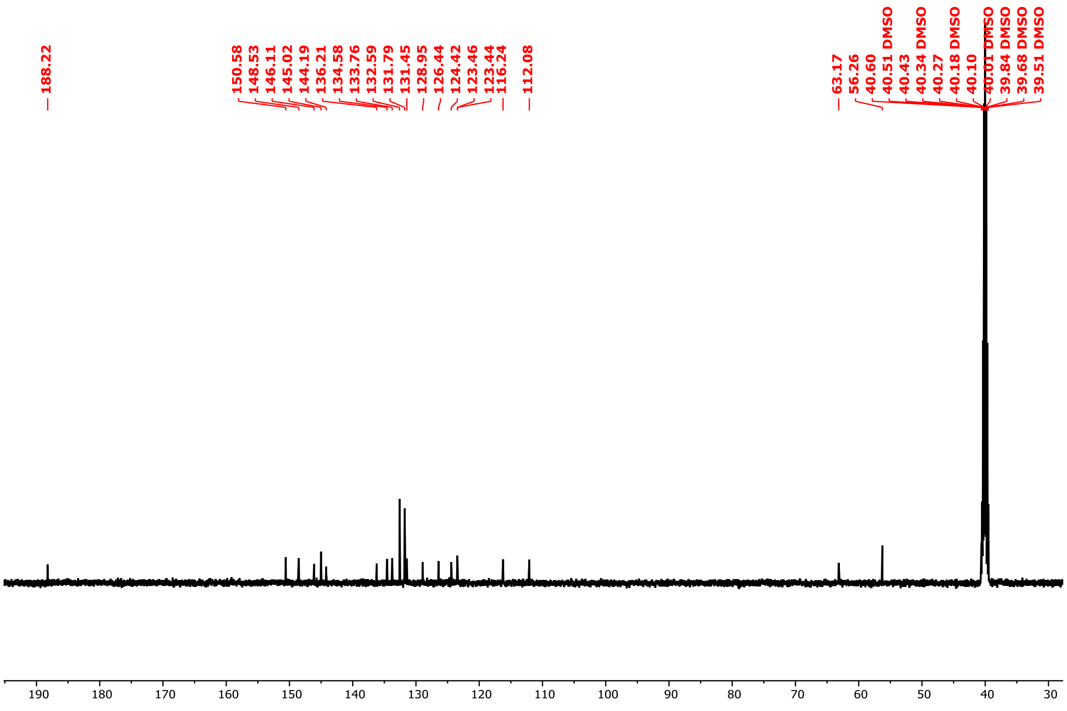

Figure S1: ^{13}C-NMR spectrum of compound **8h** (DMSO-d_6).

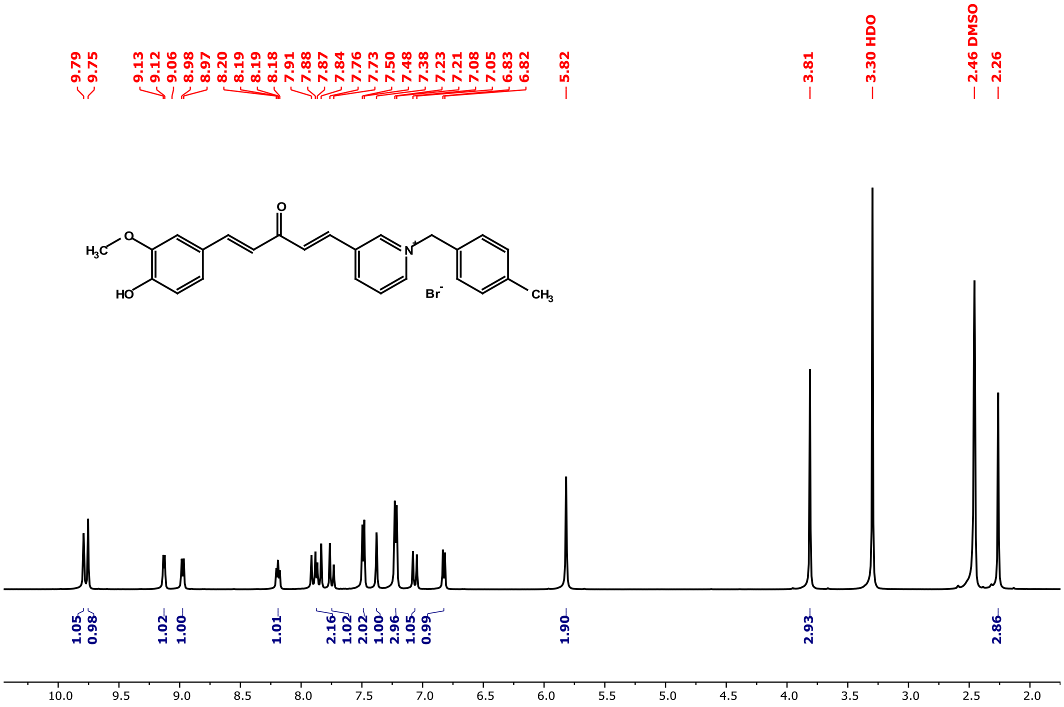

Figure S45: ^{1}H-NMR spectrum of compound **8i** (DMSO-d_6).

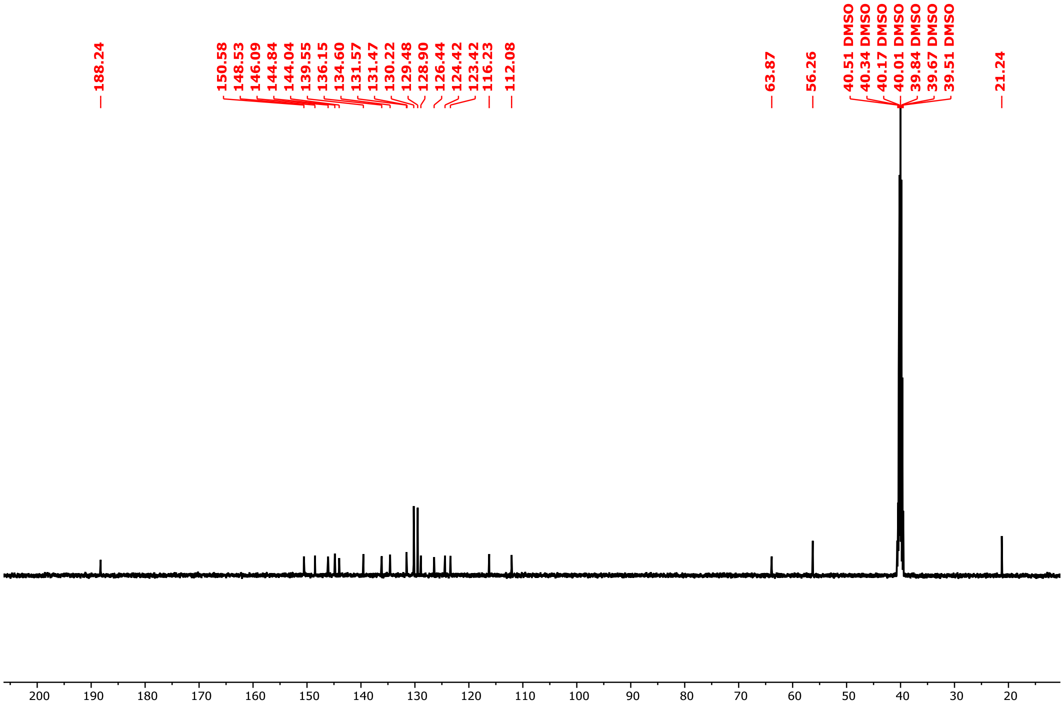

Figure S46: ^{13}C-NMR spectrum of compound **8i** (DMSO-d_6).

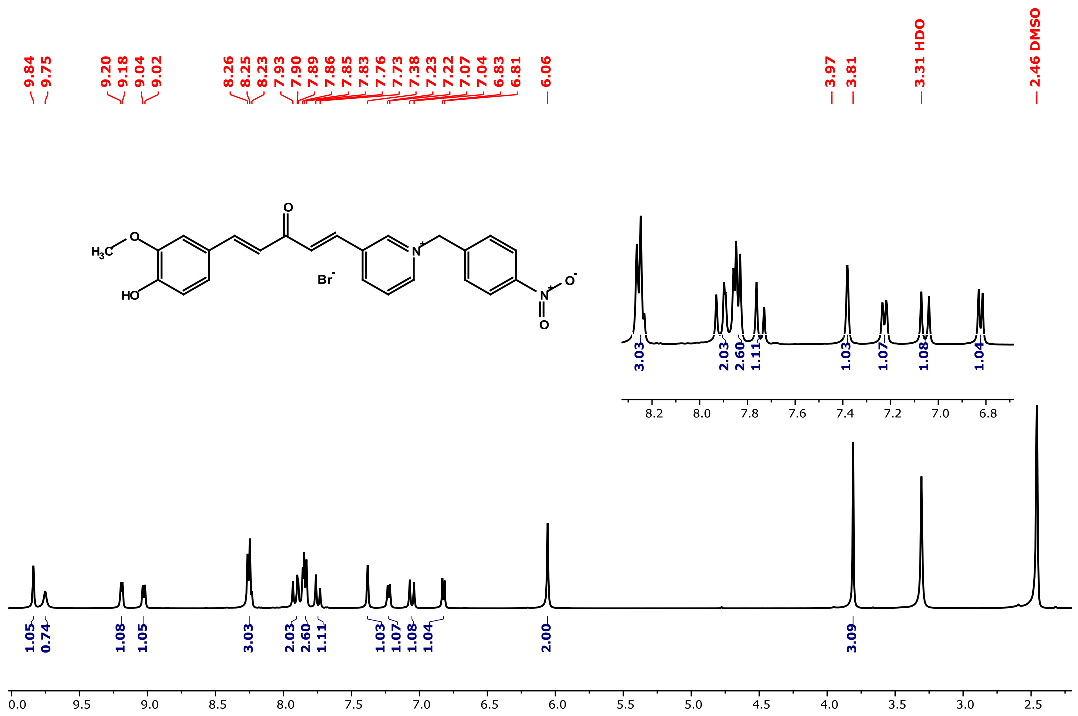

Figure S47: ^{1}H-NMR spectrum of compound **8j** (DMSO-d_6).

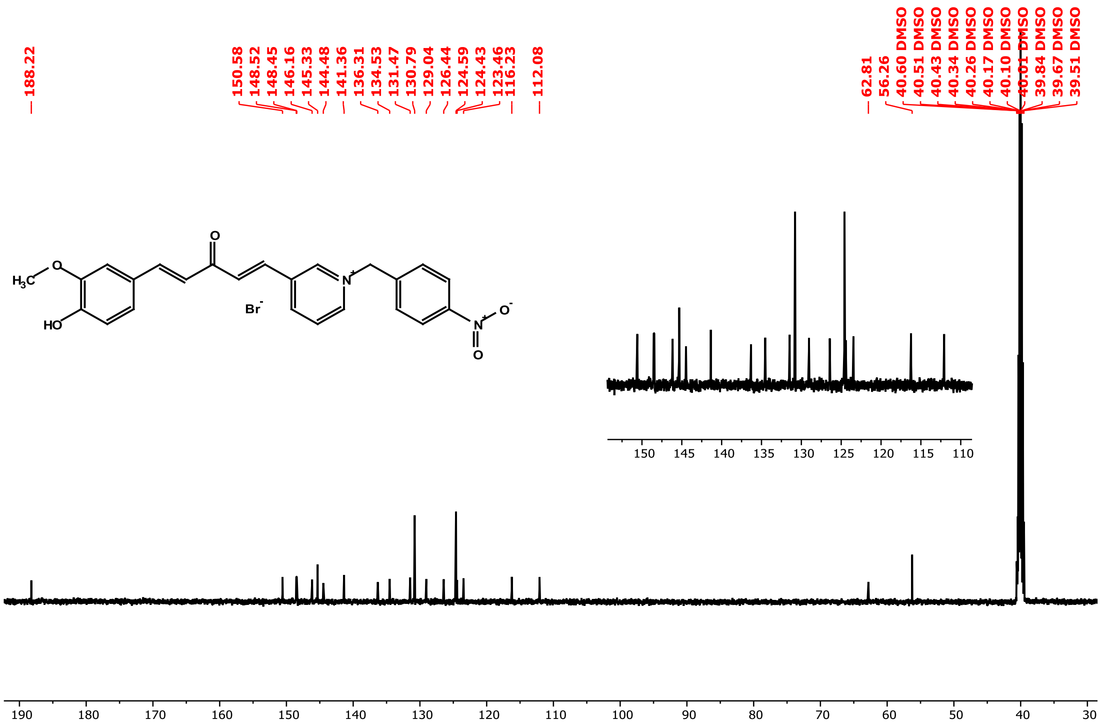

Figure S48: ^{13}C-NMR spectrum of compound **8j** (DMSO-d_6).

2. Inhibition assay graphs

2.1.Dose-Response curves

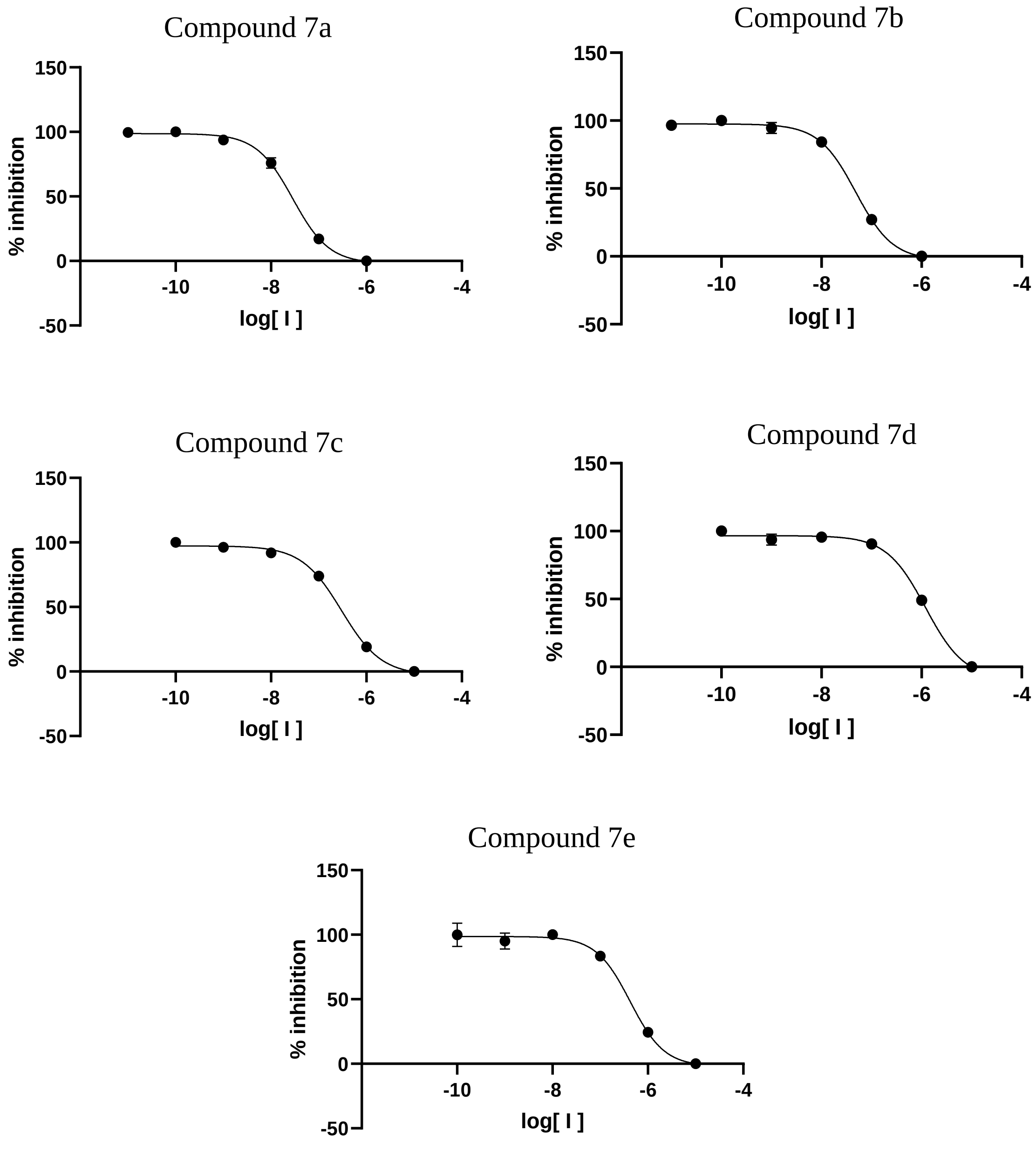

Figure S49-A: IC_{50} value curves of benzene-based compounds bearing 4-benzyl pyridinium moiety.

Compound 8a

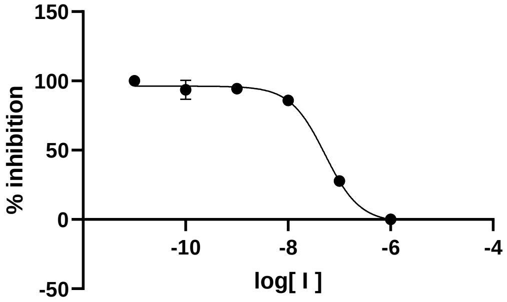

Compound 8b

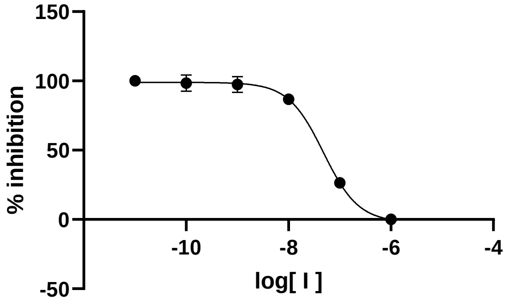

Compound 8c

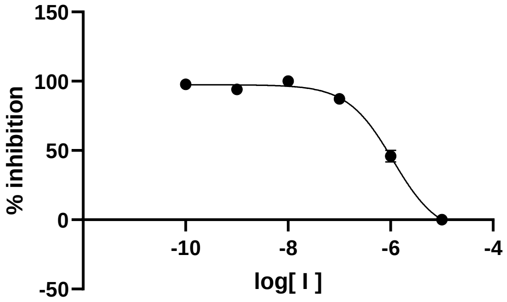

Compound 8d

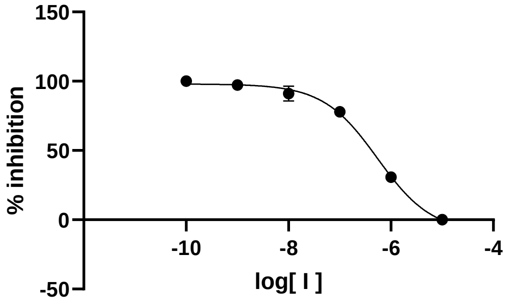

Compound 8e

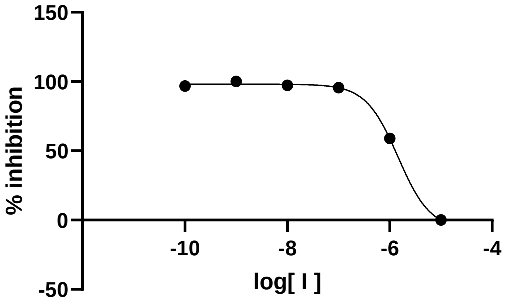

Figure S49-B: IC_{50} value curves of benzene-based compounds bearing 3-benzyl pyridinium moiety.

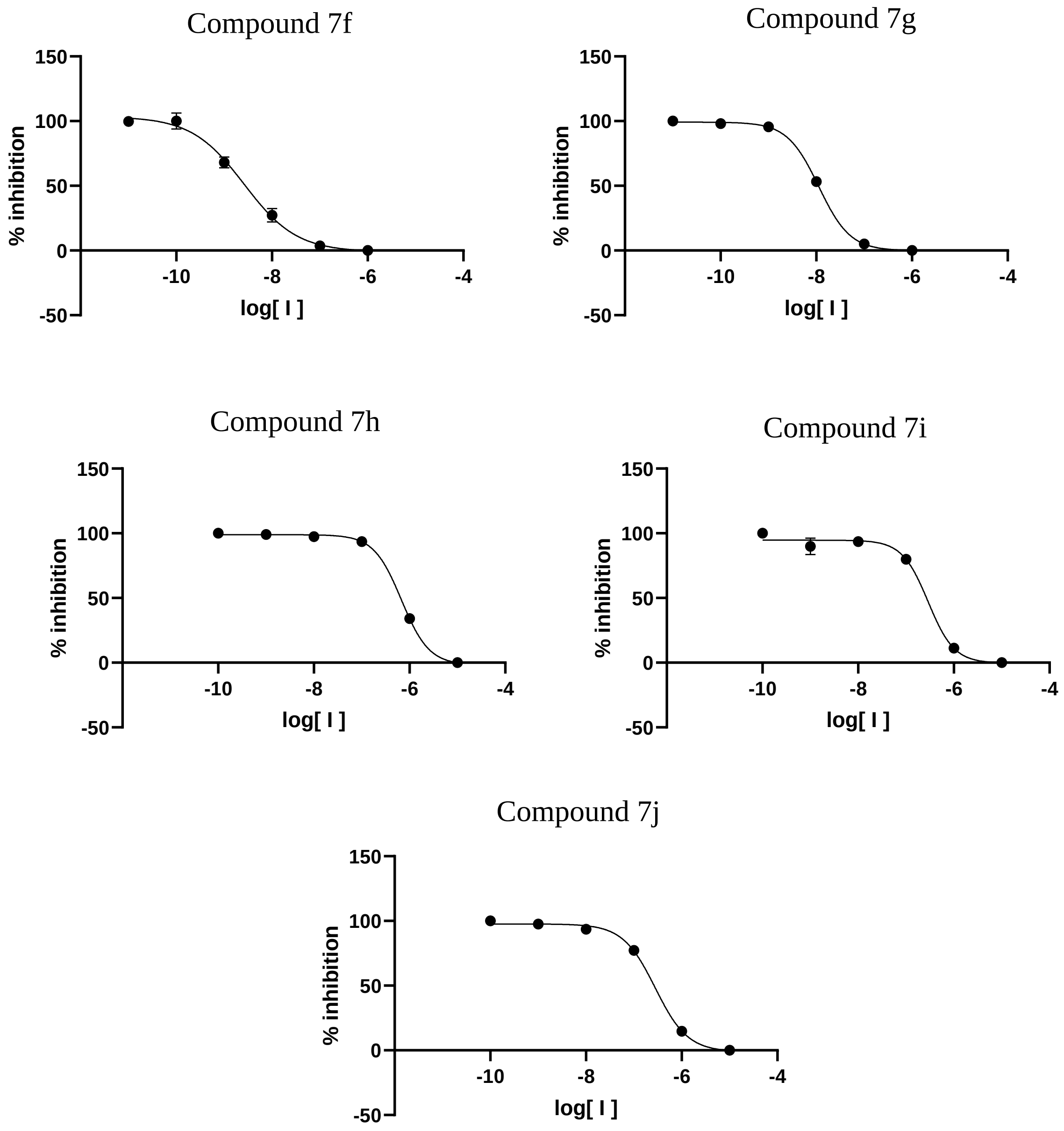

Figure S49-C: Dose-response curves of curcumin-based compounds bearing 4-benzyl pyridinium moiety.

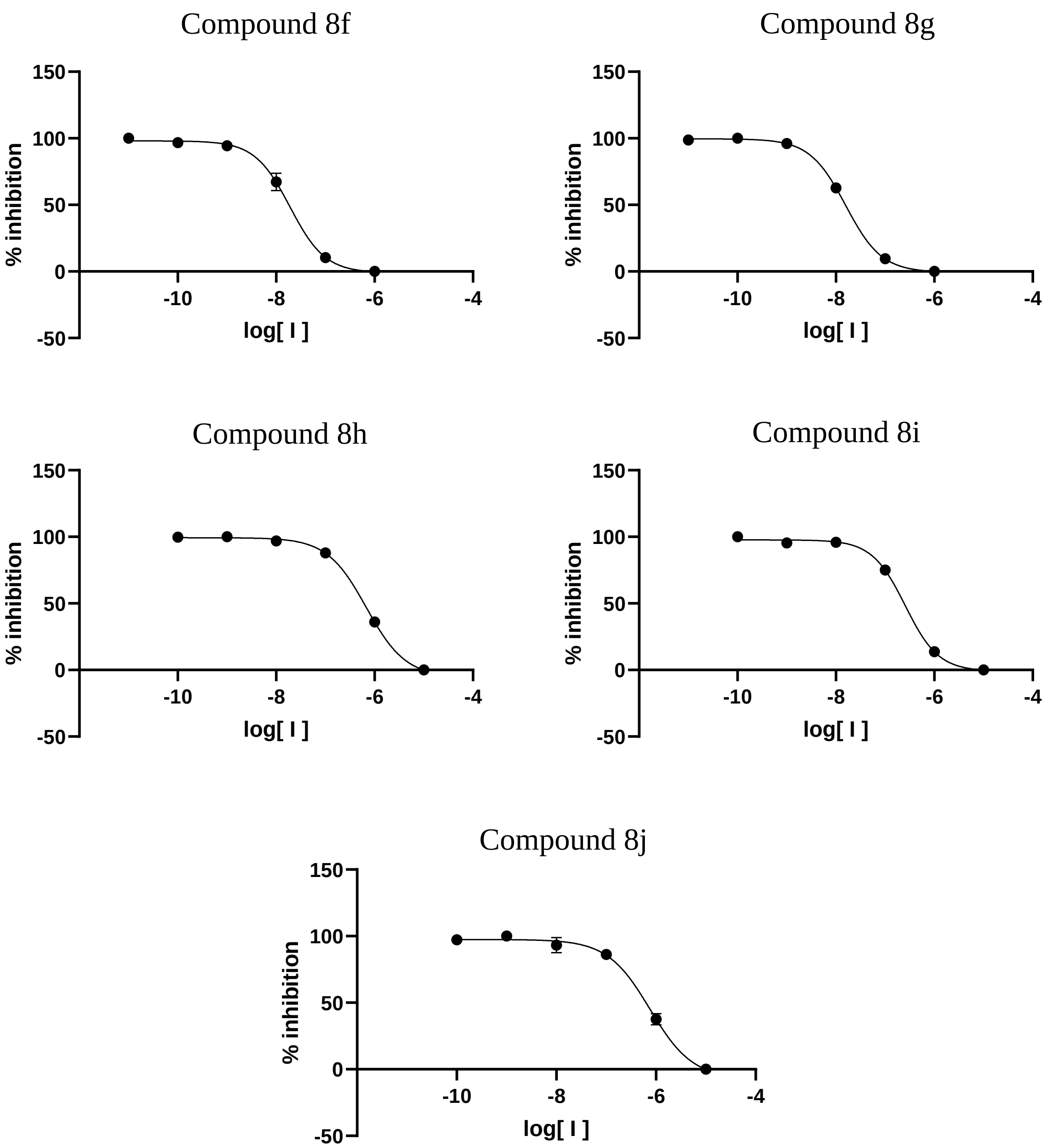

Figure S49-D: Dose-response curves of curcumin-based compounds bearing 3-benzyl pyridinium moiety.

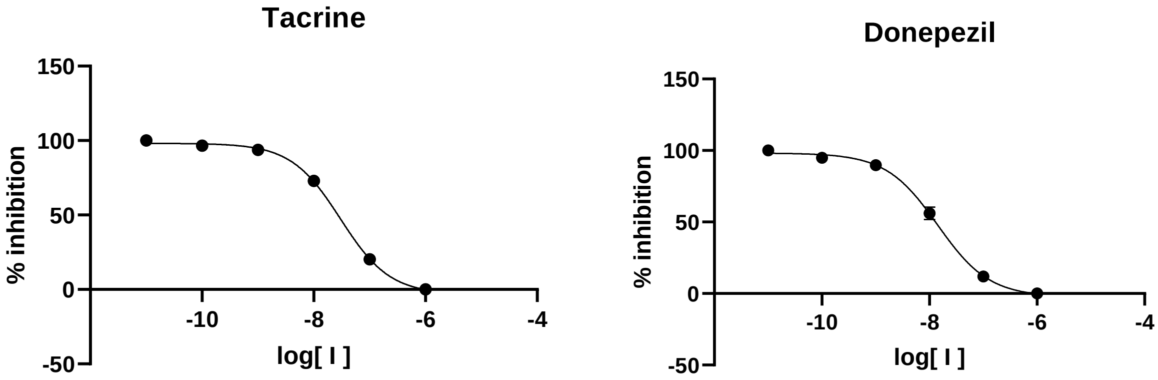

Figure S49-E: Dose-response curves of standard drugs Tacrine/Donepezil.

2.2. Kinetics study graphs

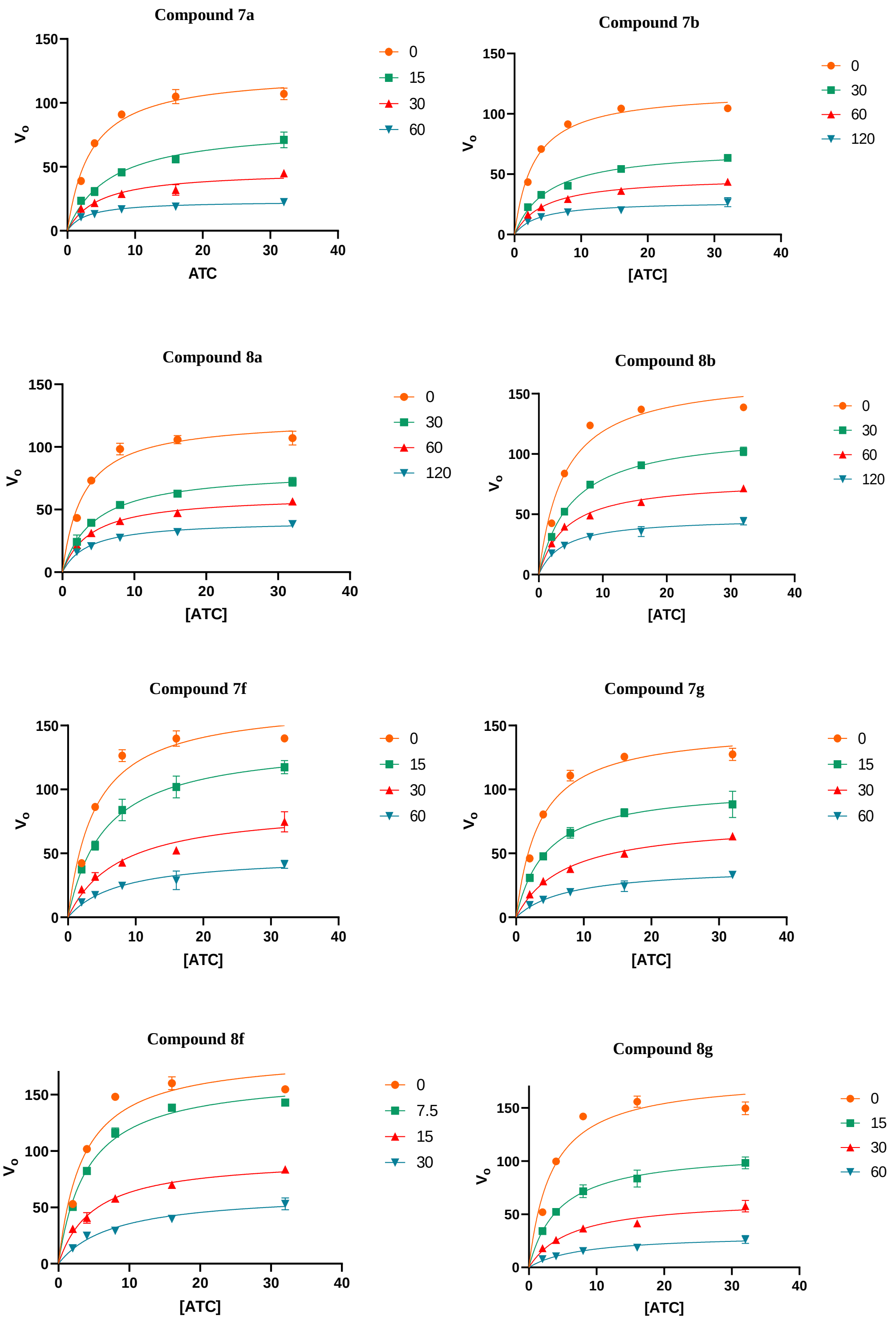

Figure S50-A: Michaelis-Menten kinetics plot of the most potent compounds in the presence and absence of compound (inhibitor) (0-120 nM).

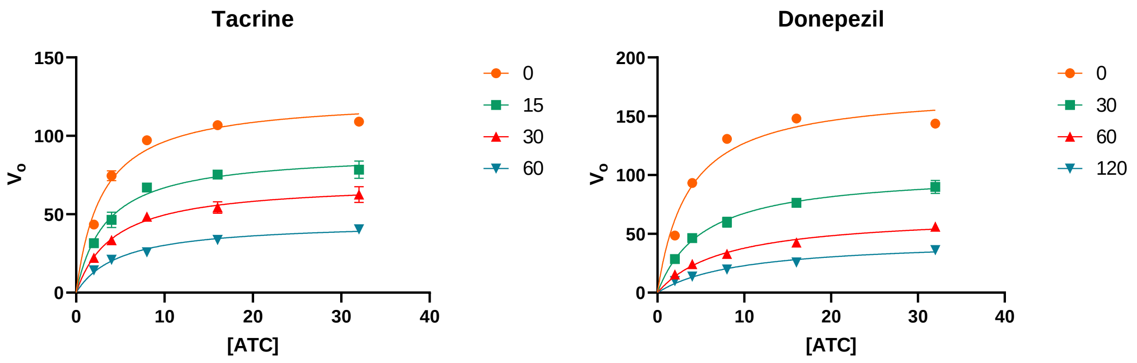

Figure S50-B: Michaelis-Menten kinetics plot of standard drugs tacrine and donepezil (0-120 nM).

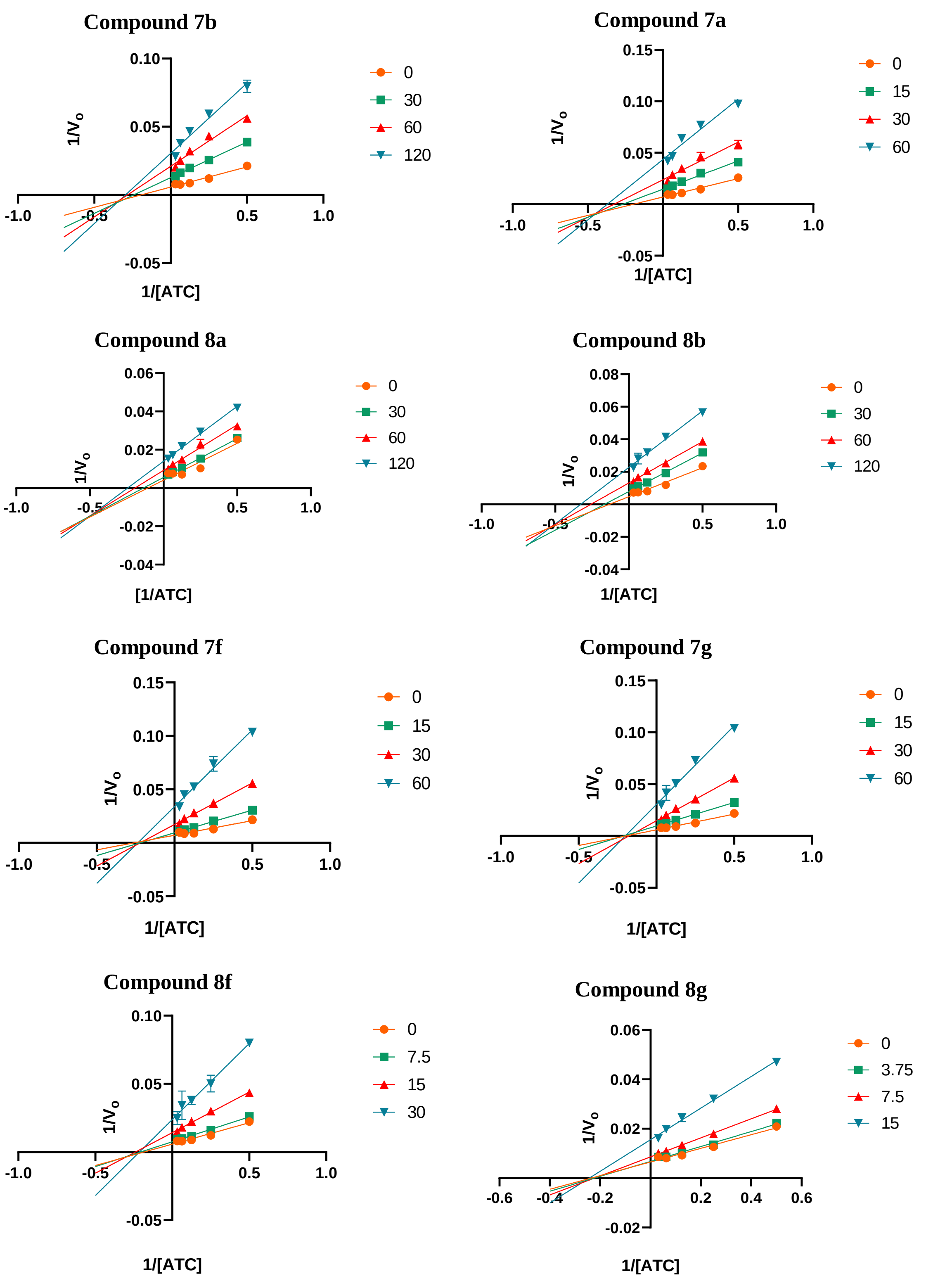

Figure S51-A: Lineweaver-Burk plots resulting from the substrate-velocity curve of AChE activity with different substrate (ATC) concentrations (32 – 2 nM) in the absence and presence of the most potent compounds.

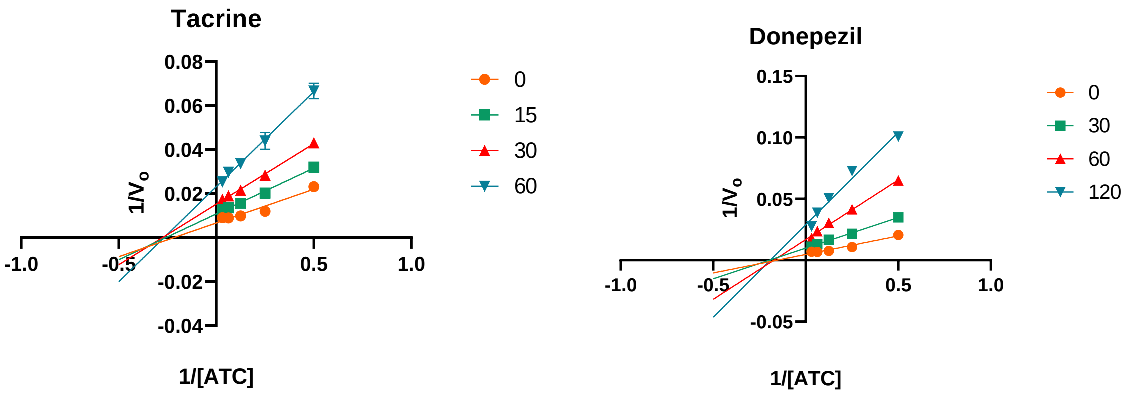

Figure S51-B: Lineweaver-Burk plots resulting from the substrate-velocity curve of AChE activity with different substrate (ATC) concentrations (32 – 2 nM) in the absence and presence of the standard drugs.

Compound 7a

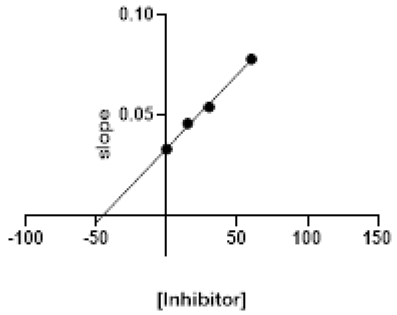

Compound 7b

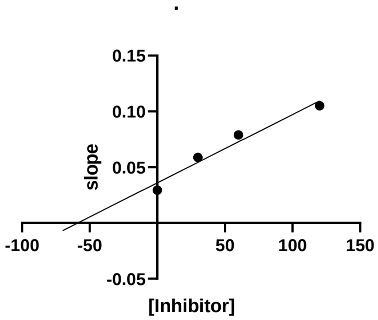

Compound 8a

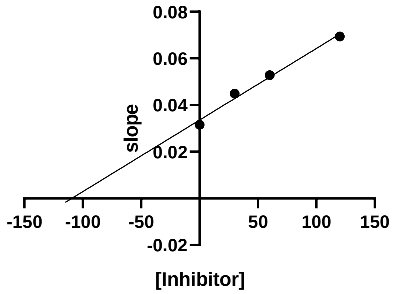

Compound 8b

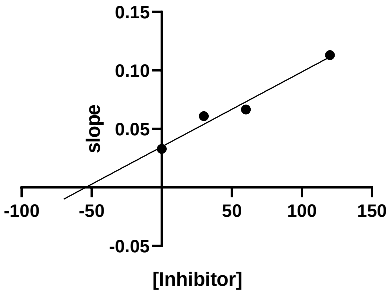

Compound 7f

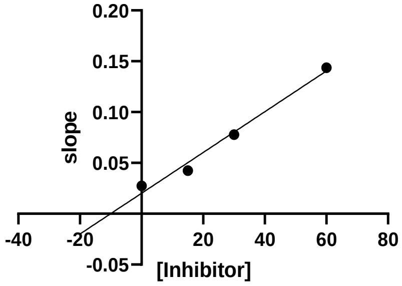

Compound 7g

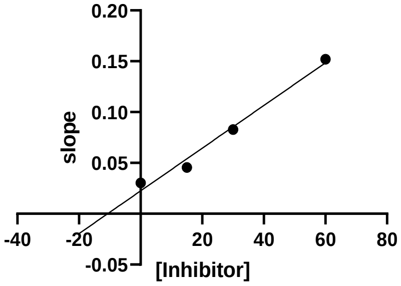

Compound 8f

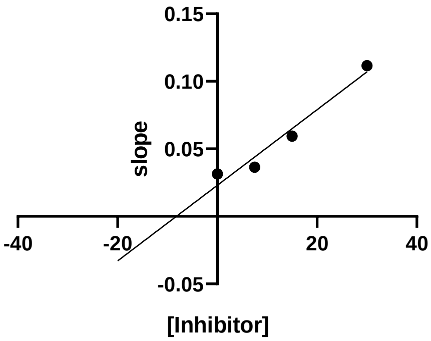

Compound 8g

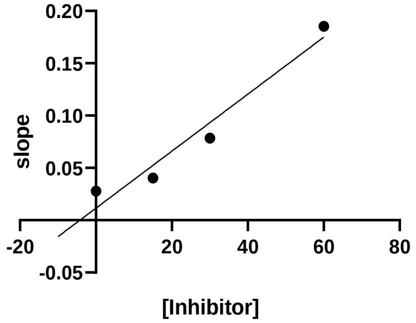

Figure S52-A: Lineweaver-Burk plot slopes versus concentration of the most potent inhibitors.

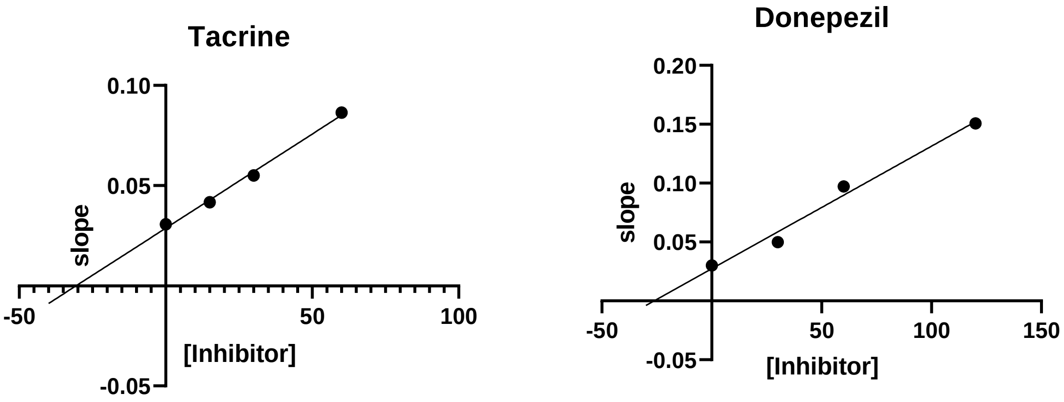

Figure S52-B: Lineweaver-Burk plot slopes versus concentration of the standard drugs.
